# A Phase 1 Randomized, Double Blind, Placebo Controlled Rectal Safety and Acceptability Study of Tenofovir 1% Gel (MTN-007)

**DOI:** 10.1371/journal.pone.0060147

**Published:** 2013-04-03

**Authors:** Ian Mcgowan, Craig Hoesley, Ross D. Cranston, Philip Andrew, Laura Janocko, James Y. Dai, Alex Carballo-Dieguez, Ratiya Kunjara Na Ayudhya, Jeanna Piper, Florian Hladik, Ken Mayer

**Affiliations:** 1 University of Pittsburgh School of Medicine, Pittsburgh, Pennsylvania, United States of America; 2 Microbicide Trials Network, Magee-Womens Research Institute, Pittsburgh, Pennsylvania, United States of America; 3 University of Alabama, Birmingham, Alabama, United States of America; 4 FHI 360, Research Triangle Park, Durham, North Carolina, United States of America; 5 University of Washington, Seattle, Washington, United States of America; 6 Statistical Center for HIV/AIDS Research and Prevention, Fred Hutchinson Cancer Research Center, Seattle, Washington, United States of America; 7 Columbia University, New York, New York, United States of America; 8 Division of AIDS, National Institute of Allergy and Infectious Diseases, National Institutes of Health, Bethesda, Maryland, United States of America; 9 Fenway Health, Boston, Massachusetts, United States of America; Wits Reproductive Health and HIV Institute, South Africa

## Abstract

**Objective:**

Rectal microbicides are needed to reduce the risk of HIV acquisition associated with unprotected receptive anal intercourse. The MTN-007 study was designed to assess the safety (general and mucosal), adherence, and acceptability of a new reduced glycerin formulation of tenofovir 1% gel.

**Methods:**

Participants were randomized 1∶1:1∶1 to receive the reduced glycerin formulation of tenofovir 1% gel, a hydroxyethyl cellulose placebo gel, a 2% nonoxynol-9 gel, or no treatment. Each gel was administered as a single dose followed by 7 daily doses. Mucosal safety evaluation included histology, fecal calprotectin, epithelial sloughing, cytokine expression (mRNA and protein), microarrays, flow cytometry of mucosal T cell phenotype, and rectal microflora. Acceptability and adherence were determined by computer-administered questionnaires and interactive telephone response, respectively.

**Results:**

Sixty-five participants (45 men and 20 women) were recruited into the study. There were no significant differences between the numbers of ≥ Grade 2 adverse events across the arms of the study. Likelihood of future product use (acceptability) was 87% (reduced glycerin formulation of tenofovir 1% gel), 93% (hydroxyethyl cellulose placebo gel), and 63% (nonoxynol-9 gel). Fecal calprotectin, rectal microflora, and epithelial sloughing did not differ by treatment arms during the study. Suggestive evidence of differences was seen in histology, mucosal gene expression, protein expression, and T cell phenotype. These changes were mostly confined to comparisons between the nonoxynol-9 gel and other study arms.

**Conclusions:**

The reduced glycerin formulation of tenofovir 1% gel was safe and well tolerated rectally and should be advanced to Phase 2 development.

**Trial Registration:**

ClinicalTrials.gov NCT01232803.

## Introduction

Rectal microbicides (RM) are currently being developed to prevent or at least significantly reduce the risk of HIV acquisition associated with unprotected receptive anal intercourse (RAI) [1]. RAI is a common sexual practice among men who have sex with men (MSM) [2]. Recent epidemiological data have suggested that RAI is also common among men and women in the developed and developing world [3]–[6]. As a consequence there is an urgent need to develop a safe and effective RM. Attention is currently focused on the development of tenofovir (TFV) gel as a potential RM. The vaginal formulation of TFV that was used in the CAPRISA 004 study [7] has been evaluated in a Phase 1 rectal safety study (RMP-02/MTN-006) [8]. Use of the gel was associated with mild to moderate gastrointestinal symptoms including bloating, pain, urgency, and diarrhea. The vaginal formulation of TFV is hyperosmolar (3111 mOsmol/kg) and it is possible that these symptoms were linked to product osmolality [9]. Consequently, the TFV used in the MTN-007 study was formulated with a lower glycerin concentration (5% w/w mg rather than the 20% w/w in the vaginal formulation) that results in a product osmolality of 836 mOsmol/kg [10]. It was anticipated this formulation would be better tolerated by study participants.

In the two phase 1 trials of antiretroviral rectal microbicides conducted to date, product use was not associated with any significant change in mucosal safety parameters [8], [11]. Whilst this is reassuring, the possibility exists that the range of parameters used in these studies was too narrow and might have missed unanticipated or subtle but important mucosal changes. To mitigate this situation, the MTN-007 study included microarray assessment of mucosal gene expression. In addition, a nonoxynol-9 (N-9) arm was included to help determine the utility of individual mucosal safety assays in detecting mucosal injury. Rectal use of N-9 in humans has been associated with transient mild gastrointestinal discomfort as well as minor histological abnormality [12] and has been associated with induction of proinflammatory responses in cervical epithelial cells [13]. It was hoped that these additional assessments would help provide a more comprehensive assessment of mucosal safety.

## Materials and Methods

The protocol for this trial and supporting CONSORT checklist are available as supporting information; see Checklist S1 and Protocol S1.

### Ethics Statement

The study was designed by the investigators with collaborative input from CONRAD and the NIAID/DAIDS/Prevention Sciences Integrated Preclinical-Clinical Program (IPCP) for HIV Topical Microbicides, as stipulated in the award notice and reviewed by the U.S. Food and Drug Administration (FDA). The study was approved by the University of Pittsburgh Institutional Review Board (IRB) as well as the University of Alabama IRB and the Fenway Health IRB. All subjects provided written informed consent. The trial is registered at ClinicalTrials.gov, number #NCT01232803 and is in compliance with the CONSORT 2010 recommendations for reporting of trial results (www.consort-statement.org) [14], [15].

### Study Schema

The primary objective of MTN-007 was to evaluate the safety of TFV gel when applied rectally. Secondary objectives included evaluation of the acceptability of TFV gel, the safety of the hydroxyethyl cellulose (HEC) placebo gel, and determining whether the use of either TFV or N-9 gels was associated with rectal mucosal damage. MTN-007 was a Phase 1, double blind, placebo-controlled trial in which participants were randomized to receive rectal TFV, N-9, HEC gels or No Rx (1∶1:1∶1) at three clinical sites (Pittsburgh, PA; Birmingham, AL; and Boston, MA). The study protocol was approved by IRBs at all three sites and informed consent was received from all participants. Enrollment began in October 2010 and the last participant completed the study in July 2011. The target sample size is 60, equally split to 15 in each arm, which ensures a 79% probability to observe at least one Grade 2 or higher adverse event in an arm when the true event rate is 10%. Five additional enrollees were recruited to preserve the study power in the case of participants using less than 5 doses in the 7-day dosing period or fail to complete the final clinical visit. A blinded statistician from The Statistical Center for HIV/AIDS Research & Prevention (SCHARP), University of Washington, Seattle, WAS, USA created lists containing randomly generated unique three-digit codes for study product randomization for each clinical site. Participants, study staff, pharmacists, clinicians and statisticians were blinded to study assignments.

### Study Population

The study population consisted of healthy, RAI-abstinent, HIV-uninfected, adults (male and female) aged 18 or older at time of screening. Female participants were required to be using an acceptable form of contraception (e.g., barrier method, intra uterine device, hormonal contraception, surgical sterilization, or vasectomization of the male partner). Individuals with abnormalities of the colorectal mucosa, significant gastrointestinal symptoms (such as a history of rectal bleeding), evidence of anorectal *Chlamydia trachomatis* (CT) or *Neisseria gonorrhea* (GC) infection, chronic hepatitis B infection, or a requirement to use drugs that were likely to increase the risk of bleeding following mucosal biopsy were excluded from the study.

### Study Products

Reduced glycerin (RG)-TFV 1% gel was supplied by CONRAD (Arlington, VA, USA). 2% N-9 was provided as Gynol II® (Johnson & Johnson, Fort Washington, PA). HEC gel, known as the “Universal Placebo Gel” [16], and used in a previous Phase 1 rectal safety study of the UC781 gel [11], was also supplied by CONRAD (Arlington, VA, USA). Each participant was assigned a carton of applicators, based on the randomization number. At the Treatment 1 Visit the participant’s first dose of study product was administered by the clinic staff. During the period of daily administration study participants were instructed to insert one dose of gel into the rectum once daily throughout the 7-day period. Rectal administration of study product occurred in the evening or before the longest period of rest. All study products were provided in identical opaque HTI polypropylene pre-filled applicators (HTI Plastics, Lincoln, NE) containing 4 mL of study product.

### Study Procedures

There were a total of five study visits and two follow-up phone calls. After obtaining informed consent all participants were screened with a thorough medical history, a targeted physical examination, a digital rectal examination, and collection of swabs for CT*/*GC nucleic acid amplification testing (NAAT). Urine was also collected for CT/GC NAAT and for pregnancy testing in the female participants (pregnancy testing was repeated at all subsequent clinical visits). Blood was collected for safety labs (complete blood count, urea nitrogen, creatinine, alanine aminotransferase, and aspartate aminotransferase) and serology (syphilis, HIV-1, hepatitis B, and herpes simplex 1 and 2). Participants who met the inclusion and exclusion criteria during the Screening Visit proceeded to an Enrollment Visit. At the Enrollment Visit participants were randomized, a behavioral questionnaire was administered and a rectal examination and a focused physical examination were performed. Swabs were collected for assessment of rectal microflora and quantification of cytokines/chemokines in rectal secretions. Participants then received a Normosol-R pH 7.4 enema and effluent was collected for evidence of epithelial sloughing and a sample of feces collected for measurement of fecal calprotectin. A flexible sigmoidoscope was then inserted into the rectum and 7 biopsies were collected at 15 cm from the anal margin. A disposable anoscope was inserted into the anal canal and high resolution anoscopy (HRA) of the anorectum was performed at 16× magnification with collection of 7 rectal biopsies at 9 cm from the anal margin. Biopsies were used for histology, qRT-PCR, microarray analysis, and flow cytometry. At the Treatment 1 Visit (performed within 7–28 days of the Enrollment visit), all participants randomized to receive gel product had a single applicator of study gel inserted into the rectum. Within 30 minutes, swabs were collected for microflora and cytokines. An enema was administered and the same rectal samples, including biopsies, were collected as occurred during the Enrollment Visit. At the Treatment 2 Visit (performed at least 7 days after Treatment Visit 1) participants randomized to receive gel product were provided with 7 applicators of study product to take home and asked to insert the contents of one applicator daily for 7 days. The Final Clinic Visit occurred no more than 21 days after Treatment Visit 2 and was identical to the Enrollment Visit except that anogenital testing (CT/GC) was only performed if clinically indicated.

### Clinical Safety and Laboratory Assessments

Emergent adverse events (AEs) were graded using the Division of AIDS Table for Grading the Severity of Adult and Pediatric Adverse Events, Version 1.0, December 2004 as well as Addendum 1 and 3 (*Female Genital and Rectal Grading Table for Use in Microbicide Studies* (http://rsc.tech-res.com/safetyandpharmacovigilance/). In cases where an AE was covered in both tables, the *Female Genital or Rectal Grading Table for Use in Microbicide Studies* was the grading scale utilized.

### Product Acceptability

Overall product like (or dislike) and likelihood of gel use in the future were assessed using an internet based computer assisted self-interview (CASI). To monitor adherence, participants were asked to use a phone reporting system after each episode of gel use.

### Mucosal Safety

#### Histology

A qualitative scoring system developed for inflammatory bowel disease (IBD) research [17] and adapted for use in rectal microbicide trials [18] was used to characterize potential product associated injury with a scale of 1 (normal) to 5 (mucosal erosion or ulceration).

#### Fecal calprotectin

Fecal calprotectin was measured using a commercial assay (Genova Diagnostics, Asheville, NC, USA) [19].

#### Epithelial sloughing

Epithelial sloughing was evaluated using a modification of a previously described technique [20]. Briefly, lavage fluid, collected following the Normosol enema was spun at 1000 rpm for 5 minutes. The cell pellet was resuspended in 1 mL of 2% paraformaldehyde. The suspension was placed in a petri dish that had previously been marked with quadrants. Each quadrant of the petri dish was scanned with a dissecting microscope at a magnification of 40×. The total number of >2 mm epithelial sheets in each quadrant of the petri dish were recorded.

#### Rectal microflora

Rectal microflora were characterized using previously described semi-quantitative culture analysis techniques [21], [22] that have been used in other rectal microbicide Phase 1 studies [8], [11].

#### Mucosal T cell phenotype

Mucosal mononuclear cells were isolated from rectal biopsies using a combination of mechanical and enzyme digestion as previously described [11]. Flow cytometric analysis was performed on a BD™ LSRFortessa cytometer (BD Biosciences, San Jose, CA). All data were stored in list mode and analyzed with BD™ FACSDIVA operating system and Flow Jo (Tree Star, Inc., Ashland, OR). All antibodies were purchased from BD Biosciences, San Jose, CA (PerCP-CD45, Clone 2D1; Pacific Blue-CD3, Clone UCHT1; PE-Cy7-CD4, Clone SK3; APC-H7-CD8, Clone SK1; FITC-CD69, Clone FN50; APC-CD184 (CXCR4), Clone 12G5 and PE-CD195 (CCR5)) and titrated under assay conditions to determine an optimum saturating dilution. Cells were first stained with LIVE/DEAD® Fixable Aqua stain fluorescence (Life Technologies, Eugene, OR).

#### Cytokine and chemokine mRNA expression

Rectal biopsies were homogenized in the presence of 0.5 mm RNase-free zirconium beads with the aid of a Bullet Blender homogenizer (Next Advance Inc., Averil Park, NY). RNA was then purified on columns using an RNAqueous®-4PCR kit (Applied Biosystems/Ambion, Foster City, CA). The extracted total RNA was eluted in a volume of 100 µl and DNase 1- treated. 1000 ng of total RNA from biopsy samples were converted to cDNA using MultiScribe™ reverse transcriptase and TaqMan® Reverse Transcription reagents (Applied Biosystems, Roche Molecular Systems, Inc., Branchburg, NJ). Oligo dT(20) was used to prime the reverse transcription (RT) reaction and reactions were run on the Applied Biosystems Veriti™ Dx Thermal Cycler (Life Technologies Corporation, Carlsbad, CA). Three reference genes (GAPDH, β-actin, and β2 Microglobulin β2M) were used to normalize mucosal gene expression of CD45, IL-1β, IL-6, IL-12p40, IL-8, IL-17, IFN-γ, MIP-1α, MIP-1β, TNF-α, IL23, CCR5 and RANTES (Table S1). All qRT-PCR experiments were performed on a Bio-Rad CFX96 Real Time PCR System (Bio-Rad, Hercules, CA).

#### Luminex analysis of rectal secretions

Luminex® was used to measure IFN-γ, IL-1β, IL-6, IL-8, IL-12 (p40), IL-17, MIP-1α, MIP-1β, RANTES and TNF-α in rectal secretions (MILLIPLEX MAP kit; Millipore, Billerica, MA).

#### Microarray analysis

RNA was extracted from colorectal biopsies taken from 32 male study participants (8 men per study arm, including the no-treatment arm) using the RNeasy Mini Kit (Qiagen, Valencia, CA). 200 ng of total RNA was amplified and labeled using the Illumina TotalPrep RNA Amplification kit (Ambion, Grand Island NY). cRNA was hybridized to HumanHT12 v4 Expression BeadChips (Illumina Inc., San Diego, CA).

### Analysis of Outcomes

Adverse events were evaluated for the full study cohort that included all participants that were randomized into the study. All adverse events were classified using the MedDRA organ system class/preferred term. The proportion of participants having at least one AE event was compared across arms using the Chi Square test or Fisher’s exact test when counts are small. Product acceptability was determined through a CASI interview and was operationalized as intentionality of product use with RAI having a rating in the upper one third of a 10-point Likert scale (values of 7–10). Histology, fecal calprotectin, epithelial sloughing, rectal microflora, and mucosal biomarkers were summarized for each arm by mean and standard deviation among participants who completed at least 5 doses of gels during the 7-day product use period and completed the Final Clinic Visit. Using linear regression, pairwise group comparisons were conducted across groups after adjustment for baseline variability for biomarkers measured after the single-dose use and for the 7-day use, separately. Multiple testing in mucosal biomarkers was adjusted for by using Bonferroni correction to control family wise error rate, as well as using the Benjamini-Hochberg procedure to control false discovery rate (FDR) [23]. Microarray data were pre-processed using robust spline normalization and variance stabilizing transformation. Unexpressed or low variability probes were filtered out, leaving 1928 probes for analysis of treatment effects versus control using a Bayesian statistical framework Cyber-T and Benjamini-Hochberg false discovery rate (FDR) significance adjustment [23]. Criteria for significance and relevance were an FDR ≤0.05 and a log_2_ fold expression change of ≥0.5 (up-regulation) or ≤0.5 (down-regulation), respectively.

## Results

### Enrollment, Retention, and Participant Disposition

**Figure 1:**
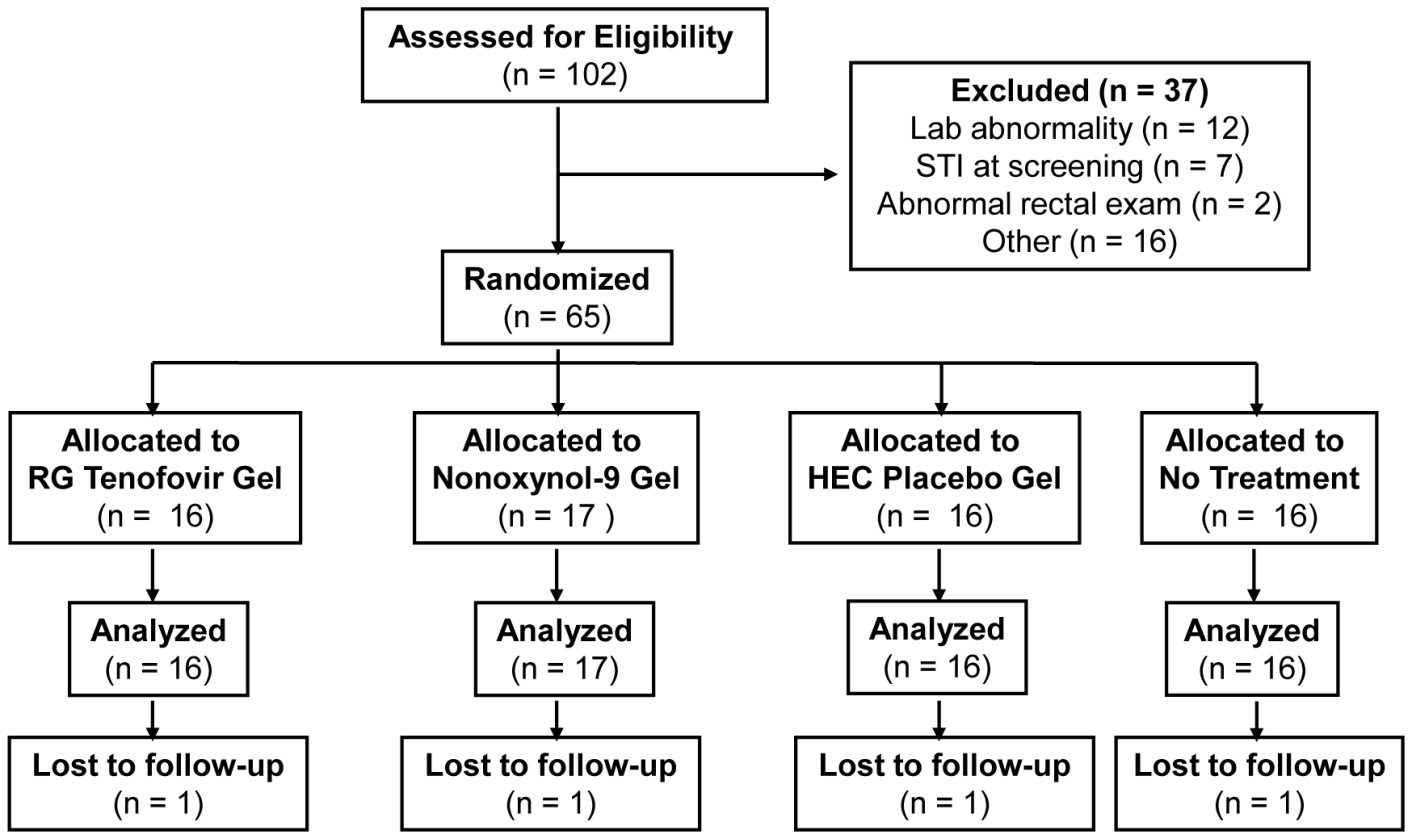
Flow diagram of participant progress through the MTN-007 study.

A total of 65 participants were enrolled and randomized in the study (TFV, *n* = 16; N-9, *n* = 17; HEC, *n* = 16; and No Rx, *n* = 16) (Figure 1). The majority of participants were white males (Table 1). There is no statistical difference between arms in age distribution (p-value 0.79, ANOVA test), in gender composition (p-value 0.75, Fisher’s exact test), nor in the proportion of white participants (p-value 0.54, Chi-square test). One participant in the N-9 arm withdrew from further participation after the enrollment visit (before receiving any study product), due to an unrelated adverse event. After completing their single-dose exposure, two participants did not receive products for the 7-day exposure. One participant in the HEC group had major depression, was placed on product hold, and was subsequently removed from the study. The other participant in the TFV arm missed the Treatment Visit 2. Averaged across all study visits, the proportion of participants who completed expected visits was 95% or above for all four study arms.

**Table 1:** Baseline demographics of each treatment group.

		All Arms	Tenofovir Gel	N-9 Gel	HEC Placebo Gel	No Treatment
**Participants Enrolled**		65	16	17	16	16
**Age (Years)**	**Mean (STD)**	35.7 (11.0)	35.3 (8.7)	37.0 (9.9)	36.8 (12.6)	33.5 (13.0)
	**p-value**	0.79				
**Gender**	**Male**	45 (69%)	10 (63%)	13 (76%)	12 (75%)	10 (63%)
	**Female**	20 (31%)	6 (38%)	4 (24%)	4 (25%)	6 (38%)
	**p-value**	0.75				
**Race**	**White**	44 (68%)	10 (63%)	13 (76%)	9 (56%)	12 (75%)
	**Non-white**	21 (32%)	6 (37%)	4 (24%)	7 (44%)	4 (25%)
	**p-value**	0.54				

HEC, hydroxyethyl cellulose; N-9, Nonoxynol-9.

### Adherence and Acceptability

Among 46 participants who received products for the 7-day use of gel, two participants had at least one product hold but later resumed product use; one was due to moderate elbow cellulitis after the Treatment visit 1 (N-9) and the other was due to prohibited medication. Accounting for these product holds, only 1 participant in the N-9 arm reported using less than 80% of the assigned doses between the Treatment Visit 2 and Final visit (Table S2). Among 46 participants who were assigned to use gel and completed the acceptability question, reported likelihood of future product use (acceptability) was 93% (HEC), 87% (TFV, Fisher exact test p-value = 1.0 when compared to the HEC group), and 63% (N-9, Fisher exact test p-value = 0.08 when compared to the HEC group).

### Adverse Events

Adverse events were generally mild (Grade 1, N = 121, 80% of all AEs) or moderate (Grade 2, N = 27, 18% of all AEs). Two Grade 3 events occurred in the No Rx arm and one Grade 4 psychiatric event (the major depression episode mentioned above) occurred in the HEC arm prior to 7-day product use; all Grade 3 and 4 AEs were unrelated to product. Gastrointestinal adverse events were common but the vast majority were mild (Table 2). There were no significant differences in the proportion of participants with ≥ Grade 2 or higher adverse events across the arms of the study: 3/16 (19%) in the TFV arm, 7/17 (41%) in the N-9 arm, 5/16 (31%) in the HEC arm, and 6/16 (38%) in the No Rx arm; when compared to the No Rx arm, one sided Fisher exact test yielded p-value 0.94 for the TFV arm, 0.56 for the N-9 arm, and 0.77 for the HEC arm.

**Table 2:** Participants reporting adverse events by study arm.

	All Arms	Tenofovir Gel	N-9 Gel	HEC Placebo Gel	No Treatment
**Participants enrolled**	65	16	17	16	16
**Total number of AEs**	151	32	51	34	34
**Grade 1 (Mild)**	121 (80.1%)	29 (90.6%)	44 (86.3%)	26 (76.5%)	22 (64.7%)
**Grade 2 (Moderate)**	27 (17.9%)	3 (9.4%)	7 (13.7%)	7 (20.6%)	10 (29.4%)
**Grade 3 (Severe)**	2 (1.3%)	0	0	0	2 (5.9%)
Grade 4 (Potentially life-threatening)*	1 (0.7%)	0	0	1 (2.9%)	0
**Grade 5 (Death)**	0	0	0	0	0
**Participants with one or more AEs**					
**Grade 1 (Mild)**	30 (46.2%)	7 (43.8%)	10 (58.8%)	7 (43.8%)	6 (37.5%)
**Grade 2 (Moderate)**	18 (27.7%)	3 (18.8%)	7 (41.2%)	4 (25.0%)	4 (25.0%)
**Grade 3 (Severe)**	2 (3.1%)	0	0	0	2 (12.5%)
**Grade 4 (Potentially life-threatening)**	1 (1.5%)	0	0	1 (6.3%)	0
**Grade 5 (Death)**	0	0	0	0	0
**Participants with gastrointestinal AEs**					
**Abdominal distension (G1/G2)**	5 (7.7%)	1 (6.3%)	2 (11.8%)	2 (12.5%)	0
**Abdominal pain (G1)**	3 (4.6%)	1 (6.3%)	0	1 (6.3%)	1 (6.3%)
**Anal pruritus (G1)**	3 (4.6%)	1 (6.3%)	1 (5.9%)	1 (6.3%)	0
**Defecation urgency (G1)**	6 (9.2%)	0	5 (29.4%)	1 (6.3%)	0
Diarrhea (G1/G2/G3**)	9 (13.8%)	1 (6.3%)	4 (23.5%)	1 (6.3%)	3 (18.8%)
**Flatulence (G1/G2)**	13 (20%)	6 (37.5%)	2 (11.8%)	2 (12.5%)	3 (18.8%)
**Haematochezia (G1)**	1 (1.5%)	0	0	1 (6.3%)	0
**Haemorrhoids (G1)**	2 (3.1%)	0	1 (5.9%)	1 (6.3%)	0
**Intestinal polyp (G1)**	1 (1.5%)	0	0	0	1 (6.3%)
**Painful defecation (G1)**	2 (3.1%)	0	2 (11.8%)	0	0
**Proctalgia (G1)**	3 (4.6%)	1 (6.3%)	2 (11.8%)	0	0
**Proctitis (G1)**	1 (1.5%)	0	1 (5.9%)	0	0
**Rectal polyp (G1)**	3 (4.6%)	1 (6.3%)	1 (5.9%)	0	1 (6.3%)

HEC, hydroxyethyl cellulose; N-9, Nonoxynol-9; AEs, adverse events, *Grade 4 event was a participant who experienced a psychiatric AE (major depression) that occurred prior to 7-day product use **Grade 3 diarrhea occurred in 1 participant in the No Treatment arm.

### Mucosal Safety

#### Histology, fecal calprotectin, and epithelial sloughing

There was suggestive evidence of increase in the histology scores for the N-9 and HEC arms compared to the TFV arm at the 9 cm site at the Final Visit (Table 3). In contrast, there were no significant differences between the fecal calprotectin levels or epithelial sloughing scores (data not shown).

**Table 3:** Mucosal immunology assays with suggestive evidence of changes with nominal pairwise comparison p-value less than 0.05.

		TenofovirGel (T)	N-9 Gel(N9)	HEC PlaceboGel (H)	No Treatment(N)	p-Value
**Participants analyzed**		15	15	15	15	
		Mean (SD)	Mean (SD)	Mean (SD)	Mean (SD)	
**Histology score** **at 9 cm**	**Enrollment**	0.7 (0.5)	1.0 (0.0)	1.1 (0.6)	0.9 (0.4)	
	**Treatment 1**	1.1 (0.6)	1.1 (0.7)	1.4 (0.8)	1.5 (1.0)	
	**Final visit**	0.7 (0.5)	1.8 (1.2)	1.1 (0.6)	1.4 (0.6)	T:N9, 0.01, N9:H, 0.03
**Flow cytometry** **at 9 cm**						
	**Final Visit**					
	CD45/CD3 (%)	51.7 (19.3)	48.5 (17.4)	41.0 (15.9)	57.7 (14.8)	N9:H, 5.7^−3^
	CD3/CD4 (%)	60.5 (9.0)	60.5 (7.9)	53.8 (10.8)	50.1 (11.1)	N9:H, 0.04; N9:N, 0.01
	CD4/CXCR4 (%)	48.0 (20.4)	59.6 (20.8)	49.4 (25.6)	38.2 (25.7)	N9:N, 0.02
	CD3/CD8 (%)	27.4 (8.6)	27.5 (7.6)	31.2 (8.6)	37.3 (10.4)	N9:N, 1.6^−3^ ˆ
	CD8/CD69 (%)	71.3 (14.1)	69.5 (8.8)	75.0 (12.8)	83.4 (9.8)	N9:N, 0.02
**Flow cytometry** **at 15 cm**						
	**Treatment 1**					
	CD8/CD69 (%)	81.8 (9.1)	80.5 (14.3)	82.5 (11.5)	89.0 (5.4)	T:N, 0.02
	**Final visit**					
	CD45/CD3 (%)	57.3 (10.6)	47.2 (16.0)	42.8 (15.5)	50.8 (18.9)	T:H, 0.04
	CD3/CD8 (%)	31.2 (8.5)	32.8 (10.8)	30.3 (8.7)	36.5 (12.8)	N9:N, 0.04
	CD8/CCR5 (%)	90.0 (6.0)	84.7 (8.7)	80.3 (15.4)	80.8 (10.7)	T:N, 0.01
**qRT-PCR** + **at 9 cm**						
	**Treatment 1**					
	IL-1β	−2.9 (0.6)	−2.2 (0.8)	−2.9 (0.8)	−3.2 (0.6)	N9:H, 6.8^−4^ ˆ; N9:N,1.3^−3^ ˆ
	IL-6	−4.2 (0.7)	−3.1 (0.6)	−4.0 (0.8)	−4.3 (1.1)	N9:T, 1.3^−5^ * ˆ; N9:H, 3.0^−4^ ˆ; N9:N, 4.3^−3^
	TNF-α	−3.3 (0.9)	−2.8 (0.7)	−3.5 (0.9)	−3.8 (0.5)	N9:H, 0.01; N9:N, 2.8^−3^
	IL-8	−3.0 (0.7)	−2.1 (0.9)	−3.0 (0.8)	−3.2 (0.7)	N9:T, 4.2^−5^ * ˆ; N9:H, 2.8^−4^ ˆ; N9:N, 4.9^−3^
	MIP-1α	−3.4 (0.7)	−2.8 (0.7)	−3.5 (0.8)	−3.7 (0.6)	N9:T, 2.7^−4^ ˆ; N9:H, 2.5^−3^; N9:N, 2.6^−3^
	MIP-1β	−2.9 (0.8)	−2.5 (0.7)	−3.0 (0.8)	−3.3 (0.5)	N9:T, 1.8^−3^ ˆ; N9:H, 7.4^−3^; N9:N, 8.0^−3^
	IL-12p40	−4.9 (0.8)	−4.4 (0.8)	−5.0 (1.0)	−5.2 (0.7)	N9:N, 0.02
	IL-23	−4.4 (1.1)	−3.6 (1.0)	−3.9 (0.7)	−4.6 (0.9)	N9:H, 0.02; N9:N, 0.01
	CCR5	−4.6 (0.8)	−3.9 (0.6)	−4.1 (0.6)	−4.6 (0.7)	N9:H, 0.03; N9:N, 6.0^−3^
	**Final visit**					
	RANTES	−2.7 (0.9)	−2.5 (0.8)	−2.4 (0.5)	−2.6 (0.7)	N9:T, 0.02; N9:H, 0.03; N9:N, 0.02
	IL-17	−4.9 (1.0)	−4.4 (0.8)	−4.5 (0.6)	−4.9 (0.8)	T:N, 0.02
**qRT-PCR** + **at 15 cm**						
	**Treatment 1**					
	IFN-γ	−4.6 (0.6)	−4.4 (0.8)	−4.3 (0.6)	−4.7 (0.4)	N9:H, 0.04; N9:N, 0.04
	RANTES	−2.5 (0.9)	−2.1 (0.7)	−2.2 (0.6)	−2.5 (0.6)	T:N, 0.01
	IL-17	−4.8 (0.9)	−4.4 (0.9)	−4.4 (0.8)	−5.2 (0.5)	H:N, 0.02
	**Final visit**					
	IL-6	−4.5 (0.7)	−4.3 (0.7)	−4.0 (0.5)	−4.5 (0.7)	N9:H, 0.02
	MIP-1α	−3.4 (0.7)	−3.6 (0.7)	−3.6 (0.6)	−3.9 (0.6)	T:N, 0.04
	RANTES	−2.6 (1.0)	−2.2 (0.7)	−2.3 (0.5)	−2.5 (0.7)	N9:T, 0.01
	IL-12p40	−5.2 (0.9)	−4.8 (1.1)	−4.9 (0.7)	−5.2 (0.6)	T:H, 6.3^−3^; N9:H, 0.04
	IL-23	−4.5 (1.1)	−4.0 (1.1)	−3.9 (0.7)	−4.7 (1.0)	H:N, 7.5^−3^
**Luminex assay**						
	**Treatment 1**					
	IL-8	164.3 (229.9)	537.3 (421.9)	510.9 (570.9)	303.5 (498.4)	N9:T, 0.02
	MIP-1α	2.6 (1.1)	4.1 (3.1)	5.0 (5.2)	7.2 (7.7)	T:N, 0.03
	RANTES	8.3 (10.1)	335.9 (429.1)	12.1 (10.6)	33.1 (74.6)	N9:T, 3.6^−3^; N9:H, 1.3^−3^ ˆ; N9:N, 0.01
	**Final visit**					
	TNF-α	3.0 (4.0)	2.2 (1.9)	2.7 (2.1)	1.4 (1.0)	H:N, 0.04

HEC, hydroxyethyl cellulose; N-9, Nonoxynol-9, qRT-PCR, quantitative reverse transcriptase PCR, RANTES; Regulated upon activation normal T cell expressed.

+ Data expressed as logarithm (base 10) transformation of the number of copies of gene of interest/the number of copies of β2 microglobulin in the same sample.

* p-values that are significant after controlling for family-wise error rate at 0.05 using Bonferroni correction.

ˆp-values that are significant after controlling for false discovery rate at 0.1 using the Benjamini-Hochberg procedure.

#### Rectal microflora

There were no significant changes in rectal microflora between the Baseline and Final Visits in any of the gel arms (data not shown).

#### Mucosal T cell phenotype, cytokine and chemokine mRNA expression, and Luminex analysis of rectal secretions

Forty-five participants who completed more than 5 doses during the 7-day product use period as well as fifteen participants in the No Rx arm who completed the Final Clinic Visit were included in these biomarker analyses. Table 3 shows biomarkers that have pairwise comparison nominal p-values less than 0.05. Only two tests remain significant after Bonferroni multiple testing correction (the comparisons of N-9 versus TFV for IL-6 and IL-8 gene expression after the single-dose treatment). Eight more comparisons meet the more lenient false discovery rate cut-off 0.1 using the Benjamini-Hochberg procedure. The majority of these changes involved upregulation of cytokine/chemokine expression in the N-9 arm.

#### Microarray analysis

**Figure 2:**
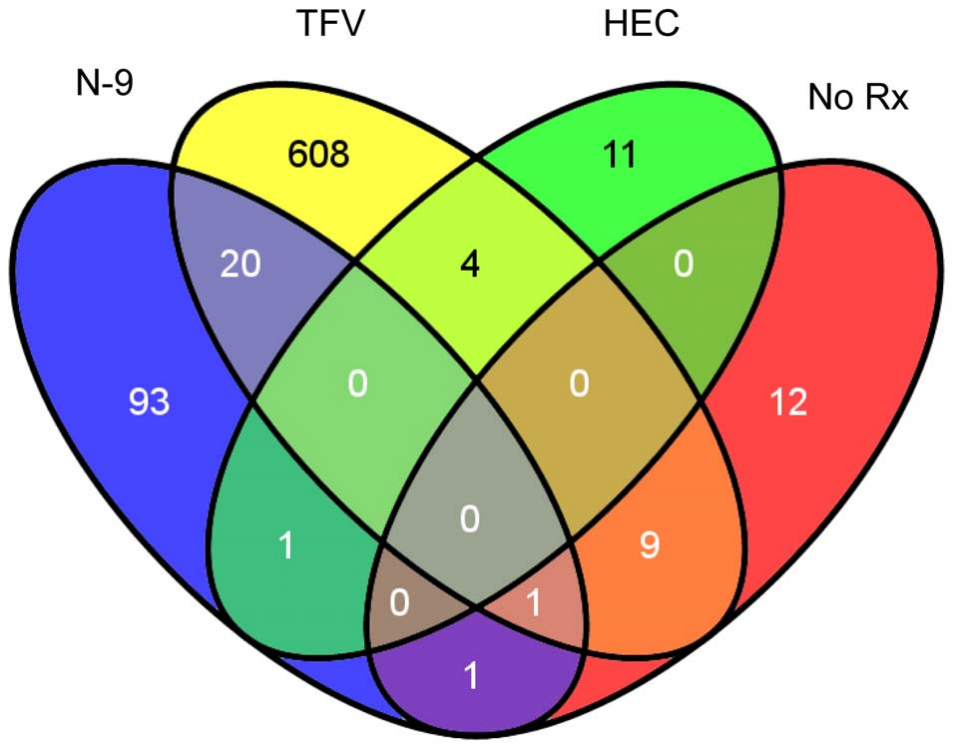
Number of genes in rectal biopsies (9 cm) with significant expression changes between baseline and after 7 days of treatment. The four ellipses represent each of the four treatment arms, Nonoxynol 9 (N-9) gel, tenofovir (TFV) gel, hydroxyethylcellulose (HEC) gel or no gel (No Rx). The numbers of genes changing expression exclusively in one study arm are indicated in fields of the diagram that do not overlap with another field (N-9, 93 genes; TFV, 608 genes; HEC, 11 genes; No Rx 12 genes). When identical genes changed in more than one treatment arm, their number is placed into the respective overlapping fields of the diagram. Choice of colors is arbitrary to help visually distinguish each separate field of the diagram. cRNA preparation, gene chip hybridization, and data significance analysis was carried out as described in the Materials and Methods section of the paper.

A single application of N-9 or TFV up-regulated 24 genes and 70 genes, and down-regulated 30 and 8 genes, respectively. At the Final Visit, N-9 up-regulated 60 and down-regulated 56 genes, whereas TFV up-regulated 137 and down-regulated 505 genes (Figure 2). Background fluctuations in the HEC and no-treatment arms were negligible (manuscript in preparation).

### Gender Specific and Regional Variations in Mucosal Biomarkers

MTN-007 enrolled 20 women (31% of the study population) all of whom reported RAI. Suggestive differences in T cell phenotype (CD8+/CD69+) and mucosal cytokine/chemokine gene expression (TNF-α and IL-23) were also present when the data were stratified by gender (manuscript in preparation). Regional differences were seen in T cell phenotype (CD3+/CD8+, CD8+/CD69+, CD4+/CD69+, CD4+/CXCR4+) and mucosal cytokine/chemokine gene expression (IL-1β, IL-6, IL-8, MIP-1α, MIP-1β, RANTES, IL-12p40, IL-17, and IL-23) between samples collected at 9 cm and 15 cm (manuscript in preparation).

## Discussion

This study demonstrates that the RG formulation of TFV gel is safe and acceptable when administered for up to seven days in sexually abstinent participants with a history of RAI. Gastrointestinal AEs such as abdominal pain, rectal urgency, diarrhea, and flatulence were the most common AEs seen but generally mild or moderate. The preparatory enema and subsequent flexible sigmoidoscopy may have contributed to these symptoms. However, the overall rates of these symptoms were less than those seen in the RMP-02/MTN-006 study which evaluated a more hyperosmolar formulation of TFV gel [8]. Use of TFV gel did not appear to be associated with mucosal damage as assessed by a broad range of histological, immunological, and microbiological parameters. Significant changes in gene expression were identified in the TFV gel arm using microarray technology. The biological significance of these changes, and more importantly whether they represent a mucosal safety signal, is not clear and will require further evaluation. A recent preclinical study identified intestinal injury associated with *in vitro* exposure of colorectal explants to the hyperosmolar vaginal formulation of TFV [9]. However, similar histopathological changes were not seen in the TFV gel arm of the MTN-007 study.

The inclusion of N-9 as a positive control was associated with evidence of mild mucosal injury but was not associated with significant changes in epithelial sloughing. Indeed epithelial sloughing was seen at baseline in several participants. Non-human primate studies of rectal exposure to N-9 also failed to demonstrate epithelial sloughing [24]. Fecal calprotectin was also not elevated throughout the study. This assay is useful in discriminating between irritable bowel syndrome and IBD [19] but appears to have limited utility in the evaluation of RM. Based on these data, the MTN will not use these two assays in future Phase 1/2 studies of candidate RM.

It is unclear whether the changes in mucosal gene expression assessed by microarray are related to the TFV or its associated formulation. Future RM studies may answer this question. The CHARM-01 study (NCT01575405) will explore the impact of three different TFV formulations on rectal safety and acceptability. Each formulation has a different osmolality ranging from 479 to 3111 mOsmol/kg) and the study should be able to evaluate the impact of osmolality on mucosal safety. The MTN-017 study (NCT01687218) will be a Phase-2 RM study in which participants will receive 8 weeks of TFV gel (either daily or with sex) followed by 8 weeks of oral Truvada®. This study will provide information on the consequences of extended exposure to rectal TFV as well as a crossover comparison of oral and topical exposure to TFV. Both studies are expected to start in 2013.

Importantly, 31% of the participants in MTN-007 were women; emphasizing the need for RM for both men and women. We observed some modest gender specific differences in mucosal safety biomarkers but a larger study would be required to provide more definitive data on the impact of gender on the gastrointestinal mucosa. Differences in mucosal safety parameters between the 9 cm and 15 cm samples were more marked and in keeping with previous studies demonstrating regional heterogeneity in colonic mucosal biology [18], [25].

This study demonstrated that it is possible to collect adequate mucosal samples for Phase 1 RM studies via anoscopy. This observation will potentially simplify the design and execution of future Phase 1 RM studies although it is difficult to collect more than 7 rectal biopsies via anoscopy and so studies requiring collection of rectal samples for mucosal safety, pharmacokinetic, and pharmacodynamic assessment may still require flexible sigmoidoscopy.

It is encouraging that the RG formulation of TFV is well tolerated as the vaginal formulation used in previous studies was associated with a high frequency of gastrointestinal side effects when given rectally [8]. This safety profile together with evidence from the RMP-02/MTN-006 study showing that rectal use of TFV is associated with *ex vivo/in vitro* inhibition of HIV-1 viral replication [8] provides a compelling rationale for progression of this product into Phase 2 development. Collectively, these data will determine the longer term safety profile of this product and help decide whether it is a suitable agent to take into Phase 3 effectiveness studies.

## Supporting Information

Table S1 **qRT-PCR primer and probe sequences.**
(DOC)

Table S2 **Study retention and adherence.**
(DOC)

Checklist S1 **MTN-007 CONSORT Checklist.**
(DOC)

Protocol S1 **MTN-007 Trial Protocol.**
(PDF)
