# Supplementary material for: A Phase 1 Randomized, Double Blind, Placebo Controlled Rectal Safety and Acceptability Study of Tenofovir 1% Gel (MTN-007)
Source: PLoS One. 2013 Apr 3;8(4):e60147. doi: 10.1371/journal.pone.0060147 (PMC3616022; doi:10.1371/journal.pone.0060147)
Supplement: Table S1 — qRT-PCR primer and probe sequences. (DOC) [file pone.0060147.s001.doc]

**Table S1. qRT-PCR Primer and Probe sequences**

| Gene Name | Primers | Sequence 5' to 3' | Length |
| --- | --- | --- | --- |
|  |  |  |  |
| 1) GAPDH | Forward | GCCTCAAGATCATCAGCAATG |  |
|  | Reverse | CTTCCACGATACCAAAGTTGTC |  |
|  | Probe | GCCAAGGTCATCCATGA | 89bp |
| 2) β-Actin | Forward | TGGAGAAAATCTGGCACCAC |  |
|  | Reverse | GGTCTCAAACATGATCTGG |  |
|  | Probe | ACCGCGAGAAGATGACC | 106bp |
| 3) β2M | Forward | CTTTGTCACAGCCCAAGATAG |  |
|  | Reverse | ATCCAAATGCGGCATCTTC |  |
|  | Probe | CAGCATCATGGAGGTTTG | 80bp |
| 4) CD45 | Forward | GGAAGTGCTGCAATGTGTCATT |  |
|  | Reverse | CTTGACATGCATACTATTATCTGATCTCA |  |
|  | Probe | ACAACTAAAAGTGCTCCTCCAAGCCAGGTCT | 101bp |
| 5) IL-1β | Forward | ACAGATGAAGTGCTCCTTCCA |  |
|  | Reverse | ATCCAGCTACGAATCTCCGAC |  |
|  | Probe | CTCTGCCCTCTGGATGGCGG | 73bp |
| 6) IL-6 | Forward | GGTACATCCTCGACGGCATCT |  |
|  | Reverse | GTGAAAGCAGCAAAGAGGCACT |  |
|  | Probe | AGCCCTGAGAAAGGAGACATGTAACAAGAGTAACA | 81bp |
| 7) IL-12p40 | Forward | TGGAGTGCCAGGAGGACAGT |  |
|  | Reverse | CAAACCTGACCCACCCAAGA |  |
|  | Probe | ATGGTGGATGCCGTTCACAAGCTCAA | 147bp |
| 8) IFN-γ | Forward | TCAGCTCTGCATCGTTTTGG |  |
|  | Reverse | TTCAGATGTAGCGGATAATGGAAC |  |
|  | Probe | TTGGCTGTTACTGCCAGGACCCATATGT | 120bp |
| 9) TNF-α | Forward | CCAGGCAGTCAGATCATCTTCTC |  |
|  | Reverse | AAGCTGAGGGGCAGCTCC |  |
|  | Probe | AGCCTGTAGCCCATGTTGTAGCAAACCC | 86bp |
| 10) IL-8 | Forward | CCACACTGCGCCAACA |  |
|  | Reverse | ATCCAAGAATCAGTGAAGATGC |  |
|  | Probe | CTGGGTGCAGAGGGTTGTGG | 168bp |
| 11) MIP-1α | Forward | GAGACGAGCAGCCAGTGCTC |  |
|  | Reverse | GCCGGCAGGTCTGTGC |  |
|  | Probe | CGGTGTCATCTTCCTAACCAAGCGA | 64bp |
| 11) MIP-1β | Forward | TCTCAGCACCAATGGGCTC |  |
|  | Reverse | TTCTTACACCGCGAGGAAGC |  |
|  | Probe | CCCTCCCACCGCCTGCTGCT | 63bp |
| RANTES | Forward | CTACACCAGTGGCAACTGCT |  |
|  | Reverse | AGAAGAAATGGGTTCGGGA |  |
|  | Probe | TCACCCGAAAGAACCGCCAAGTGT | 95bp |
