# Supplementary material for: A Phase 1 Randomized, Double Blind, Placebo Controlled Rectal Safety and Acceptability Study of Tenofovir 1% Gel (MTN-007)
Source: PLoS One. 2013 Apr 3;8(4):e60147. doi: 10.1371/journal.pone.0060147 (PMC3616022; doi:10.1371/journal.pone.0060147)
Supplement: Table S2 — Study retention and adherence. (DOC) [file pone.0060147.s002.doc]

**Table S2. Study retention and adherence by treatment group.**

|  | **All Arms** | **Tenofovir Gel** | **N-9 Gel** | **HEC Placebo Gel** |
| --- | --- | --- | --- | --- |
| **Participants enrolled** | 65 | 16 | 17 | 16 |
|  |  |  |  |  |
| **Study retention for all visits** | 98%* | 100% | 100% | 100% |
|  |  |  |  |  |
| **Randomized and received study product** | 46 | 15 | 16 | 15 |
|  |  |  |  |  |
| **Randomized but did not receive study product** | 2 | 1 | 0 | 1 |
|  |  |  |  |  |
| **Percentage of study product use (Visit 2 to Final Visit)** |  |  |  |  |
| 0.0% | 0 (0%) | 0 (0%) | 0 (0%) | 0 (0%) |
| 0.1 – 49.9% | 1 (2%) | 0 (0%) | 0 (0%) | 0 (0%) |
| 50.0 – 79.9% | 0 (0%) | 0 (0%) | 0 (0%) | 0 (0%) |
| >= 80.0% | 45 (98%) | 15 (100%) | 15 (94%) | 15 (100%) |
| **Product hold or discontinuation** | 3 | 1 | 1 | 1 |
| **Reasons for product hold or discontinuation** |  |  |  |  |
| Adverse experience | 2 (67%) | 0 (0%) | 1 (100%) | 1 (100%) |
| Other | 1 (33%) | 1 (100%) | 0 (0%) | 0 (0%) |
|  |  |  |  |  |
| **Product resumed** |  |  |  |  |
| Yes | 2 (67%) | 1 (100%) | 1 (100%) | 0 (0%) |
| No | 1 (33%) | 1 (100%) | 1 (100%) | 1 (100%) |

HEC, hydroxyethylcellulose; N-9, Nonoxynol-9

*94% in the no treatment arm
