# Supplementary material for: A Phase 1 Randomized, Double Blind, Placebo Controlled Rectal Safety and Acceptability Study of Tenofovir 1% Gel (MTN-007)
Source: PLoS One. 2013 Apr 3;8(4):e60147. doi: 10.1371/journal.pone.0060147 (PMC3616022; doi:10.1371/journal.pone.0060147)
Supplement: Protocol S1 — MTN-007 Trial Protocol. (PDF) [file pone.0060147.s004.pdf]

MTN-007
A Phase 1 Randomized, Double-Blinded, Placebo-Controlled Rectal Safety and Acceptability Study of Tenofovir 1% Gel

A Study of the Microbicide Trials Network

Funded by:
Division of AIDS, US National Institute of Allergy and Infectious Diseases
National Institute of Mental Health
US National Institutes of Health

Grant #:
5-U01-AI068633-05

DAIDS Protocol #: 10736

IND Holder:
CONRAD

This protocol will be performed under CONRAD IND#: 73,382

Protocol Chair:
Ian McGowan, MD, PhD, FRCP

Protocol Co-Chair:
Kenneth Mayer, MD

Version 2.0

August 13, 2010

MTN-007

A Phase 1 Randomized, Double-Blinded, Placebo-Controlled Rectal Safety and Acceptability Study of Tenofovir 1% Gel

TABLE OF CONTENTS

LIST OF ABBREVIATIONS AND ACRONYMS i
PROTOCOL TEAM ROSTER v
INVESTIGATOR SIGNATURE FORM ix
PROTOCOL SUMMARY x
1 KEY ROLES 13
1.1 Protocol Identification 13
1.2 Sponsor and Monitor Identification 13
1.3 Medical Officer 13
1.4 Site Investigators 13
1.5 Network Laboratory 14
1.6 Data Center 14
1.7 Study Operations 14
2 INTRODUCTION 15
2.1 Background of Microbicide Research 15
2.2 Description of Study Products 20
2.3 Tenofovir 1% Vaginal Gel (Tenofovir Gel) 20
2.4 2% Nonoxynol-9 Gel 21
2.5 Placebo Gel 21
2.6 *In Vitro* Studies 22
2.7 Condom Compatibility Studies 26
2.8 Animal Studies 27
2.9 Human Clinical Studies 38
2.10 Justification of No Treatment Arm 47
2.11 Study Hypothesis and Rationale 47
2.12 Justification of Dosing 49
3 OBJECTIVES 51
3.1 Primary Objective 51
3.2 Secondary Objectives 51
3.3 Exploratory Objectives 52
4 STUDY DESIGN 52
4.1 Identification of Study Design 52
4.2 Summary of Major Endpoints 52
4.3 Description of Study Population 53
4.4 Time to Complete Accrual 53
4.5 Study Groups 53
4.6 Expected Duration of Participation 53
4.7 Sites 53
5 STUDY POPULATION 54
5.1 Selection of Study Population 54

5.2 Inclusion Criteria 54
5.3 Exclusion Criteria 55
6 STUDY PRODUCT 57
6.1 Regimen 57
6.2 Administration 58
6.3 Study Product Formulation 58
6.4 Study Product Supply and Accountability 59
6.5 Assessment of Participant Adherence 61
6.6 Concomitant Medications 61
6.7 Prohibited Medications and Practices 61
7 STUDY PROCEDURES 62
7.1 Screening Visit 62
7.2 Enrollment/Baseline Evaluation Visit 64
7.3 Treatment 1 Visit 65
7.4 Follow-Up Phone Assessment 65
7.5 Treatment 2 Visit 66
7.6 Final Clinic Visit 66
7.7 Follow-up Phone Assessment Visit/Termination Visit 67
7.8 Follow-up Procedures for Participants Who Discontinue 68
Study Product 68
7.9 Interim Contacts and Visits 70
7.10 Final Contact 71
7.11 Clinical Evaluations and Procedures 71
7.12 Behavioral Measures 72
7.13 Laboratory Evaluations 73
7.14 Specimen Collection and Processing 74
7.15 Specimen Handling 74
7.16 Storage of Specimens for Future Use 74
7.17 Biohazard Containment 74
8 ASSESSMENT OF SAFETY 75
8.1 Safety Monitoring 75
8.2 Clinical Data Safety Review 75
8.3 Adverse Events Definitions and Reporting Requirements 76
8.4 Expedited Adverse Event (EAE) Reporting Requirements 78
8.5 Pregnancy and Pregnancy Outcomes 79
8.6 Social Harms Reporting 80
9 CLINICAL MANAGEMENT 80
9.1 Grading System 80
9.2 Dose Modification Instructions 80
9.3 Discontinuation of Study Product(s) in the Presence of Toxicity 81
9.4 General Criteria for Discontinuation of Study Product 81
9.5 Management of Specific Adverse Events 81
9.6 Criteria for Early Termination of Study Participation 82
10 STATISTICAL CONSIDERATIONS 82
10.1 Overview and Summary of Design 82
10.2 Study Endpoints 82

10.3 Accrual, Randomization, Blinding, and Sample Size 84
10.4 Data Analysis 85
11 DATA HANDLING AND RECORDKEEPING 89
11.1 Data Management Responsibilities 89
11.2 Source Documents and Access to Source Data/Documents 89
11.3 Quality Control and Quality Assurance 90
11.4 Study Coordination 90
12 CLINICAL SITE MONITORING 91
13 HUMAN SUBJECTS PROTECTIONS 91
13.1 Institutional Review Boards 91
13.2 Protocol Registration 92
13.3 Risk-Benefit Statement 93
13.4 Informed Consent Process 96
13.5 Participant Confidentiality 97
13.6 Special Populations 97
13.7 Compensation 98
13.8 Communicable Disease Reporting 98
13.9 Access to HIV-related Care 98
13.10 Study Discontinuation 99
14 PUBLICATION POLICY 99
15 APPENDICES 100
APPENDIX I: SCHEDULE OF STUDY VISITS AND EVALUATIONS 101
APPENDIX II: HIV ANTIBODY TESTING ALGORITHM 102
APPENDIX III: TOXICITY TABLES 103
APPENDIX IV: MANUAL FOR EXPEDITED REPORTING OF ADVERSE EVENTS TO DAIDS 104
APPENDIX V: HISTOPATHOLOGY SCORING SYSTEM 105
APPENDIX VI: SAMPLE INFORMED CONSENT FORM (SCREENING) 106
APPENDIX VII: SAMPLE INFORMED CONSENT DOCUMENT (ENROLLMENT) 113
APPENDIX VIII: SAMPLE INFORMED CONSENT DOCUMENT (Storage and Future Testing of Specimens) 124

TABLE OF FIGURES

Table 1: Summary of RAI in Surveys of Sexual Behavior 15
Table 2: Tenofovir Bioassay Data 28
Table 3: Rectal Irritation Study in New Zealand White Rabbits 30
Table 4: Use of Topical Tenofovir to Prevent Vaginal Transmission of SIV 33
Table 5: Other Studies of Tenofovir 1% Gel 41
Table 6: PrEP Studies 41
Table 7: Study Product Regimen 58
Table 8: Screening Visit 63
Table 9: Enrollment/Baseline Evaluation Visit (Day 0, Within 36 Days of Screening Visit) 64
Table 10: Treatment 1 Visit 65

Table 11: Follow-Up Phone Assessment 66
Table 12: Treatment 2 Visit 66
Table 13: Final Clinic Visit 67
Table 14: Follow-Up Phone Assessment Visit 68
Table 15: Early Termination Visit 69
Table 16: Interim Contacts and Visits 70
Table 17: Power Consideration for MTN-007 85
Table 18: Confidence Intervals 85

MTN-007

A Phase 1 Randomized, Double-Blinded, Placebo-Controlled Rectal Safety and Acceptability Study of Tenofovir 1% Gel

LIST OF ABBREVIATIONS AND ACRONYMS

3TC	lamivudine
ABC	abacavir
AE	adverse event
AIDS	Acquired Immunodeficiency Syndrome
ALT	alanine transaminase
APV	amprenavir
ARV	antiretroviral
AST	aspartate aminotransferase
ATP	adenosine triphosphate
AUC	area under the curve
C_{max}	maximum plasma concentration
CAB	community advisory board
CBC	complete blood count
CDC	Centers for Disease Control and Prevention
CFR	US Code of Federal Regulations
cGMP	current good manufacturing practices
CI	confidence interval
CRF	case report form
CRS	Clinical Research Site
CT	*Chlamydia trachomatis,* chlamydia
CTA	Clinical Trial Agreement
CVL	cervicovaginal lavage
DAIDS	Division of AIDS
ddC	zalcitabine
ddI	didanosine
DLV	delavirdine
DMPA	depot-medroxyprogesterone acetate
DNA PCR	deoxyribonucleic acid polymerase chain reaction
DSMB	Data and Safety Monitoring Board
EC_{50}	50% effective concentration
EAE	expedited adverse event
EIA	enzyme immunoassay
EFV	efavirenz
ET	Eastern Time
FDA	US Food and Drug Administration
FDR	false discovery rates
FTC	emtricitabine
g	gram
GC	*Neisseria gonorrhoeae,* gonorrhea

GCP	good clinical practice
GEE	generalized estimation equations
GLP	good laboratory practices
HBsAg	Hepatitis B surface antigen
HEC	hydroxyethyl cellulose
HIV	Human Immunodeficiency Virus
HPLC	high pressure liquid chromatography
HPTN	HIV Prevention Trials Network
hr	hour
HRA	high resolution anoscopy
HSV-2	Herpes simplex virus type 2
IATA	International Air Transport Association
IDV	indinavir
IFA	immunofluorescent antibody
IL	interleukin
IND	investigational new drug
IoR	Investigator of Record
IRB	Institutional Review Board
IV	intravenous
kg	kilogram
LDMS	Laboratory Data Management System
MDP	Microbicide Development Program
MIP	macrophage inflammatory protein
mL	milliliter
MMC	mucosal mononuclear cells
mRNA	messenger ribonucleic acid
MSM	men who have sex with men
MTN	Microbicide Trials Network
MTT	[1-(4,5-dimethylthiazol-2-yl)-3,5-diphenylformazan]
N-9	nonoxynol-9
NAAT	nucleic acid amplification testing
NE	neutrophil elastase
NFV	nelfinavir
ng	nanogram
NIAID	National Institute of Allergy and Infectious Disease
NIH	National Institutes of Health
NIMH	National Institute of Mental Health
NL	Network Laboratory
NNRTI	non-nucleoside reverse transcriptase inhibitor
NOAEL	no observed adverse effect level
NRTI	nucleoside reverse transcriptase inhibitor
NSAID	non-steroidal anti-inflammatory drugs
NVP	nevirapine
OHRP	Office for Human Research Protections
PBMC	Peripheral Blood Mononuclear Cell
PBS	phosphate buffered saline

PCR	polymerase chain reaction
PEP	post-exposure prophylaxis
PK	pharmacokinetics
PMPA	9-[(R)-2-(phosphonomethoxy) propyl] adenine monohydrate
PMPAp	PMPA monophosphate
PMPApp	PMPA diphosphate
PoR	Pharmacist of Record
PPD	Pharmaceutical Product Development
PrEP	pre-exposure prophylaxis
PRS	phone reporting system
PSRT	Protocol Safety Review Team
PSS	polystyrene sulfonate
PTID	Participant Identification Number
qc	quantitative competitive
qRT-PCR	quantitative real time reverse transcriptase polymerase chain reaction
RAI	receptive anal intercourse
RANTES	Regulated upon Activation-Normal T cell Expressed and Secreted
RMP	Rectal Microbicide Program
RNA	ribonucleic acid
RPR	rapid plasma reagin
RSC (DAIDS)	Regulatory Support Center
RT	reverse transcriptase
RT-PCR	reverse transcriptase polymerase chain reaction
RTV	ritonavir
SAE	serious adverse event
SCHARP	Statistical Center for HIV/AIDS Research & Prevention
SDF	stromal-derived factor
SDMC	Statistical and Data Management Center
SHIV	Simian/Human Immunodeficiency Virus
SIV	Simian Immunodeficiency Virus
SLPI	secretory leukocyte protease inhibitor
SMC	study monitoring committee
SOP	standard operating procedure
SQV	saquinavir
STI	sexually transmitted infection
T_{max}	time to peak concentration
$TCID_{50}$	50% Tissue Culture Infective Dose
TDF	tenofovir disoproxil fumarate (oral tenofovir)
TER	transepithelial resistance
TERIS	Teratogen Information System
TFV	tenofovir gel
Th	T helper
UCLA	University of California at Los Angeles
ULN	upper limit of normal

VI	virus isolation
VM	vaginal microbicide
vRNA	viral ribonucleic acid
WB	western blot
w/v	weight per volume
w/w	weight for weight
ZDV	zidovudine
µCi	microcurie
µg	microgram
µL	microliter
µM	micromole

MTN-007

A Phase 1 Randomized, Double-Blinded, Placebo-Controlled Rectal Safety and Acceptability Study of Tenofovir 1% Gel

PROTOCOL TEAM ROSTER

Philip Andrew, RN
Senior Research Clinician
FHI
PO Box 13950
Research Triangle Park, NC 27709 USA
Phone: 919-544-7040, Ext. 11213
Fax: 919-544-0207
Email: pandrew@fhi.org

Ratiya Pamela Kunjara Na Ayudhya, MT ASCP
Laboratory Manager
Microbicide Trials Network
204 Craft Avenue Room A540
Pittsburgh PA 15213 USA
Phone: 412-641-6393
Fax:412-641-6170
Email: pkunjara@mwri.magee.edu

Roberta Black, PhD
Microbicide Research Branch Chief
National Institutes of Allergy and Infectious Diseases (NIAID)/Division of AIDS (DAIDS)
6700 B Rockledge Drive, Room 5135
Bethesda, MD 20817 USA
Phone: 301-496-8199
Fax: 301-402-3684
Email: rblack@niaid.nih.gov

Katherine Bunge, MD
Protocol Safety Physician
Magee-Womens Hospital of UPMC
300 Halket Street
Pittsburgh, PA 15213 USA
Phone: 412-917-9936
Fax: 412-641-6170
Email: kbunge@mail.magee.edu

Marianne Morse Callahan
Deputy Director, Clinical
CONRAD
1911 North Fort Myer Drive, Suite 900
Arlington, VA 22209 USA
Phone: 703-276-3915
Fax: 703-524-4770
Email: mcallahan@conrad.org

Alex Carballo-Diéguez, PhD
Behavioral Research Working Group (BRWG) Representative
HIV Center for Clinical and Behavioral Studies
New York State Psychiatric Institute and Columbia University
1051 Riverside Drive, Unit 15
New York, NY 10032 USA
Phone: 212-543-5261
Fax: 212-543-6003
Email: ac72@columbia.edu

Missy Cianciola, MS
SDMC Senior Project Manager
Fred Hutchinson Cancer Research Center (FHCRC)-Statistical Center for HIV/AIDS Research and Prevention (SCHARP)
1100 Fairview Avenue North, LE-400
PO Box 19024
Seattle, WA 98109-1024 USA
Phone: 206-667-7290
Fax: 206-667-4812
Email: missy@scharp.org

Ross D. Cranston MD, FRCP
Site Investigator
Division of Infectious Disease
University of Pittsburgh Medical Center
Keystone Building, Suite 510
3520 Fifth Avenue
Pittsburgh, PA 15213 USA
Phone: 412-383-2054
Fax: 412-383-2900
Email: rdc27@pitt.edu

MTN-007

A Phase 1 Randomized, Double-Blinded, Placebo-Controlled Rectal Safety and Acceptability Study of Tenofovir 1% Gel

PROTOCOL TEAM ROSTER

James Dai, PhD
SDMC Biostatistician
FHCRC—SCHARP
1100 Fairview Avenue North, M2-C200,
PO Box 19024
Seattle, WA 98109-1024 USA
Phone: 206-667-7000
Fax: 206-667-4812
Email: jdai@fhcrc.org

Henry L. Gabelnick, PhD
Co-Sponsor Representative
CONRAD
1911 North Fort Myer Drive, Suite 900
Arlington, VA 22209 USA
Phone: 703-524-4744
Fax: 703-524-4770
Email: hgabelnick@conrad.org

Charlene S. Dezzutti, PhD
Biomedical Science Working Group Representative
Microbicide Trials Network
204 Craft Avenue
Pittsburgh, PA 15213 USA
Phone: 412-641-3462
Fax: 412-641-6170
Email: dezuttics@upmc.edu

Rebecca Giguere, MPH
BRWG Representative
HIV Center for Clinical and Behavioral Studies
New York State Psychiatric Institute and Columbia University
1051 Riverside Drive, Unit 15
New York, NY 10032 USA
Phone: 212-543-5829
Fax: 212-543-4385
Email: giguere@pi.cpmc.columbia.edu

Andrew D. Forsyth, PhD
Program Chief
Center for Mental Health Research on AIDS,
National Institute of Mental Health (NIMH)
6001 Executive Blvd, MSC 9619
Bethesda, MD 20892 USA
Phone: 301-443-8403
Fax: 301-443-9719
Email: aforsyth@mail.nih.gov

Yevgeny Grigoriev, MD, PhD
SDMC Clinical Affairs Safety Associate
FHCRC—SCHARP
1100 Fairview Avenue North, LE-400
PO Box 19024
Seattle, WA 98109-1024 USA
Phone: 206-667-3440
Fax: 206-667-4812
Email: ygrigori@scharp.org

David R. Friend, PhD
Director, Product Development
CONRAD
1911 N. Fort Myer Drive, Suite 900
Arlington, VA 22209 USA
Phone: 703-276-3906
Fax: 703-524-4770
Email: dfriend@conrad.org

Sharon Hillier, PhD
Principal Investigator
Microbicide Trials Network
204 Craft Avenue
Pittsburgh, PA 15213 USA
Phone: 412-641-8933
Fax: 412-641-6170
Email: shillier@mail.magee.edu

MTN-007

A Phase 1 Randomized, Double-Blinded, Placebo-Controlled Rectal Safety and Acceptability Study of Tenofovir 1% Gel

PROTOCOL TEAM ROSTER

Florian Hladik, MD, PhD
Associate Director, MTN Immunology Core
Department of Obstetrics and Gynecology
University of Washington
1959 NE Pacific Street, Box 356460
Seattle, WA 98195, USA
Phone: 206-221-2740/206-667-6836
Fax: 206-543-3915
Email : fhladik@fhcrc.org

Craig Hoesley, MD
Site Investigator
University of Alabama at Birmingham
1530 3rd Avenue South
BDB 467 Box 20
Birmingham, AL 35294 USA
Phone: 205-934-3365
Fax: 205-975-3232
Email: choesley@uab.edu

Marla Husnik, MS
SDMC Statistical Research Associate
FHCRC—SCHARP
1100 Fairview Avenue North, M2-C200
PO Box 19024
Seattle, WA 98109-1024 USA
Phone: 206-667-5633
Fax: 206-667-4812
Email: marla@scharp.org

Cindy Jacobson, PharmD
Director, Pharmacy Affairs
Microbicide Trials Network
204 Craft Avenue
Pittsburgh, PA 15213 USA
Phone: 412-641-8913
Fax: 412-641-6170
Email: cjacobson@mail.magee.edu

Sherri Johnson
Clinical Research Manager
FHI
4401 Wilson Boulevard, Suite 700
Arlington, VA 22203 USA
Phone: 703-516-9779 Ext.12127
Fax: 703-516-9781
Email: sjohnson@fhi.org

Jonathan Paul Lucas, MPH
Community Program Manager
FHI
Post Office Box 13950
Research Triangle Park, NC 27709 USA
Phone: 919-544-7040 Ext. 458
Fax: 919-544-0207
Email: jlucas@fhi.org

Kenneth Mayer, MD
Protocol Co-Chair
The Fenway Institute
Fenway Community Health
1340 Boylston Street
Boston, MA 02115 USA
Phone: 617-927-6400
Fax: 617-267-0764
Email: Kenneth_Mayer@brown.edu

James Maynard, MDiv
CWG Representative
The Fenway Institute
Fenway Community Health
1340 Boylston Street
Boston, MA 02215 USA
Phone: 617-927-6016
Fax: 617-267-0764
Email: jmaynard@fenwayhealth.org

MTN-007

A Phase 1 Randomized, Double-Blinded, Placebo-Controlled Rectal Safety and Acceptability Study of Tenofovir 1% Gel

PROTOCOL TEAM ROSTER

Ian McGowan, MD, PhD, FRCP
Protocol Chair
Microbicide Trials Network
204 Craft Avenue
Pittsburgh, PA 15213 USA
Phone: 412-641-8999
Fax: 412-641-6170
Email: imcgowan@pitt.edu

Jill Schwartz, MD
Medical Director
CONRAD
1911 Fort Myer Drive, Suite 900
Arlington, VA 22209 USA
Phone: 703-276-3913
Fax: 703-524-4770
Email: jschwartz@conrad.org

Jeanna Piper, MD
DAIDS Medical Officer
NIAID
Division of AIDS
6700 B Rockledge Drive, Room 5124
Bethesda, MD 20892 USA
Phone: 301-451-2778
Fax: 301-402-3684
Email: piperj@niaid.nih.gov

Mala Shah, MPH
Protocol Development Manager
Microbicide Trials Network
204 Craft Avenue
Pittsburgh, PA 15213 USA
Phone: 412-641-8999
Fax: 412-641-6170
Email: shahms@mail.magee.edu

Janet Schafer
Manager, Clinical Trials/ Regulatory Affairs
CONRAD
1911 Fort Myer Drive, Suite 900
Arlington, VA 22209 USA
Phone: 703-276-4034
Fax: 703-524-4770
Email: jschafer@conrad.org

Devika Singh MD, MPH
Protocol Safety Physician
Box 359927
Department of Global Health
International Clinical Research Center
325 Ninth Ave
Seattle WA 98104 USA
Phone: 206-744-8311
Fax: 206-520-3831
Email: dsingh@u.washington.edu

MTN-007

A Phase 1 Randomized, Double-Blinded, Placebo-Controlled Rectal Safety and Acceptability Study of Tenofovir 1% Gel

INVESTIGATOR SIGNATURE FORM

Version 2.0
August 13, 2010

A Study of the Microbicide Trials Network (MTN)

Funded by:
Division of AIDS, US National Institute of Allergy and Infectious Diseases
National Institute of Mental Health
US National Institutes of Health

IND Holder:
CONRAD

I, the Investigator of Record, agree to conduct this study in full accordance with the provisions of this protocol. I will comply with all requirements regarding the obligations of investigators as outlined in the Statement of Investigator (Form FDA 1572), which I have also signed. I agree to maintain all study documentation for at least two years following the date of marketing approval for the study product for the indication in which it was studied. If no marketing application is filed, or if the application is not approved, the records will be retained for two years after the investigation is discontinued and the US Food and Drug Administration is notified. Publication of the results of this study will be governed by MTN policies. Any presentation, abstract, or manuscript will be submitted to the MTN Manuscript Review Committee, DAIDS, and CONRAD Inc. for review prior to submission.

I have read and understand the information in the Investigator's Brochure(s), including the potential risks and side effects of the products under investigation, and will ensure that all associates, colleagues, and employees assisting in the conduct of the study are informed about the obligations incurred by their contribution to the study.

____
Name of Investigator of Record

______________________________ ______________________________
Signature of Investigator of Record Date

MTN-007

A Phase 1 Randomized, Double-Blinded, Placebo-Controlled Rectal Safety and Acceptability Study of Tenofovir 1% Gel

PROTOCOL SUMMARY

Short Title: **Tenofovir Rectal Safety Study**

Clinical Phase: 1

IND Sponsor: CONRAD

Protocol Chair: Ian McGowan, MD, PhD, FRCP

Protocol Co-Chair: Kenneth Mayer, MD

Sample Size: Approximately 60

Study Population: RAI (receptive anal intercourse)-abstinent, HIV-uninfected adults (male and female) from sites listed below

Participating Clinical Research Sites (CRS):

- Alabama Microbicides CRS, Birmingham, AL
- Fenway Clinic CRS, Boston, MA
- Pittsburgh CRS, Pittsburgh, PA

Study Design: Phase 1 randomized, double-blinded, multi-site, placebo-controlled trial

Study Duration: Participant accrual will take approximately 5 months and each participant will be on study for approximately 4 to 11 weeks. The total duration of the study will be approximately 8 months.

Study Products:

Rectal

- Tenofovir 1% gel
- 2% Nonoxynol-9 gel (Gynol-II®)
- Placebo gel (hydroxyethylcellulose-HEC)

Study Regimen:

After completing screening and baseline evaluation, eligible participants will be randomized to receive tenofovir 1% gel, 2% nonoxynol-9 gel (N-9) or placebo gel (15 per group). The study will also include a no treatment arm (15 participants). Participants will return to the clinic, where they will self-administer a single dose of the study gel under observation. Within approximately 30 minutes, lavage, stool, and rectal biopsy specimens will be obtained. After a one-week recovery period, participants will return to the clinic for assessment. If no significant adverse events (AEs) are reported they will begin to self-administer once-daily outpatient doses of the study gel for 7 days. Participants will return to clinic for evaluation and specimen collection after completion of 7 days of daily dosing.

Primary Objective:

- To evaluate the safety of tenofovir 1% gel when applied rectally

Primary Endpoint:

- Grade 2 or higher AEs as defined by the Division of AIDS Table for Grading the Severity of Adult and Pediatric Adverse Events, Version 1.0, December 2004 (Clarification dated August 2009) and/or Addenda 1 and 3 (Female Genital and Rectal Grading Tables for Use in Microbicide Studies) to this table

Secondary Objectives:

- To evaluate the acceptability of tenofovir 1% gel when applied rectally
- To evaluate the safety of the placebo gel when applied rectally
- To determine whether use of tenofovir 1% gel is associated with rectal mucosal damage
- To determine whether use of 2% nonoxynol-9 gel (Gynol-II®) is associated with rectal mucosal damage

Secondary Endpoints:

- The proportion of participants who at their Final Clinic Visit report via the acceptability questionnaire that they would be very likely to use the candidate microbicide during receptive anal intercourse
- Grade 2 or higher adverse events in the placebo gel arm, as defined by the Division of AIDS Table for Grading the Severity of Adult and Pediatric Adverse Events, Version 1.0, December 2004 (Clarification dated August

2009) and/or Addenda 1 and 3 (Female Genital and Rectal Grading Tables for Use in Microbicide Studies)

- Changes in the following parameters:
  - Epithelial sloughing
  - Intestinal histopathology
  - Intestinal mucosal mononuclear cell phenotype
  - Intestinal mucosal cytokine messenger RNA (mRNA)
  - Intestinal mucosal gene expression arrays
  - Cytokine profile in rectal secretions
  - Fecal calprotectin
  - Microflora

Exploratory Objectives:

- To determine whether regional heterogeneity exists between mucosal endpoints in samples collected at 9 cm and 15 cm for all parameters examined

- To determine whether there is a correlation between histological abnormality and changes in mucosal biomarkers

Exploratory Endpoints:

- Changes in the following parameters:
  - Epithelial sloughing
  - Intestinal histopathology
  - Intestinal mucosal mononuclear cell phenotype
  - Intestinal mucosal cytokine messenger RNA (mRNA)
  - Intestinal mucosal gene expression arrays
  - Cytokine profile in rectal secretions
  - Fecal calprotectin
  - Microflora

1 KEY ROLES

1.1 Protocol Identification

Protocol Title:	A Phase 1 Randomized, Double-Blinded, Placebo-Controlled Rectal Safety and Acceptability Study of Tenofovir 1% Gel
MTN Protocol Number:	MTN-007
Short Title:	Tenofovir Rectal Safety Study
Date:	August 13, 2010

1.2 Sponsor and Monitor Identification

Funding Agency:	Division of AIDS (DAIDS)/National Institute of Allergy and Infectious Diseases (NIAID)/National Institute of Mental Health (NIMH)/National Institutes of Health (NIH)<br>6700 B Rockledge Drive<br>Bethesda, MD 20892 USA
IND Holder:	CONRAD<br>1911 North Fort Myer Drive, Suite 900<br>Arlington, VA 22209 USA
Monitor:	Pharmaceutical Product Development (PPD), Inc.<br>929 North Front Street<br>Wilmington, NC 28401-3331 USA

1.3 Medical Officer

Jeanna Piper, MD
National Institute of Allergy and Infectious Diseases
Division of AIDS
6700 B Rockledge Drive, Room 5124
Bethesda, MD 20892 USA

1.4 Site Investigators

Craig Hoesley, MD
Kenneth Mayer, MD
Ross Cranston, MD, FRCP

1.5 Network Laboratory

MTN Network Laboratory
Magee-Womens Research Institute
204 Craft Avenue, Room A540
Pittsburgh, PA 15213 USA

1.6 Data Center

SCHARP
Fred Hutchinson Cancer Research Center
1100 Fairview Avenue N., LE-400
PO Box 19024
Seattle, WA 98109-1024 USA

1.7 Study Operations

FHI
PO Box 13950
Research Triangle Park, NC 27709 USA

2 INTRODUCTION

2.1 Background of Microbicide Research

To date, the majority of microbicide research has focused on the assessment of the safety and effectiveness of vaginal microbicides used for the prevention of HIV transmission via the vaginal compartment. Receptive anal intercourse (RAI) is common among men who have sex with men (MSM) and there is increasing evidence that heterosexual women in the developed and developing world also practice anal sex (Table 1). It can therefore be anticipated that once vaginal microbicides are licensed, they will be used in both the vaginal and rectal compartments. As a consequence, there is a need to evaluate both the rectal and vaginal safety profiles of candidate microbicides.

Table 1: Summary of RAI in Surveys of Sexual Behavior

Population	Men, Women, or Men and Women	N	Prevalence of RAI	Reference
MSM in EXPLORE study	Men	4,295	48-54%	Koblin et al. 2003[1]
High risk women	Women	1,268	32%	Gross M et al. 2000[2]
College students	Men and women	210	20%	Civic D 2000[3]
US Survey (15-44 year olds)	Men and women	12,571	35-40%	Mosher 2005[4]
Californian residents	Men and women	3,545	6-8%	Erickson PI et al. 1995[5]

The Rectal Microbicide Program (RMP-02)/MTN-006 and MTN-007 clinical protocols have therefore been developed to assess the safety and pharmacology of tenofovir gel when used rectally in men and women as well as address critical questions in rectal microbicides through explorative objectives. Although a single combined study was considered, the complexity of the study and the potential participant burden were considered to be substantial and as a result the protocol teams felt compartmentalization of key activities into two concurrent and mutually supportive clinical studies was a more efficient, less burdensome and safer approach. RMP-02/MTN-006 concentrated on systemic, mucosal and tissue pharmacology and pharmacodynamics of tenofovir 1% gel following administration, including *ex vivo* challenge of biopsies with HIV as a potential marker for retained tissue anti-HIV activity. MTN-007 will focus on accumulating Phase I safety and acceptability data, while providing in its secondary and exploratory objectives the data needed to determine maximum and minimum parameters for a suite of potential rectal safety assays using comparisons of the results from the Gynol II® (2% N-9), placebo, and no treatment arms. In order to assure comparability both protocols have been operationally (general schema, procedures, inclusion and exclusion criteria, etc.) harmonized. The trials also have in common the basic suite of rectal safety assays, which have been developed by

the University of California at Los Angeles (UCLA) Microbicide Development Program (MDP) team. Together these two trials should act synergistically to determine/establish: (1) initial safety of tenofovir 1% gel as a rectal microbicide; (2) acceptability of a vaginally formulated tenofovir gel when used rectally; (3) pharmacokinetics (PK)/ pharmacodynamic (PD) parameters for rectal tenofovir 1% gel use; and (4) further validate the proposed rectal safety suite by establishing maximum (Gynol II®), minimum (placebo gel), and baseline (no treatment arm) mucosal responses, while proactively managing participant and clinical site burden. Additionally, the co-sponsorship of the RMP-02/MTN-006 trial (UCLA and University of Pittsburgh sites) will support the transition of the UCLA site–developed safety suite to the MTN Network Laboratory (NL) (RMP-02/MTN-006), which will then disseminate the suite to the MTN-007 sites.

Vulnerability of the Rectal Compartment to HIV Infection

The rectal compartment is highly vulnerable to HIV transmission. A single layer of columnar epithelium separates the intestinal lumen from the lamina propria. The lamina propria is populated with a broad range of HIV target cells including macrophages, dendritic cells, and activated CD4+ T lymphocytes expressing the CCR5 and CXCR4 HIV-1 coreceptors.[6] It is likely that the immune composition of the rectal mucosa is at least partially responsible for the 10- to 20-fold increased risk of HIV transmission associated with anal[7, 8] compared to vaginal intercourse.[9, 10] Any product that induces local inflammation is likely to further increase this risk by recruiting and/or activating the immune target cells.

Developing Safety Standards for Rectally-Administered Microbicides

Methods to assess microbicide induced toxicity in the rectal compartment are in a state of evolution. Mucosal changes may be subtle and require new modes of detection (polymerase chain reaction (PCR), flow cytometry, immunohistochemistry, etc). For this reason, the HIV Prevention Trial Network (HPTN) sponsored the HPTN 056 study "Characterization of Baseline Mucosal Indices of Injury and Inflammation in Men For Use in Rectal Microbicide Trials" conducted at UCLA.[11] The lessons learned from the HPTN 056 trial have guided the selection of parameters to be included in this protocol. As these are assays in development and clinical relevance remains to be defined, these will not be safety indices but exploratory endpoints. The rationale for selecting each of these endpoints is further described below:

Epithelial sloughing

Rectal lavage and examination of effluent for shedding of epithelial cells has been used to characterize the rectal safety profile of microbicide candidates in murine, non-human primate, and human studies. This approach has demonstrated that N-9 is associated with transient epithelial disruption. Substantial reversal of these mucosal changes occurred by 2 hours and microscopically normal epithelium was noted after 24 hours.[12-14] In contrast, administration of VivaGel®, C31G, Carraguard, or UC781 to non-human primates did not result in epithelial desquamation.[15-18] Since the epithelial sloughing does not have an absolute, quantifiable threshold, the scoring system of 0-to-4 will be used.[16] Each of four petri-dish quadrants is scored as either 0 or 1, indicating either the

absence or presence of epithelial sheets. The total score for each preparation can therefore be from 0-4. Changes pre- and post-treatment will be analysed.

Intestinal histopathology

Histopathological assessment of intestinal tissue is a routine method of demonstrating mucosal abnormality associated with gastrointestinal diseases such as ulcerative colitis, Crohn's disease, and gluten enteropathy (celiac disease). In general, mucosal change in these diseases can be quite dramatic whereas microbicide-induced changes may be quite subtle. As a consequence we will use a qualitative scoring system (See Appendix V) developed by the inflammatory bowel disease community[19] and adapted for use in HPTN 056.[11] Prior to the HPTN 056 study, one rectal microbicide study using histological data,[14] employed a simple scoring system of normal, slightly abnormal, or abnormal. Using this histological system, 69% of the placebo recipients and 89% of the N-9 recipients had slightly abnormal or abnormal rectal biopsies. The scoring system developed for the HPTN 056 study might provide better discrimination between abnormal and normal histology.

Intestinal mucosal mononuclear cell phenotype

Enzymatic digestion of intestinal biopsies and flow cytometric analysis of T cell populations[20] will be used to determine if product administration is associated with changes in mucosal T cell populations, co-receptor expression, or T cell activation. Co-receptor expression (e.g., CCR5, CXCR4, etc.) on mucosal T cells is important for HIV-1 entry. In healthy HIV-1 seronegative individuals, the expression level of CCR5 is increased seven-fold in mucosal mononuclear cells (MMC) compared to peripheral blood mononuclear cells (PBMC).[6] CXCR4, however, is expressed in CD45RO+ T cells in similar levels as in MMC and PBMC. It was recently shown that MMC are more easily infected with HIV-1 than PBMC.[21, 22] Explanations for the high susceptibility of MMC to HIV-1 may include the increased expression of HIV-1 co-receptors, especially CCR5, as well as the heightened activation status of the MMC. The expression of CCR5 has been shown to be up-regulated by pro-inflammatory and T helper (Th)-1 cytokines, while Th-2 cytokines up-regulate CXCR4.[23, 24] This suggests that expression of CCR5 and CXCR4 is partly controlled by Th1/Th2 type of cytokines, which have been shown to be up-regulated in rectal mucosa from HIV-infected patients.[11] It will be important to ascertain whether microbicidal agents trigger similar responses and associated increased vulnerability to HIV infection.

All flow cytometry will be performed at the MTN Core Laboratories in Pittsburgh, PA. This approach has been used before by other investigators[25, 26] and it is anticipated that it will be possible to conduct adequate assessment of mucosal T cell populations on these samples.

Intestinal mucosal cytokine mRNA

Documentation of an increase in mucosal production of pro-inflammatory cytokines such as interleukin (IL)-6 or IL-8 following microbicide exposure may act as a surrogate marker of product induced toxicity.[27] Recent work has helped define the optimal methodology to measure cytokines in biological samples.[28] In MTN-007 we will

measure proinflammatory cytokines that have been associated with increased recruitment of potential HIV target cells and/or replication of HIV infection. Previous HIV mucosal pathogenesis studies have demonstrated significant increases in mucosal cytokine mRNA in individuals with untreated HIV infection compared to controls or patients with undetectable plasma HIV viremia.[29, 30]

CCL5, also known as Regulated upon Activation-Normal T Cell Expressed and Secreted (RANTES), macrophage inflammatory protein (MIP)-1α and MIP-1β13 are the natural ligands for CCR5 while stromal-derived factor (SDF) -1 is the ligand for CXCR4. The physiological function of β-chemokines and their receptors is to direct migration of recruited lymphocyte subsets to sites of inflammation and immune activation furthering the inflammatory cascade. Blocking chemokine activity has proved to be effective for inhibiting the migration of certain leukocytes while up-regulation of chemokine receptors and their ligands are characteristic correlates of mucosal inflammation.[31, 32] Immune activation of resting $CD4^+$ T cells has been shown to trigger viral replication and spread.[33, 34]

In MTN-007 we will use quantitative, real-time reverse transcriptase polymerase chain reaction (qRT-PCR) to quantify mucosal mRNA expression of the following proinflammatory cytokines, chemokines, and chemokine receptors: IL-1β, IFN-γ, TNF-α, IL-6, IL-8, IL-12, IL-17, IL-23, MIP-1α, MIP-1β, RANTES, and CCR5.

Cytokine profile in rectal secretions

As discussed above, measurement of cytokines or chemokines in mucosal tissue or local secretions may provide important information regarding the potential for a candidate microbicide to induce mucosal toxicity. In addition to the mRNA analysis of intestinal tissue biopsies, we will quantify cytokine levels in rectal secretions using the Luminex® technique, which can measure multiple cytokines or chemokines in small volumes (< 100 μL) of rectal secretions. We will use Luminex® to measure the following cytokines, chemokines, and chemokine receptors: IL-1β, IFN-γ, TNF-α, IL-6, IL-8, IL-12, IL-17, IL-23, MIP-1α, MIP-1β, RANTES, and CCR5.

Intestinal mucosal gene expression arrays

Currently, no validated biomarkers that reliably measure the genital toxicity of microbicides are available. Certain cytokines and chemokines may in principle be suitable as mucosal biomarkers for microbicide-induced toxicity or inflammation. The concentration of inflammatory cytokines in mucosal secretions has therefore been evaluated in prior microbicide studies. However, these studies have their limitations. First, only a few genes/proteins can be studied from one sample. Second, it is unclear what constitutes a meaningful change in cytokine concentration.

Because of the described limitations when measuring individual biomarkers, it would be useful to develop assays that evaluate the cumulative impact of candidate microbicides on mucosal immune function as a whole. To evaluate the global impact of microbicides on the mucosa, we will perform gene expression microarrays on mRNA isolated from mucosal biopsies taken before and after application of tenofovir, N-9 or placebo gel.

HumanWG-6 Expression BeadChips (Illumina Inc., San Diego, CA) permit the measurement of 48,000 mRNAs simultaneously on a single high-density oligonucleotide microarray. This offers the opportunity to: (1) identify signature expression patterns of dozens or even hundreds of genes that correlate with microbicide side effects on the mucosa; (2) interpret expression changes of a particular gene group, such as inflammatory cytokines, in relationship to other genes; and (3) cross-validate the array results with measurements of mucosal cytokine mRNAs by RT-PCR and of cytokine proteins in mucosal secretions by the Luminex® technique (both performed in other participating laboratories).

An expected outcome of the array studies is the identification of groups of genes, in particular apoptotic, pro-inflammatory and/or innate immunity-related genes, which are modulated significantly from baseline in response to topical N-9 application. In comparison, these changes are expected not to occur after tenofovir or placebo gel application. Another expected outcome of the array studies is the validation of any significant cytokine/chemokine/chemokine receptor changes found by qRT-PCR and Luminex® measurements. Similar results in all three assays (expression array, qRT-PCR and Luminex®) will underscore the potential usefulness of a biomarker to predict toxicity of a candidate microbicide. Moreover, interpreting such a promising marker in the light of other gene expression changes in the microarray will provide an opportunity to better understand its biological significance.

<u>Fecal calprotectin</u>
Stool samples will be collected at the time of rectal lavage for the measurement of fecal calprotectin. Calprotectin accounts for 60% of the cytoplasmic protein fraction of polymorphonuclear granulocytes and is also found in monocytes, macrophages, and eosinophils.[35, 36] Calprotectin plays an important role in innate immunity and has antibacterial, antifungal, and immunomodulatory effects *in vivo*. Because intestinal granulocytes end their lifespan by migrating through the intestinal wall and since granulocyte-derived calprotectin can be found in feces, calprotectin is felt to be a useful indirect index of mucosal inflammation.[37, 38] In fact, fecal calprotectin levels are elevated in inflammatory bowel disease[39, 40] and correlate well with disease activity in Crohn's disease and ulcerative colitis. In addition, fecal calprotectin levels have been found to be significantly elevated in first-degree relatives of patients with Crohn's disease even though all the relatives were clinically asymptomatic.[41] These data suggest that the fecal calprotectin assay may be sufficiently sensitive to respond to subtle increases in mucosal inflammation. Fecal calprotectin has a sensitivity of 96% in discriminating between healthy controls (2mg/L; 95% CI 2-3 mg/L) and subjects with active inflammatory bowel disease (91 mg/L; 95% CI 59-105 mg/L).[40]

<u>Microflora</u>
Assessment of pre- and post-exposure changes in rectal microflora will be conducted. It is currently unknown whether rectal administration of tenofovir 1% gel will prompt a change in the rectal microflora. Transient reductions in vaginal lactobacilli have been noted with the administration of candidate microbicides.[16] There are no rectal microflora data from human microbicides although non-human primate studies have not

demonstrated significant changes in rectal microflora following rectal administration of vaginal microbicides.

Assessing Acceptability of Rectally-Administered Microbicides
Prevention tools are effective only if used. The limited use of condoms by many at-risk individuals illustrates the importance of a product's acceptability and perceived need, i.e., the willingness of the users of the product to use it correctly and consistently. This study will explore the acceptability of tenofovir 1% gel for rectal use by means of a behavioral assessment that includes both structured and semi-structured methods. This assessment will evaluate not only product acceptability, but also the acceptability of a vaginal applicator for rectal product application. The behavioral assessment consists of two elements: 1) a Baseline Behavioral Questionnaire and 2) a Product Acceptability Questionnaire. The questionnaires were originally developed based on in-depth qualitative interviews of 20 participants in the first phase of R01 HD046060 "Topical Microbicide Acceptability," (Carballo-Diéguez, PI), a study that focused on acceptability of rectal microbicides among men and women.[42, 43]

The questionnaires were subsequently administered to more than 100 MSM in Boston and New York, and to 36 men and women participating in a rectal microbicide study in Los Angeles (Peter Anton, PI). This study showed that there were no comprehension problems or other difficulties. There are additional advantages to using the same product-acceptability instrument across studies, since this allows more valid post-hoc comparisons across studies.

2.2 Description of Study Products

2.3 Tenofovir 1% Vaginal Gel (Tenofovir Gel)

Tenofovir (sometimes referred to as PMPA,9-[(R)-2-(phosphonomethoxy)propyl] adenine monohydrate) is a novel nucleotide analogue belonging to the class of acyclic phosphonomethylether nucleotides with potent activity against retroviruses.[44] Further information is available in the current version of the tenofovir gel investigator's brochure.[44]

2.3.1 Mechanism of Action

Tenofovir is an acyclic nucleotide analogue of adenosine monophosphate. Once inside the cell tenofovir is phosphorylated by cellular enzymes to form tenofovir diphosphate. Tenofovir diphosphate is a competitive inhibitor of HIV-1 RT that terminates the growing DNA chain. Tenofovir diphosphate is a weak inhibitor of mammalian DNA polymerases α, β, and mitochondrial DNA polymerase γ.

2.3.2 Strength of Study Product

The strength of the tenofovir gel will be the concentration (1%) previously tested in HPTN 050 (investigational new drug (IND 55,690)), CAPRISA-004, CONRAD A04-095

(IND 73,382) and A04-099 (IND 73,382), HPTN 059 (IND 55,690), MTN-001 (IND 55,690), MTN-002 (IND 55,690), and RMP-02/MTN-006 (CONRAD IND 73,382). From the current good manufacturing practice (cGMP) formulators (DPT Pharmaceuticals), the density of the gel is 1.06 g/mL which has been rounded up to 1.1 g/mL of gel. Each gram of gel contains 10 mg of tenofovir, resulting in a total of 44 mg of tenofovir delivered in each application (or 0.044 grams of tenofovir).

2.4 2% Nonoxynol-9 Gel

N-9 is a non-ionic surfactant that has been most commonly used as a spermicidal agent in several over-the-counter products including condoms, gels, and films.

2.4.1 Mechanism of Action

N-9 is a non-ionic surfactant that destroys the integrity of the lipid bilayer membrane with a virucidal action that disrupts the viral envelope. It is by this mechanism that N-9 has its effect on HIV-1.[45, 46]

2.4.2 Strength of Study Product

The strength of the study product will be 2%. This strength has been used in previous human studies of this compound and is one of the strengths currently marketed as a spermicidal gel in North America. Murine (2% to 3.5% N-9)[13], nonhuman primate (4% N-9)[47] and human (2% N-9)[12] rectal studies demonstrated acute transient epithelial disruption and sloughing at these doses. In a study by Tabet et al.[14], in which participants received a 3.5% formulation of N-9 in escalating doses over a 6-week period, minor histological abnormalities were commonly observed.

2.5 Placebo Gel

The placebo gel is the hydroxyethylcellulose (HEC)-based or “Universal” placebo gel,[48] a vaginal product that contains HEC as the thickener, purified water, sodium chloride, sorbic acid and sodium hydroxide. The placebo gel is used to approximate the viscosity of other microbicide gel candidates.

2.5.1 Mechanism of Action

The placebo gel is designed to be inactive in the vagina. The gel is isotonic and formulated at a pH of 4.4 to avoid disrupting the normal vaginal pH. It is formulated with minimal buffering capacity to avoid the inactivation of sexually transmitted pathogens.

2.5.2 Strength of Study Product

Placebo gel at a concentration of 2.7% w/w HEC will be used in this study.

2.6 *In Vitro* Studies

2.6.1 Reformulated Tenofovir Gel

The reformulated tenofovir 1% gel being used in MTN-007 has a lower osmolality than the original formulation that was used in prior studies.[49] Additionally, the density of the reformulated tenofovir 1% gel is 1.02 g/mL. This will be the first clinical trial using this formulation of tenofovir 1% gel. Safety testing in epithelial cell lines has demonstrated retention of transepithelial resistance (TER) by Caco-2 and HEC-1-A cell lines. Previous results showed the original formulation to induce a transient drop in the epithelial resistance. This was not observed with the reformulated tenofovir gel. Safety testing of colorectal explants shows similar MTT (Formazan [1-(4, 5-dimethylthiazol-2-yl)-3, 5-diphenylformazan]) results with both formulations. However, histological testing showed retention of the epithelium after application of the reformulated tenofovir gel as compared to epithelial stripping with the original formulation. Additional testing in colorectal explant cultures also showed that the new formulation did not compromise product efficacy. Collectively, these data suggest that the reformulated tenofovir gel is just as effective as the original formulation but is less toxic to the epithelium.

2.6.2 Tenofovir

Formulation Testing

The physiological properties evaluated included osmolarity, viscosity, pH, and *in vitro* release.[50] In comparison to isosmolar standards, tenofovir 1% gel and its matched placebo exhibited 11.5-fold and 11-fold, greater osmolarity, respectively than isosmolar conditions implying that this formulation is hyperosmolar. Both gels had a pH of ~4.4, which is similar to the vaginal environment. Viscosity evaluations were conducted for both tenofovir 1% gel and its matched placebo gel. The viscosity of the tenofovir 1% gel and its placebo at 30 rpm showed reproducible results in 3 trials. Both gels were found to be shear thinning in nature. Thinning viscosity indicates that it is “flowable” which allows for even spread across mucosal surfaces.

Safety Testing in Cell Lines

Tenofovir 1% gel and its placebo gel were evaluated for its effect on the viability of colorectal Caco-2 epithelial cell line.[50] Viability of the Caco-2 epithelial cell line after a 24-hour exposure to tenofovir 1% gel or placebo gel showed minimal reduction; a 1:10 dilution of both gels yielded ≥ 60% viability. To put this into perspective, the over-the-counter preparations of N-9 (3%) and KY® jelly (Johnson & Johnson, New Brunswick, NJ) need to be diluted a minimum of 1:1000 and 1:100, respectively, of their original formulation to yield ≥ 60% viable epithelial cultures.[47] Using the 1:10 dilution, a “2 hours per day for 5 days” exposure experiment was performed to evaluate the impact of extended use on Caco-2 cell viability. No reduction in Caco-2 viability was noted after the 5-day exposure, indicating that the 1:10 dilutions of both gels were stable concentrations for use in further analysis.

The ability of mucosal epithelial cells to maintain an intact, polarized monolayer in the presence of a microbicide is a possible predictor of that product's safety on colorectal tissues because the epithelial layer is integral in the protection against sexually transmitted infections including HIV. Therefore, Caco-2 cells were plated in duplicate in transwell plates, and their TER was measured using the Millicell® ERS meter (Millipore, Billerica, MA) to form a polarized monolayer. When the cells reached plateau TER, a 1:10 dilution of tenofovir 1% gel or placebo gel and a 1:50 dilution of N-9 were added to the apical side of the transwells. The TER was measured over a 24-hour period. Tenofovir 1% gel and placebo gel maximally reduced the TER as compared to the control (68% and 59% respectively) after 4 hours. Over the next 20 hours, the TER returned to control TER levels. N-9-treated wells, however, continually declined and reached background levels after 4 hours. These data suggest that hyperosmolar nature of the tenofovir 1% gel formulation resulted in the transient loss of the epithelial monolayer resistance.

Safety Testing in Colorectal Explant Cultures

Tenofovir 1% gel and its matched placebo were tested for toxicity to colorectal explant cultures.[50] Briefly, duplicate polarized tissues were exposed to product for 18 hours and then washed to remove excess product. One of the duplicate tissues was incubated with MTT to measure the reduction to formazan and the other was placed in 10% buffered formalin for histology. Up to 5 different tissue donors were used. Tenofovir 1% gel and the placebo did not reduce the viability of the colorectal explants as based on the MTT assay. When assessed for histologic changes, the tenofovir 1% gel and to a lesser extent the placebo treated tissues showed fractured epithelium with an intact lamina propria. This result may be due to the hyperosmolar formulation of the tenofovir 1% gel and this data would correspond to the changes noted for the epithelial cell line TER.

Efficacy Testing in Colorectal Explant Cultures

The efficacy of the tenofovir 1% gel and placebo gels were assessed using the polarized colorectal explant culture system.[50, 51] The explants were set-up in duplicate and exposed to HIV-1 without or with 1:5 dilutions of tenofovir 1% or placebo gels on the apical side. The explants were allowed to culture overnight and then washed. The explants were followed for 21 days and HIV-1 replication was assessed by the production of p24 in the basolateral supernatant. The tenofovir 1% gel was effective at preventing HIV-1 infection of the tissue. The placebo was also partially effective at reducing the HIV-1 infection. This has been noted previously for other products that were evaluated.[51]

Anti-HIV-1 Activity

The *in vitro* antiviral activity of unformulated tenofovir against laboratory and clinical isolates of HIV-1 was assessed in lymphoblastoid cell lines, primary monocyte/macrophage cells and peripheral blood lymphocytes.[52, 53] The 50% effective concentration (EC_{50}) values for tenofovir were in the range of 0.04 µM - 8.5 µM. In drug combination studies of tenofovir with NRTIs (abacavir [ABC], didanosine [ddI], lamivudine [3TC], stavudine [d4T], zalcitabine [ddC], zidovudine [ZDV]); non-nucleoside

reverse transcriptase inhibitors (NNRTI) (delavirdine [DLV], efavirenz [EFV], nevirapine [NVP]); and protease inhibitors (amprenavir [APV], indinavir [IDV], nelfinavir [NFV], ritonavir [RTV], saquinavir [SQV]), additive/synergistic effects were observed. Tenofovir displayed antiviral activity *in vitro* against HIV-1 clades A, B, C, D, E, F, G, and O (EC_{50} values 0.5 µM - 2.2 µM) and showed strain specific activity against HIV-2 (EC_{50} values ranged from 1.6 µM to 5.5 µM).

Resistance

HIV-1 isolates with reduced susceptibility to unformulated tenofovir have been selected *in vitro.*[53, 54] These viruses expressed a K65R mutation in RT and showed a 2-4 fold reduction in susceptibility to tenofovir. Of note, this mutation also confers increased susceptibility to some other nucleoside reverse transcriptase inhibitors (NRTI), and is associated with approximately 50% reduction in the replicative capacity of HIV-1 (potentially resulting in a “less fit” virus).[55] Tenofovir-resistant isolates of HIV-1 have been recovered from some patients treated with Viread® in combination with certain antiretroviral (ARV) agents.[53] In treatment-naïve patients, 8/47 (17%) isolates from patients failing Viread® + 3TC + EFV through week 144 showed >1.4 fold (median 3.7) reduced susceptibility *in vitro* to tenofovir.

Cross-resistance

Cross-resistance among certain NRTIs has been recognized.[52, 53] The M184V/I and/or K65R substitutions selected *in vitro* by the combination of emtricitabine (FTC) and unformulated tenofovir are also observed in some HIV-1 isolates from subjects failing treatment with tenofovir in combination with either 3TC or FTC, and either abacavir, didanosine, or zalcitabine. Therefore, cross-resistance among these drugs may occur in patients whose virus harbors either or both of these amino acid substitutions. In treatment-experienced patients, 14/304 (5%) isolates from patients failing Viread® through week 96 showed >1.4 fold (median 2.7) reduced susceptibility to tenofovir. Genotypic analysis of resistant isolates showed a mutation in the HIV-1 RT gene resulting in the K65R amino acid substitution. HIV-1 isolates from patients (n = 20) whose HIV-1 expressed a mean of 3 ZDV-associated RT amino acid substitutions (M41L, D67N, K70R, L210W, T215Y/F, or K219Q/E/N) showed a 3.1-fold decrease in the susceptibility to tenofovir. Multinucleoside resistant HIV-1 with a T69S double insertion mutation in the RT showed reduced susceptibility to tenofovir.[54]

2.6.3 2% Nonoxynol-9 Gel

Formulation Testing

None has been done. The 2% N-9 gel was purchased in a local pharmacy.

Safety Testing in Cell Lines

Colorectal epithelial cell lines, Caco-2 and SW837, were exposed to 10-fold dilutions of the gel for 24 hours.[56] The dilution that resulted in greater than 60% viability was 1:1000 which equates to 20 µg/mL of N-9 (20 mg/mL in original gel). Using this dilution of 2% N-9 gel, no significant impact in the Caco-2 transepithelial resistance was noted. However, if the dose was 2 mg/mL (1:10 dilution of the original gel), the monolayer

resistance was completely destroyed by 2 hours after application. These data suggest that significant dilution of the N-9 gel is required to not be toxic to the colorectal epithelial cell lines.

Safety Testing in Colorectal Explant Cultures

Colorectal explant tissue was set-up in duplicate in a polarized transwell system. Two percent N-9 gel diluted to 2 mg/mL or placebo gel was applied to the apical side of the tissue and allowed to culture overnight.[51] After culture, the tissues were washed and one of the duplicates was placed in formalin for histological analysis and the other was placed in 1-(4,5-dimethylthiazol-2-yl)-3,5-diphenylformazan (MTT) to determine viability (adenosine triphosphate (ATP) activity). Complete necrosis of the epithelium and lamina propria was observed in N-9-treated explants. Explants treated with each placebo were histologically normal. Exposure to N-9 produced the greatest reduction in viability, to 47% of that of the control (P<.052). Explants treated with the placebo gel were not significantly different from the control. Collectively, these data show that 2% N-9 gel was very toxic to the colorectal explant tissues.

Anti-HIV-1 Activity

To assess the anti-HIV-1 activity, 2% N-9 gel was diluted 1:1000 to 20 µg/mL and applied to peripheral blood mononuclear cells (PBMCs) with HIV-1 isolates that included laboratory isolates (HIV-1_{BaL} and HIV-1_{LAI}) and 3 primary isolates (one each of subtype A, C, and CRF01-AE).[56] HIV-1 replication was detected using an HIV-1 p24gag ELISA from collected culture supernatants. The diluted N-9 completely inhibited HIV-1_{BaL}, subtype A, and subtype CRF01-AE infection but had 1 $\log_{10}$ reduction against HIV-1_{LAI} or subtype C (58% and 88% inhibition, respectively). Moreover, the placebo gel was equally as effective against HIV-1_{BaL} as the diluted 2% N-9 gel but was not effective against the other subtypes.

Colorectal explant tissue was set-up in a polarized transwell system and 2% N-9 gel diluted to 2 mg/mL or placebo gel were mixed with HIV-1_{BaL} and applied to the apical side of the tissue and allowed to culture overnight.[51] After culture, the tissues were washed and HIV-1 replication was monitored by the HIV-1 p24gag ELISA in the basolateral supernatant of the explant cultures. No HIV-1_{BaL} was recovered from the N-9-treated tissue due to the lack of viable immune cells to support viral replication. The placebo gel had minimal impact on HIV-1 infection of the tissue.

2.6.4 Placebo Gel

Formulation Testing

Analyses of pH (placebo gel mixed with human seminal plasma, 8.03 ± 0.26) found that the gel formulation did not show significant buffering capacity and could not acidify the alkaline pH of seminal plasma, a favorable property for a placebo formulation.[57] *In vitro* assessments of spermicidal activity utilizing human semen from healthy donors showed that the placebo gel had no significant deleterious effects on sperm motility, even after 60-minute incubation.

Safety Testing in Cell Lines
Dilutions of the HEC gel in culture medium exhibited negligible toxicity to human vaginal epithelial cells (standard MTT assay), even at the lowest dilution tested (1:2).[48] Exposure of human vaginal epithelial cells to the HEC gel resulted in minimal IL-1α induction, even at the lowest dilutions tested (lowest dilution, 1:2). Additional studies have shown that HEC gel is safe to peripheral blood mononuclear cells, and colorectal epithelial cell lines.[51, 56] Indeed, no changes in the transepithelial resistance was noted after HEC gel was applied.[56]

Safety Testing in Colorectal Explant Cultures
The HEC gel was applied to colorectal explant tissues using a polarized system.[51] For safety analysis the MTT assay and histology were performed. No observed reduction in the MTT levels or changes in the tissue architecture were noted.

Efficacy Testing
Analysis of HEC gel activity against HIV showed that it had no protective effect when tested on PBMCs, macrophages, or colorectal explant cultures.[51, 56]

2.7 Condom Compatibility Studies

2.7.1 Tenofovir 1% Gel

The compatibility of tenofovir 1% gel was also tested with three types of lubricated male latex condoms.[44] A matched placebo gel and placebo gel (HEC gel as planned for this trial) were used as comparator gels. The condoms tested were representatives of leading brands on the US market (Trojan® and Durex®) with either silicone or aqueous lubricant. The airburst test was used to evaluate changes in film integrity (strength) and test specimens were measured before and after treatment with the gels to assess changes in strength properties following the application of the three gel preparations. All three gels were shown to be compatible with the above condoms. The compatibility of tenofovir 1% gel with Alatech™ Healthcare (Eufala, AL) male latex silicone lubricated condoms was also evaluated. Tenofovir placebo gel was used as a comparator. The two application treatments of tenofovir 1% gel and matched placebo gel increased airburst volumes by 5 to 6 L compared with the baseline. With an increase in volumes there was a decrease in airburst pressures by 0.2 kPa. This implies a physical change to a more elastic condom. This slight change in physical properties suggests an interaction of the tenofovir 1% gel with the silicone lubricant, but does not indicate that the condoms are unsuitable for use in clinical studies.

2.7.2 2% Nonoxynol-9 Gel

Nonoxynol-9 has been used as a condom coating for many years without any appreciable impact on condom function.

2.7.3 Placebo Gel

The effects of the placebo gel on three brands of condoms including Trojan Enz®, Durex® and Trojan Supra® have been evaluated.[44] The physical properties of each were not significantly affected. Although there were slight increases in airburst volume for all types, and an increase in pressure for synthetic condoms following gel exposure, this was considered normal and not statistically significant.

2.8 Animal Studies

2.8.1 Tenofovir 1% Gel

Pharmacokinetics-Vaginal Administration

Single-dose pharmacokinetics of radio-labeled tenofovir gel in female rabbits has been previously examined (0.5 mL, 1% w/v tenofovir, 5 mg per animal, 50 µCi/kg).[58] Plasma concentrations of radioactivity were highest at the first sample time point (0.5 hour (hr)) and below the level of quantification at 24 hours. Pharmacokinetic parameters including the proportion of dose absorbed systemically could not be estimated, due to the very low plasma concentrations.

In a tissue distribution study using the same radio-labeled tenofovir 1% vaginal gel formulation, dose and strength as the above study, eighteen female rabbits were administered an intravaginal dose using a gavage needle.[44] An additional eighteen rabbits received an intravaginal dose of 3% w/v radio-labeled tenofovir (15 mg per animal). Analysis of vaginal tissue sections found no clear relationship between tissue concentration and dose, with no consistent pattern of distribution. Very little radioactivity was recovered in non-vaginal tissues. Concentrations in blood (0.002 to 0.047 µg-eq/g of tissue) exemplified the variability of distribution of the product although the effect of oral absorption due to grooming behaviors of the animals may have impacted these results.

The pharmacokinetics, excretion and tissue distribution of ^{14}C-PMPA were evaluated in rats following intravaginal administration of an earlier formulation of tenofovir gel containing propylene glycol.[59] Four female rats received a single intravaginal dose administered as an aqueous gel containing 20 mg tenofovir/g. Plasma concentrations of total radioactivity were highly variable; this was attributed to inconsistent retention of the formulation within the vagina, or possibly oral absorption related to grooming. The apparent maximum plasma concentration (C_{max}) for tenofovir occurred at the earliest time point (15 minute), suggesting that absorption from the vagina was relatively rapid. Thereafter plasma concentrations declined with an approximate half-life of 1.6 hrs. The bioavailability of intravaginal tenofovir was estimated by comparison of the observed area under the curve $_{(0-24)}$ (AUC) with historical AUC data for an intravenous (IV) dose of 10 mg/kg tenofovir in rats (9.71 µg hr/mL). The observed systemic bioavailability of intravaginal tenofovir was 7.9%.

In the excretion and distribution study, two groups of four additional rats received a single intravaginal dose of ^{14}C-PMPA (10 mg/kg, 100 µCi/kg) administered as an aqueous gel containing 20 mg tenofovir/g. This study found that much of the dose was lost from the vaginal orifice by leakage. Vaginal tissue contained 0.1% of the dose and less than 0.01% of the dose was recovered in the ovaries and uterus.

The pharmacokinetics (PK) of radio-labeled tenofovir gel was evaluated via plasma and vaginal biopsies collected from four rhesus macaques following single-dose intravaginal administration of tenofovir 1% vaginal gel.[44] Radioactivity was detected starting at 15 minutes post application, with peak concentration of tenofovir in vaginal tissue at 8 hrs and remaining high at 12 hours. No significant radioactivity was detected in whole blood or plasma.

Systemic and vaginal tissue bioavailability was assessed in female white New Zealand rabbits following single and multiple intravaginal doses (twice a day for 7 or 14 days) of 1 mL of tenofovir 1% gel or a single IV solution of 10 mg tenofovir.[44] Animals that were vaginally and intravenously dosed were sacrificed at the following timepoints: 1) 8 hours after single IV dose; 2) 4 hours after single vaginal dose; 3) 8 hours after single vaginal dose; 4) 4 hours after the thirteenth twice-daily vaginal dose; and, 5) 4 hours after the twenty-seventh twice-daily vaginal dose (see table below). After sacrifice, vaginal tissue was rinsed to remove topical tenofovir, and biopsy samples were taken. Both vaginal rinse and vaginal tissue were analyzed for tenofovir content. Systemic absorption following a single intravaginal dose was barely detectable, and only within the first 30 minutes. Multiple intravaginal administrations of tenofovir 1% gel and the single IV administration of 10 mg tenofovir resulted in systemic levels of tenofovir (see Table 2).

Table 2: Tenofovir Bioassay Data

	**Mean 1st Rinse Vaginal Surface (nanogram(ng)/mL)**	**Mean Vaginal Tissue Concentration (ng/g)**	**C_{max} (ng/mL)**	**AUC (0-4 hr) (ng*hr/mL)**
Single IV, 8 hr	**362** (19-990)	**950** (120-5,019)	**10,221**	**4,013** (3,192-4,503)
Single vaginal, 8 hr	**97** (7-415)	**940** (10-7,277)	**3**	--
Single vaginal, 4 hr	**1,441** (2-5,100)	**2,817** (35-11,780)	**5**	--
Twice daily x 7d vaginal, 4 hr	**1,086** (145-4,369)	**3,146** (448-14,429)	**239** (29-808)	**342** (54-1,037)
Twice daily x 14d vaginal, 4 hr	**3,361** (33-8,000)	**11,409** (245-50,102)	**71** (24-197)	**94** (12-229)

Pharmacokinetics-Rectal Administration

Only preliminary assessments of single dose rectal administration of tenofovir 1% gel (PMPA) have been conducted in the setting of a pilot macaque efficacy trial.[60] Plasma samples were assayed for tenofovir concentration by the Clinical Pharmacology and Analytical Chemistry Core of the University of North Carolina Center for Acquired

Immunodeficiency Syndrome (AIDS) Research. Drug concentrations in plasma were determined by a validated high pressure liquid chromatography (HPLC) method with ultraviolet detection.[61] This method utilized a dynamic range of 10 to 10,000 ng/mL, with intra- and inter-day variability of <10% across this range. Total tenofovir concentrations were assayed in tissues using a fully validated HPLC method with mass spectrometry detection.

Analysis of intestinal tissue samples collected at necropsy showed that all tenofovir-dosed animals had measurable concentrations of drug in lysates of colorectal tissue at concentrations between 20.8 and 54.2 µg/g protein but no drug was detected in lysates of homogenates from the small intestine. Tissues from untreated animals acted as negative controls. To indirectly estimate the amount of intracellular phosphorylated tenofovir in tissues, samples were analyzed with (to measure the combination of tenofovir + tenofovir monophosphate + tenofovir diphosphate) and without (to measure tenofovir only) phosphatase hydrolysis. Subtracting the concentration of tenofovir obtained from tissue samples without phosphatase, from the concentration of tenofovir obtained from tissue samples with phosphatase, demonstrated that between 46-75% of total tenofovir in tissues was present as the intracellular monophosphate and diphosphate forms. Based on intracellular data describing tenofovir monophosphate: diphosphate ratios,[62-64] it was estimated that approximately 30-60% of total tenofovir in tissues was present as the intracellular diphosphate form. The relatively low rectal dose of tenofovir gel applied, an average of 10 µg/kg, resulted in a maximum plasma detection level of 0.19%, which was detected 15-minutes after rectal dosing.

Toxicology-Vaginal Administration

The preclinical toxicity of tenofovir gel has been evaluated in 14-day rat and 10-day rabbit vaginal irritation and toxicity studies.[58, 65] Daily intravaginal administration of tenofovir gel produced no vaginal irritation in rats (≤10% tenofovir) and minimal to mild vaginal irritation in rabbits (3% or 10% tenofovir).

14-Day Vaginal Irritation and Toxicity Study of Tenofovir Gel in Rats

Ten female Sprague Dawley rats/group received either 0% (vehicle control), 1%, 3%, or 10% tenofovir gel (2.5% HEC formulation) by intravaginal administration (0.5 mL/dose) once daily for 14 days.[44] There were no mortalities, and no tenofovir-related clinical signs of toxicity or changes in body weight, food consumption, or absolute/relative kidney weights. Individual and mean vaginal (gross) irritation scores for all tenofovir-dosed animals sacrificed at Day 15 were graded as 0 (no erythema or edema); microscopic irritation scores for the vagina, cervix, ovaries, uterine horns, and vulva were graded as 0 (normal histology). No tenofovir-related histopathological effects on the vagina, cervix, ovaries, uterine horns, vulva, or kidneys were observed.

10-Day Vaginal Irritation Study of Tenofovir Gel in Rabbits

The potential irritant effects of tenofovir were evaluated in vaginal tissues of female New Zealand White rabbits using three different gel formulations (2.5% HEC or 1.0 to 2.0% Carbopol® 1342).[44] This study consisted of eleven treatment groups (five rabbits/group) that received one of the following: a sham treatment or Conceptrol® (positive control);

0%, 0.3%, 1.0%, 3.0%, or 10.0% tenofovir formulated in the HEC gel preparation; or 0% or 3.0% tenofovir formulated in a 1.0% or 2.0% Carbopol® 1342 gel preparation. With the exception of the sham dose group, all rabbits received dose formulation (1.0 mL/dose) daily applied topically to the mucosal surface of the vaginal vault for 10 consecutive days. No mortalities and no tenofovir-related clinical signs of toxicity or body weight changes were observed in this study. Group composite vaginal irritation scores for the 10% tenofovir topical gel (HEC formulation), 0% tenofovir (1.0% Carbopol® 1342 formulation), and Conceptrol® (positive control) dose groups were each rated as "mild." Composite vaginal irritation scores rated "minimal" were observed for all other tenofovir, vehicle or sham treatment groups, regardless of the formulation. No unacceptable level of mucosal irritation was observed in any treatment group based on the protocol-derived criteria for this animal model. Generalized erosion and/or ulceration were observed only in animals receiving Conceptrol® positive control (two of five) or the 10% tenofovir topical gel (two of five).

Toxicology-Rectal Administration

14-Day Rectal Irritation Study of Tenofovir Vaginal Gel in Rabbits
Forty New Zealand White rabbits (approximately 10-12 weeks of age and weighing in the range of 2.0 to 2.5 kg at initiation of treatment) were assigned to five dose groups (one sham control, one placebo control and three active test article) consisting of four animals per sex per group under Good Laboratory Practices [(GLP) Pacific BioLabs, Hercules, CA]. The placebo control and active test articles consisted of tenofovir matched placebo gel and three different concentrations (1%, 3%, and 10%) of tenofovir gel respectively. The lubricant for the sham control group was K-Y Jelly from a commercial source.

All female animals were dosed for 14 days and all male animals were dosed for 15 days. Animals in Groups 2 to 5 received 1 mL doses of the respective placebo or test articles via rectal administration for 14/15 consecutive days. A short, soft catheter was attached to a syringe and filled with 1 mL of the appropriate test article. Animals in Group 1, (sham control) underwent the same treatment procedure for 14/15 days with the exception that no dose was administered and the catheter was lubricated with a non-irritating lubricant (K-Y Jelly) prior to insertion. The rectal route of administration was selected as it is the intended clinical route of administration.

Table 3: Rectal Irritation Study in New Zealand White Rabbits

Group	Intervention
1	sham
2	tenofovir placebo gel
3	tenofovir 1% gel
4	tenofovir 3% gel
5	tenofovir 10% gel

The test article, vaginally formulated tenofovir 1% gel, was well tolerated at dose concentrations (1 mL dose volume) of 1% (10 mg/dose), 3% (30 mg per dose) and 10% (100 mg per dose) when administered as a daily rectal dose for 14 days to female

rabbits or 15 days to male rabbits. There was no mortality in this study, and there was only one clinical finding that was potentially study-related: redness at the site of administration in one animal on one day of dosing. There was no evidence of a test article effect on body weight, body weight gain or food consumption over the dose period.

The test article, at the concentrations tested, was without significant effect at the rectal site of administration. Gross pathology at necropsy provided no evidence for tissue damage or inflammation of the rectum or surrounding tissues at the concentrations tested; histolopathological evaluation of the rectum and parts of the colon immediately adjacent to the rectum also showed no effect at the concentrations tested. Each rectum sample was subsectioned into proximal, mid and distal sections (in relation to the site of test article application) for histopathological analysis. Within each section, at least 5 subsections were evaluated for inflammation and other types of lesions. As mentioned, no differences were seen.

Rectal administration of the test articles produced little evidence of test article related systemic effects, despite measurable systemic exposures to tenofovir. At necropsy, gross pathology provided no *in situ* evidence for tissue damage or target organ effects. Changes in several hematology, coagulation and clinical chemistry parameters that reached statistical significance were not considered test article related because they were typically sporadic, not dose-related, and were present in only one gender of rabbit on each occasion. Organ weight changes also reached statistical significance on occasion, but these were also considered not to be test article related for the same reasons cited above, i.e., sporadic and not dose-related. No tissues or organs other than the rectum and colon were examined for histopathological changes.

Rectal application of test articles resulted in measurable systemic concentrations of tenofovir at all dose levels, and after the first dose on Day 1 and the Day 14 dose. Tenofovir exposures were variable on Day 1; however, by Day 14 plasma concentrations were more consistent amongst individual animals and there was a clear dose-related increase in tenofovir exposures in both male and female rabbits. Systemic exposures to tenofovir were comparable in female and male rabbits. Absorption of tenofovir was relatively rapid, with the plasma T_{max} occurring at 1 hr on Day 1 (for most dose groups) and at 2 hr (female rabbits) and 4 hr (male rabbits) on Day 14. Mean C_{max} values on Day 1 ranged from 11.7 ng/mL (Group 4 females) to 59.0 ng/mL (Group 3 females), except Group 3 males where the C_{max} was 1182 ng/mL. Mean C_{max} values on Day 14 ranged from a low of 32.3 ng/mL (Group 3 males) to 265 ng/mL (Group 5 males). The mean T_{max} and C_{max} values for Group 3 males on Day 1 were skewed by one male rabbit with a very high tenofovir plasma concentration at 24 hr post dose of 4210 ng/mL. The elimination half-life for tenofovir could not be determined with accuracy due to the variable exposures on Day 1, and a poorly defined terminal elimination phase on Day 14. For those groups where a half-life could be measured on Day 14, the $t_{1/2}$ for tenofovir ranged from 11.3 to 16.2 hours. It is possible that continued absorption of tenofovir from the rectal site of administration contributed to the inability to accurately measure half-life on Day 14. Tenofovir plasma concentrations

increased in both female and male rabbits with increasing dose. However, the increase in exposure was somewhat less than dose proportional. On Day 14 when tenofovir plasma concentrations were most consistent across individual animals, the decrease in dose-proportional exposure for C_{max}/Dose between Group 3 (10 mg) and Group 5 (100 mg) was 66% and 18% for female and male rabbits, respectively. The decrease for AUC_{last}/Dose between Group 3 and Group 5 was 52% and 32% for female and male rabbits, respectively. There was a marked increase in tenofovir exposure over the 14 days of rectal administration. Accumulation ratios (AUC_{last} Day 14/AUC_{last} Day 1) varied from 7.2 to 23.7 across dose groups.

The No Observed Adverse Effect Level (NOAEL) for rectal administration of test article in this study was greater than the highest concentration tested, i.e., >10% tenofovir in vaginal gel (a 100 mg dose).

Effectiveness-Vaginal Administration

Six independent nonhuman primate studies provided some degree of evidence for efficacy using vaginally administered 1% or 10% gel (see Table 4).[44] Although these data are limited and a powered statistical determination as to the efficacy of tenofovir 1% gel versus 10% cannot be made, empirical examination of the efficacy data identifies tenofovir 1% gel as the lowest efficacious concentration tested when given within two hours of virus challenge. All studies used SIVmac251, a highly infectious SIV isolate, and Indian-origin rhesus macaques (with the exception of study 6). Study 1 demonstrated protection of all four macaques that received 10% tenofovir gel as compared to no protection in the 2 macaques that received placebo gel. Likewise in study 2, 11 of 15 macaques that received 1% or 10% tenofovir gel were protected as compared to no protection in the 5 untreated control macaques that received no gel product. In studies 3, 4, and 5, <100% of the untreated controls were infected making these data problematic to interpret.

Table 4: Use of Topical Tenofovir to Prevent Vaginal Transmission of SIV

Study*	Number of Exposures	Treatment	Time of Administration	Number Infected	Progesterone Pretreatment
1	2	1 mL vehicle	−24 h, 0 h, 24 h, 48 h	2 of 2	No
		10% tenofovir	−24 h, 0 h, 24 h, 48 h	0 of 4	
2	1	untreated control	N/A	5 of 5	No
		10% tenofovir	−24 h , −15 m, +24 h	1 of 5	
		1% tenofovir	−24 h , −15 m, +24 h	1 of 5	
		1% tenofovir	−15 m	2 of 5	
3	1	untreated control	N/A	2 of 5	No
		vehicle	−15 m	1 of 5	
		1% tenofovir	−15 m	1 of 5	
		1% tenofovir	−2 h	3 of 5	
		1% tenofovir	−8 h	1 of 5	
4	1	untreated control	N/A	4 of 5	No
		vehicle	−15 m	2 of 5	
		1 % tenofovir	−15 m	1 of 5	
		1 % tenofovir	−2 h	1 of 5	
		1 % tenofovir	−8 h	2 of 5	
5	1	untreated control	N/A	2 of 5	No
		vehicle	−2 h	2 of 5	
		1 % tenofovir	−2 h	0 of 5	
6	1	1 % tenofovir	−12 h	5 of 8	Yes
		vehicle	−12 h	8 of 8	
		1 % tenofovir	−24 h	8 of 8	
		vehicle	−24 h	8 of 8	
		untreated control	N/A	8 of 8	
		1 % tenofovir	−72 h, −48 h, −24 h	6 of 8	

* All studies were performed with the SIVmac251 isolate of SIV, and female rhesus macaques were inoculated intravaginally. Virus challenges were performed without progesterone pretreatment in Studies 1–5; macaques in Study 6 were pretreated with 30 mg Dep-Provera 30 days prior to viral challenge. The indicated studies were performed by 3 independent investigators with Studies 2, 3, 4, and 5 being performed by the same laboratory.

Study 6 was different from the first five studies in that Chinese-origin rhesus macaques were used and they were pretreated with progesterone before virus challenge to enhance susceptibility to infection and synchronize reproductive cycles. This study was designed to determine whether topical dosing of tenofovir gel could be disassociated from the coital act while remaining an effective microbicide, in a regimen consistent with the long intracellular half-life of the active metabolite, tenofovir diphosphate. A total of 48 macaques, pretreated with a 30 mg dose of depot medroxyprogesterone acetate (DMPA) 30 days prior to viral challenge, were divided into 6 groups of 8 animals each. Group 1 received one topical vaginal dose of tenofovir 1% gel 12 hours prior to one intravaginal viral challenge with a dilution of SIVmac251 stock representing approximately 50 $TCID_{50}$ (50% tissue culture infective dose). In parallel, Group 2 received matched placebo gel. Group 3 received a single dose of tenofovir 1% gel twenty-four hours prior to viral challenge. The matched placebo gel was administered to Group 4 twenty-four hours prior to viral challenge. Group 5 was an untreated control group receiving only the viral challenge. A single dose of tenofovir 1% gel was administered topically to Group 6 animals at 72, 48, and 24 hours prior to viral challenge. Thus, Group 6 animals received 3 consecutive days of gel; Group 4 served as the placebo control for Group 6. Based on plasma viral load, all untreated control animals became infected as did all placebo gel-treated macaques. Three animals were

protected from infection in Group 1 receiving a single dose of tenofovir 1% gel 12 hrs prior to virus exposure. Although no macaques receiving a single dose of tenofovir 1% gel 24 hours prior to virus exposure were protected, two of eight animals in Group 6 receiving multiple doses of tenofovir 1% gel remained uninfected. Infection status was confirmed using virus co-culture, seroconversion and lymph node deoxyribonucleic acid polymerase chain reaction (DNA PCR). These data show 24 of 24 placebo gel-treated or untreated macaques became infected with SIVmac251 while 5 of 24 macaques were protected from SIV infection by vaginally administered tenofovir 1% gel.

Progesterone pretreatment (30 mg DMPA) is used in macaque studies to increase susceptibility to infection by a mechanism thought to involve thinning of the vaginal epithelium. It is generally required to achieve 100% infection in untreated control animals challenged with less infectious Simian/Human Immunodeficiency Virus (SHIV) chimeric viruses. Although animals were pretreated with DMPA in this study but not the previous studies as shown in Table 4 (studies 1−5), this pretreatment may not be required for such a highly infectious virus as SIVmac251. In view of the potent infectivity of this virus, the lack of an endpoint in the animal titration of this stock[66], and increased susceptibility resulting from progesterone pretreatment, it is possible that the amount of virus used was too high, thereby masking any protective effect. Further studies are required to understand the factors that impact protection by intravaginal tenofovir gel in the macaque model.

Effectiveness-Rectal Administration

The rectal application of tenofovir was evaluated for protective efficacy against rectal challenge with simian immunodeficiency virus (SIV) in a well established and standardized pre-clinical macaque model.[60] A total of 20 purpose-bred Indian rhesus macaques were used to evaluate the protective efficacy of topical tenofovir. Six animals received tenofovir 1% gel *per rectum* 15 minutes prior to virus challenge and 3 macaques received tenofovir 1% gel per rectum 2 hours prior to virus challenge, whereas 4 macaques received placebo gel and 4 macaques remained untreated. In addition, 3 macaques were given tenofovir gel 2 hours after virus challenge. Following intrarectal instillation of 20 median rectal infectious doses (MID_{50}) of a non-cloned, virulent stock of $SIV_{mac251/32H}$ all animals were analyzed for virus infection, by virus isolation (VI) from peripheral blood mononuclear cells (PBMC), quantitative proviral DNA load in PBMC, plasma viral ribonucleic acid (vRNA) load by sensitive quantitative competitive (qc)-RT PCR and presence of SIV-specific serum antibodies by ELISA. A significant protective effect was seen (p=0.003; Fisher's Exact Probability test) wherein 8 of 9 macaques given tenofovir *per rectum* either 15 minutes or 2 hours prior to virus challenge were protected from infection (n=6) or had modified virus outcomes (n=2) while 4 of 4 untreated macaques and 3 of 4 macaques given placebo gel were infected, as were 2 of 3 animals receiving tenofovir gel after challenge. Moreover, analysis of lymphoid tissues *post mortem* failed to reveal sequestration of SIV in the protected animals.

Colorectal explants from non-SIV challenged tenofovir treated macaques were resistant to infection *ex vivo*, whereas no inhibition was seen in explants from the small intestine.

Tissue-specific inhibition of infection was associated with the intracellular detection of tenofovir. In colorectal explants from 3 of 4 animals, complete or nearly complete inhibition of virus replication was seen and in the other animals, a high level of variability between replicate samples resulted in lower mean inhibition. In contrast, inhibition of virus replication was not seen in explants from the small intestine suggesting that tenofovir was, at least in part, acting on cells at the virus portal of entry.

Analysis of plasma tenofovir concentration at the time of virus challenge, 15 minutes after gel administration, revealed a strong positive association with protective efficacy. The lowest concentration of plasma tenofovir associated with protection was 119.9 ng/mL. Taking into account estimated plasma volume, protection was associated with as little as 0.11% of the total tenofovir applied; however, this is systemic exposure, rather than local exposure. Moreover, an effect upon plasma viremia was observed with as little as 0.06% of applied tenofovir detected in plasma at 15 minutes. In animals given tenofovir 2 hours prior to virus challenge, plasma tenofovir concentrations at the time of challenge ranged between below the 10 ng/mL limit of detection to 23.3 ng/mL. These results therefore suggest that drug concentration peaks rapidly after rectal dosing. Interestingly ileum/jejunum tissue taken from dosed macaques remained susceptible to infection, and was confirmed by the lack of detectable drug in these tissues. This suggests that secondary distribution to this site is insignificant and supports the importance of comparing an oral, systemically-delivered dose to a topical, locally-delivered dose.

2.8.2 2% Nonoxynol-9 Gel

Toxicology-Vaginal Administration

Galen and colleagues[67] conducted the first study to evaluate the murine mucosal response to repeated microbicide applications. Female BALB/c mice were pre-treated with a 2 mg subcutaneous dose of medroxyprogesterone acetate 5 days prior to a 14-day, once daily, 40 µL, intravaginal dose of Advantage-S (3.5% N-9, Columbia Laboratories Inc., Livingston, NJ), 2% PRO 2000 Gel (Indevus Pharmaceuticals Inc., Lexington, MA) or HEC gel (supplied by the International Partnership for Microbicides). On Day 7, untreated and HEC-treated mice showed no histological changes whereas mice in the N-9 group showed epithelial disruption with some necrosis, and the mice in the PRO 2000 group showed scattered neutrophils but no evidence of epithelial damage or necrosis. The presence of N-9 also resulted in an increase in proinflammatory cytokines and chemokines and a significant increase in transcriptional activators NF-κB and AP-1, relative to the placebo gel. To determine if the noted damage resulted in the likelihood of increased risk to infection, mice were challenged with a sub-lethal dose of herpes simplex virus type 2 (HSV-2), 12 hours following their seventh intravaginal dose of gel. Mice in the N-9 group displayed a significant increase in susceptibility to HSV-2 as compared to the mice in the PRO 2000 and placebo groups. Collectively, these data support the clinical findings that N-9 induces sustained damage resulting in increased susceptibility to infection with HSV in this case.

The effects of repeated applications of two commercially available intravaginal spermicides were evaluated in a monkey model.[68] Effects of Conceptrol® (4% N-9; Ortho Pharmaceutical Corp, Raritan, NJ), benazalkonium chloride (1.2%; Stepan & Co, Northfield, IL), and a 1:1 combination of both products, were evaluated on the vaginal microflora and lower reproductive tract tissues in 14 female pig-tailed macaques. The monkeys received daily vaginal applications (1.5 mL) of either N-9 (n=4), benazalkonium chloride (n=5), or a combination of both products (n=5) for 3 to 4 days. The vaginal wall and cervix were observed by modified colposcopy and a vaginal swab for microbial assessment was taken prior to application of study product. The vaginal swab was repeated 30 minutes after application of study product. Cervical biopsy specimens were also collected in a subset of monkeys. Monkeys in the N-9 arm were treated daily for 3 days, followed by a 2 day resting period, and then treated daily for an additional 3 days. The monkeys in the benazalkonium chloride and the combination arms were treated daily for 4 days.

Colposcopic findings showed evidence of cervical erythema (n=4) and cervical papillae (n=3), and vaginal erythema (n=4) after repeated applications of N-9. Epthilelial disruption, however, was not noted in any of the monkeys in the N-9 arm, but was noted in the monkeys in the benazalkonium chloride arm (n=5) and in the combination arm (n=5).

Toxicology-Rectal Administration

Given the similarities between the rectal flora and epithelial tissue of the pig-tailed macaque (*Macaca nemestrina*) and humans, Patton and colleagues assessed the effects of repeated applications of Conceptrol® (Advanced Care Products, Skillman, NJ, USA) containing 4% N-9 on the microflora and rectal epithelium in a macaque model.[47] The macaques were randomized to one of three groups: Conceptrol®, placebo gel, and no product (8 per group). Macaques in the Conceptrol® and placebo gel groups received daily applications of the study products at 24-hour intervals, for 3 days. Rectal pH swabs, microbiology samples, and rectal lavage specimens were collected from all macaques prior to each application of study product, and again at 15 minutes post-insertion. Final samples were collected on Day 4. The findings showed that repeated applications of Conceptrol® resulted in a reduction of H_20_2-producing microorganisms and decreased detection of black pigmented anaerobic gram negative rods. Furthermore, sheets of epithelium and epithelial cells were observed in the lavage specimens 15 minutes post-insertion of Conceptrol®, and not for the placebo gel and no product groups. The presence of sheets of epithelium in the lavage specimens significantly increased with each successive application of the N-9 containing product. The N-9 product also caused red blood cells to be observed in the lavage specimens. This study points to the imperative need to conduct animal studies to assess the safety and efficacy of microbicides applied rectally, since it is likely that these products will be used rectally regardless of indication for vaginal use, once such a product is approved.

A study assessing the safety of several commercially available sexual lubricants including K-Y® Plus containing 2% N-9 (positive control) as well as phosphate buffered saline (PBS-negative control) and Carraguard, an investigational product from the Population Council, was conducted in 6 to 8 week old BALB/c mice (Charles River,

Wilmington, MA).[69] Cytotoxicity, rectal HSV-2 enhancement, and rectal sloughing assays were performed to assess the effects of the study products on the rectal epithelium. Out of the products used in this study, K-Y Plus was shown to have both the greatest anti-HIV activity and simultaneously cause the greatest amount of disruption to the rectal epithelium.

Effectiveness-Vaginal Administration
In a study by Miller et al.,[70] two different preparations of nonoxynol-9, were found to prevent the genital transmission of SIV (cell-free SIV suspension; 10^4 IV animal infectious dose) among rhesus macaques (colony-bred, > 5 years of age). As little as one mL of nonoxynol-9 contraceptive foam (12.5% vol/vol), administered intravaginally, was enough to prevent transmission in three of the six animals exposed and tested, and, intravaginal administration of as little as one mL of nonoxynol-9 contraceptive gel (3% by weight) prevented the genital transmission of SIV in two of six animals exposed and tested. No striking differences existed in the level of protection provided by either gel or foam preparations, but it is evident that the active ingredient in both was capable of providing some level of protection against genital transmission of SIV.

2.8.3 Placebo Gel

HEC is the thickener in the placebo gel. The results of multiple animal studies (vaginally administered product) have been consistent with the safety of this ingredient.[48] A recently completed rectal study in a macaque model also appears to be consistent with the safety of this ingredient.[71]

Toxicology
Up to 55 IV injections of HEC were given to dogs (dose and number not specified) without causing injury other than that typical of the other water-soluble cellulose ethers.[72] Only transitory changes in the blood picture and the deposition of the material on the intima of the blood vessels were noted. Groups of rats maintained for two years on diets containing HEC (n not specified, up to 5%) did not exhibit any adverse effects. HEC has also been administered to rats in single oral doses as high as 23,000 mg/kg without observed toxic effects (n not specified).

Intraperitoneal administration of unformulated HEC to pregnant mice in a 1% and 4% concentration caused an increase in resorption, but no detectable increase in birth defects.[73] While no epidemiological studies of congenital anomalies in infants born to women exposed to HEC during pregnancy have been reported, the Teratogen Information System (TERIS) considers the magnitude of teratogenic risk to a child born after exposure during gestation to be none.[74]

CF-1 mice (number not specified) pretreated with medroxyprogesterone acetate were administered 0.02 mL of HEC gel vaginally, followed by a 0.01 mL inoculum of 10 intravaginal $dose_{50}$ units of HSV-2 0.3 minutes later.[57] On Day 3, vaginal lavage was cultured on human foreskin fibroblasts, and mice were considered infected if a cytopathic effect was observed after 3 days of incubation. Control animals were treated

similarly but were not administered the test article. Infection rate following pretreatment with HEC gel (90%) was not significantly different from pretreatment with PBS (80%) or from mice given no treatment (% not specified). HEC gel did not enhance susceptibility of mice to HSV-2 when administered 12 hours before vaginal challenge.[48]

A 10-day rabbit vaginal irritation study (10/arm; 2 arms; HEC gel vs. 0.9% saline control) found that the HEC gel was not irritating to the vaginal mucosa of rabbits when dosed daily for 10 days.[48] One animal in the HEC gel group had an instance of vaginal redness (compared to four animals in the saline group), which did not persist and was not evident at the end of the study. Diarrhea, few feces, and soiling of the anogenital area were noted in that animal. Body weight changes were noted to be normal. In 9 of 10 animals, necropsy results were normal. Anogenital soiling was observed in the animal that exhibited erythema during the in-life phase of the study. Histopathological changes observed were similar to those seen in the control group and likely attributable to those that occur as a result of the repeated insertion of a catheter, rather than due to any effect of the test samples.

HEC gel was used as the placebo comparator in a recent rectal safety study of a combination microbicide in a macaque model.[71, 75] A third study arm received no product and served as a negative control. Rectal safety of the active product and HEC gel was evaluated following four daily applications of study products. Rectal flora, pH, and rectal lavage samples were assessed pre- and post-dosing and showed no evidence of toxicity in the macaques that received HEC gel. The infrequent evidence of epithelial sloughing and rare incidence of associated blood cells in rectal lavage samples was similar in the HEC gel and no product arms of this study.

The effect of HEC gel on vaginal transmission of $SHIV_{162p3}$ (10^3 $TCID_{50}$) to rhesus macaques was determined in two separate studies (n = 5, n = 3, respectively).[48] Macaques pretreated with medroxyprogesterone acetate were vaginally administered 1 mL of the HEC gel formulation 15 minutes prior to challenge with 0.5 mL $SHIV_{162p3}$. Investigators monitored total ribonucleic acid (RNA) load in the animal plasma for a total of 8 weeks by means of a standard quantitative RT-PCR. The first study utilized the HEC gel formulation at pH 6.5; the second study utilized a formulation at pH 4.4. In both studies, all macaques were infected, as determined by the presence of viral RNA in circulating blood, regardless of the pH of the formulation.

2.9 Human Clinical Studies

2.9.1 Tenofovir 1% Gel

RMP-02/MTN-006 and MTN-007 will be the first rectal safety studies of tenofovir 1% gel. However, a broad range of reproductive tract studies have been completed, or are ongoing and these data are summarized below.

Pharmacokinetics
Data from "Phase 1 Safety and Acceptability Study of the Vaginal Microbicide Agent PMPA Gel", also known as HPTN 050 has been recently published.[76] Eighty-four (60 HIV negative and 24 HIV positive) women applied either 0.3% or tenofovir 1% gel once or twice daily for 14 days. Systemic absorption was limited (maximum serum levels 3.1-25.8 ng/mL).

In MTN-002, the first microbicide trial to be conducted during pregnancy, 16 women received a single vaginal dose of tenofovir 1% gel prior to elective cesarean section. Tenofovir levels were measured in blood, amniotic fluid, cord blood, endometrial tissue, and placental tissue. Plasma tenofovir levels were compared to historical controls. Study results demonstrated that the PK levels of a single vaginal dose of tenofovir 1% gel in pregnant women was similar to those found in non-pregnant women and that serum tenofovir levels were up to 50 – 100 times less as compared to standard oral dosing.[77] Additionally, tenofovir was shown to get to the fetal compartment with low overall cord levels (~40 times less than oral dosing), but with a similar cord blood: maternal ratio. Overall findings suggest that tenofovir is safe for use in term pregnancy and warrants additional investigation during pregnancy.

Safety
In HPTN 050, the tenofovir 1% gel formulation was well tolerated in both HIV-uninfected and -infected women.[76] Further, 94% of female participants and 81% of male participants indicated they would definitely or probably use tenofovir gel in the future. While a number of participants (92%) reported some type of adverse event (AE), the majority of them were mild (87%) and limited to pruritus (n = 18), erythema (n=14), petechiae/ecchymosis (n = 14), vaginal discharge (n = 13), and burning (n = 10). Only four severe AEs were reported, but of these, only one (lower abdominal pain) was thought to be product-related. Product concentration, sexual activity and HIV status were not associated with a specific AE pattern. No clinically significant systemic toxicity was observed. No serious adverse events (SAEs) were reported.

In a male tolerance study (CONRAD A04-099/IND 73,382), tenofovir 1% gel was well tolerated in men following seven days of once daily exposure, for 6 to 10 hours, to the penis. There were few reported and observed genital findings after product use including mild pain (burning, irritation, discomfort) and pruritis.[44] All observed findings were classified as mild, small in size and requiring no treatment. Reported symptoms were mild, of short duration and resolved by the final visit. There were no noticeable differences between signs and symptoms of genital irritation in the circumcised compared to uncircumcised group.[44, 78]

A Phase 2 study of tenofovir 1% gel (HPTN 059) has completed follow up. This study assessed safety and acceptability of, and adherence to a regimen of tenofovir gel for vaginal use in HIV-uninfected women versus a placebo gel. Exploratory objectives included measurement of vaginal flora characteristics, assessment of the effects of gel on genital cytokine and chemokine expression, and the evaluation of cytokine and chemokine expression to correlate expression with evidence of inflammation, epithelial

disruption and genital symptoms. The study was a four-arm, three-site, randomized, controlled trial comparing gel used once daily and gel used prior to intercourse, to placebo gel, with 6 months gel exposure and follow-up. The study was conducted among 200 women in Pune, India; Birmingham, Alabama, USA; and New York, New York, USA. Participants were sexually active, HIV-uninfected women between ages 18 and 50, but not menopausal or post menopausal. Participants had six months of study gel exposure and follow-up. They were randomized to either once daily or coitally dependent group, and received either tenofovir or placebo gel. Participants received single use unit dose tubes and single-use applicators.

No statistically significant differences were seen between those receiving active and placebo gels in complete blood count (CBC), liver function tests, or renal function tests. Among those using a study gel daily, no participants had pelvic exam findings involving generalized erythema or severe edema or deep epithelial disruption at any follow-up visit during the study. At the Week-24 Visit, no participants had exam findings suggestive of vaginitis, cervicitis, superficial disruption, disrupted blood vessels, or intermenstrual bleeding. Adherence to study gel was high, and was supported by PK data. 79% of women reporting gel use in past 12 hrs had low but detectable plasma tenofovir supporting self-reported adherence data. Daily and coital use was highly acceptable to women. These data suggest a favorable safety and acceptability profile of tenofovir gel, and support routine monitoring for genital findings among women without genital symptoms at six month intervals.[79]

A Phase 2b study of vaginally-administered tenofovir 1% gel use (CAPRISA 004) has recently completed follow-up and data analysis.[80] This study, conducted among sexually active, HIV-uninfected women at an urban and rural site in South Africa, compared the safety and effectiveness of tenofovir 1% gel when use within 12-hours before and after intercourse, versus placebo gel (HEC). Safety assessments as well as HIV and urine pregnancy tests were performed at monthly follow-up visits. Pelvic exams were also performed at quarterly visits.

Study results suggest that vaginally-administered, coitally-dependent use of tenofovir 1% gel is safe. No increases in renal, hepatic, pregnancy-related, or genital AEs were observed. Additionally, tenofovir 1% gel was shown to reduce HIV infection by approximately 39% regardless of sexual behavior, condom use, HSV-2 infection, or urban/rural location. It is important to note, however, that the high acceptability rate (~97%) did not correspond to the average adherence rate (~61%). While these data suggest a favorable safety and effectiveness profile for tenofovir 1% gel, further studies must be done to assess whether more frequent (e.g. daily) dosing will enhance adherence and as a result, effectiveness, without compromising participant safety, and whether tenofovir 1% gel is safe, well-tolerated, and efficacious when administered rectally.

Drug Resistance

In HPTN 050, no new resistance mutations evolved in plasma or cervicovaginal lavage after 14 days of tenofovir gel use, but 3 women had plasma mutations associated with

low level tenofovir resistance identified at both Day 0 and Day 14 (M41L, L210M, ±T215I/Y).[76]

Other Studies of Tenofovir for HIV Prevention

Several other studies of the safety and/or effectiveness of topical tenofovir 1% gel as an HIV prevention strategy are summarized below in Table 5.

Table 5: Other Studies of Tenofovir 1% Gel

Location	Sponsor	Population	Design
USA, Dominican Republic	CONRAD A04-095/IND 73,382	Sexually abstinent women	PK study; single dose and 14-day once or twice-daily.
South Africa, Uganda, USA	DAIDS/MTN-001/IND 55,690	Sexually active women	Phase 2 Adherence and Pharmacokinetics Study of Oral and Vaginal Preparations of Tenofovir
USA	DAIDS IPCP/RMP-02/MTN-006/CONRAD IND 73,382	Sexually abstinent (for active phases of study and for 5 days following biopsy collection) women and men	Phase 1 Rectal PK and Acceptability

Studies examining the safety and/or effectiveness of oral formulations of tenofovir as a prevention strategy are summarized in Table 6 below.

Table 6: PrEP Studies

Location	Sponsor	Population	PrEP Strategy
**Phase 2**			
West Africa (Ghana, Nigeria, Cameroon)	Family Health International	936 high-risk women	TDF
United States	Centers for Disease Control and Prevention (CDC)	400 men who have sex with men	TDF
**Phase 3**			
Thailand	CDC	2,000 injection drug users (~20% women)	TDF
Botswana	CDC	1,200 men and women	FTC/TDF
Peru, Ecuador, Brazil, Thailand, South Africa, United States	NIH<br><br>(iPrEx Study, IND 71,859)	1,400 men who have sex with men (potential expanded sample size of 3,500)	FTC/TDF
Africa	Family Health International	3,800 high-risk women	FTC/TDF
Africa	University of Washington, Gates Foundation	3,900 HIV-1 seronegative partners within HIV-1 discordant couples	FTC/TDF<br>TDF

2.9.2 2% Nonoxynol-9 Gel

Safety-Vaginal Administration

To better understand the molecular basis of N-9 induced inflammation, Fichorova and colleagues[81] evaluated 10 healthy white females at low-risk for HIV infection after single or multiple doses of N-9. Two protocols were used. The first included a single intravaginal dose of 150 mg N-9, while the second included 3 intravaginal doses of 150 mg N-9. Cervicovaginal lavages (CVL) were sampled before and at 12, 36, and 60 hours following administration. CVL was clarified by pelleting the cells and the supernatant was portioned into aliquots and frozen. CVL supernatant was tested for expression of several cytokines and secretory leukocyte protease inhibitor (SLPI) and neutrophil elastase (NE). The cell pellet was suspended in 1 mL PBS and tested for polymorphonuclear cells and the remainder of the cells were placed on a slide to stain for immune cells and a central inflammatory protein, NF-κB, by immunohistochemistry. While a single dose showed modest increases in cytokine levels, multiple doses of N-9 significantly elevated levels of IL-1α, IL-1β, and NE while significantly decreasing SLPI levels as compared to baseline samples. Collectively these data suggest that repeat exposure to N-9 increases the inflammatory response in the female genital tract which has important implications for HIV acquisition.

The vaginal safety of three different concentrations of C31G was assessed in a Phase 1, randomized, double-blind, dose-escalation study.[82] C31G is a surfactant and was under investigation as a possible microbicide and contraceptive. Extra strength (ES) Gynol II® (3% N-9) was used as a comparator since studies have shown that frequent doses of N-9 damage the vaginal epithelium.[83]

A total of sixty-four, low-risk women were randomized to either different concentrations (0.5%, 1.0%, 1.7%) of C31G or to ES Gynol II®. Participants were instructed to apply the study gel once daily for seven consecutive days. If after the first seven days of use, the products appeared safe, participants were asked to apply the study gel twice-daily for seven consecutive days. Safety was assessed by symptomatic irritation, naked eye, and colposcopy. Results of the study showed that the lower concentrations of C31G were less irritating than either the 1.7% C31G or ES Gynol II®. Participants in the Gynol II® arm experienced the most irritation over the course of both treatment periods (87% and 93% respectively) and also accounted for the largest proportion of product -related and non-mild events.

N-9 (Conceptrol®) was also used as the positive control in a Phase 1 safety and colposcopy study of polystyrene sulfonate (PSS) gel conducted by Mauck and colleagues. A total of forty-eight women were randomized to apply 2.5 mL of PSS vehicle, 5% PSS (125 mg), 10% PSS (250 mg), or Conceptrol® (100 mg N-9), once daily for six consecutive days. The primary safety outcome was the proportion of participants who experienced any signs (epithelial changes) or symptoms of genital irritation (itching, pain, and abnormal bleeding). Participants in the Conceptrol® group experienced more signs and symptoms of genital irritation than their counterparts in the PSS group.

In a study designed to evaluate the safety of nonoxynol-9 on the genital mucosa of women from three centers in Malawi and Zimbabwe in preparation for a planned Phase 3 trial of effectiveness of N-9 as a microbicide in lowering the risk of acquiring HIV, 180 women were enrolled and randomized to either N-9 or placebo gel.[84] The women were to insert the gel into the vagina twice daily for 14 days, and returned for examination on days 7 and 14.

The total number of adverse events in the N-9 group was statistically significantly higher than in the placebo group (40% versus 13%, P<0.01). There was a difference in the number of reported symptoms between the two groups, with 38% in the N-9 group and 13% in the placebo group, with the specific symptoms of genital itching being reported more often in the N-9 group as compared with placebo (19% versus 8%, respectively). During the pelvic examinations at the two follow-up visits, erythema was found in 14/89 (16%) of the women on N-9, but only in 2/90 (2%) of the women on placebo. When evaluating epithelial disruptions found at follow-up, no increase in the number of genital ulcers was identified (2% versus 1%), however, there was an increase in lesions other than ulcers (13% versus 3%), and in epithelial sloughing (7% versus 0). Although this study found an increase in the rate of adverse events in the group using 100 mg of N-9 twice daily as compared with the placebo group, it was thought that these were not sufficient to cancel the planned Phase 3 effectiveness study. However, the Phase 3 study was canceled when the investigators considered the negative results from the COL-1492 effectiveness trial[85] which evaluated the use of 52.5 mg N-9, along with these safety results.

In 1998, results were published from a large multicenter randomized trial designed to determine the safety of COL-1492, a spermicidal gel that contains 52.5 mg nonoxynol-9 per dose.[83] The design of the study included both a placebo gel arm and a no-gel control arm, in addition to the active agent arm. Participants randomized to either the placebo or COL-1492 arm were asked to use the gel once daily for 14 consecutive days. Physicians and participants were blinded to study product. Clinical evaluations for patients in all three treatment groups were scheduled at days 0, 7, and 14, at which time a history was obtained and a colposcopy was performed. Additionally, participants were requested to have an evaluation if they experienced any health problems between scheduled visits. Outcomes included reported genital symptoms, incidence of gynecological signs and genital lesions revealed by colposcopy.

For the 534 women enrolled and followed in the study, the incidence of genital symptoms was significantly greater in the COL-1492 group than in the placebo or control group. Women on COL-1492 were also significantly more likely to develop a lesion than women in the other groups (P<0.001), with petechial hemorrhage being the most commonly reported abnormality (20.1% in the COL-1492 group vs. 9% in the placebo group vs. 7.3% in the control group). The incidence of ulceration and abrasion was low overall (1-3%) and was not statistically significantly different among the three groups. Interestingly, when comparing colposcopy results for the placebo gel group with the no treatment control group, the only significant difference found was a higher incidence of edema in the placebo group. There was also a higher incidence of

reported symptoms (particularly vaginal discharge and genital itching), which may be partially due to leakage of the product out of the vagina.

The effect of nonoxynol-9 (N-9) on the female genital tract was examined in a randomized double-blind safety study by Stafford, Ward, Flanagan, et al.[86] In this study, forty female volunteers were randomized to receive either N-9 at a concentration level of 20 mg/mL (100 mg/dose) or a placebo gel. Participants were asked to insert 5 mL of gel on each of 7 consecutive nights, and were examined by a physician at the screening visit, on Day 0 (entry), Day 7 (post gel) and Day 14 (final visit).

This study found that N-9, given in a standard spermicidal dose for 7 consecutive days, was associated with increased symptoms of irritation (50% in the N-9 group vs. 25% in the placebo group) as well as colposcopic evidence of erythema (45% vs. 10 %) and histological evidence of inflammation in the genital tract (35% vs. 10%). Additionally, a temporary reduction in numbers of lactobacilli isolated was seen more frequently in the women using N-9 gel. The inflammatory and microbiologic adverse events found in the women using the placebo gel were thought to be due to the presence of biologically active agents used as preservatives in the placebo gel. The differences in adverse events between the two groups were not statistically significant, likely due to the small sample size. The increase in N-9 induced damage and irritation to the female genital tract as highlighted above, underscores the rationale for inclusion of N-9 as the positive control in MTN-007.

Safety-Rectal Administration

A study conducted by Tabet et al., showed no clinical evidence of rectal or penile disruption or inflammation resulting from 3.5% N-9 use in escalating doses up to 6 weeks.[14] Tabet and colleagues conducted an open-label, Phase 1 study evaluating the safety and toxicity of Advantage 24 (Columbia Research Laboratories, Inc., Rockville Centre, New York, NY) containing, 52.5 mg of N-9 in 1.5g of gel, to rectal and urethral mucosa and penile epithelium in HIV-concordant, monogamous, male couples.

Participants were instructed to apply the N-9 or placebo gel once or twice daily and to have anal sex at least three times a week, within 30 minutes of product application. All participants used the placebo gel for one week, and then used the N-9 gel in escalating doses (up to two applicators twice daily) for 6 weeks. Anoscopies were done after 2, 5, and 6 weeks of N-9 use and rectal biopsy specimens were collected after 5 and 6 weeks of N-9 use for all receptive partners. Low-dose N-9 was not associated with clinical rectal and penile disruption or inflammation, although observations of histological abnormalities were common during use of both the N-9 and placebo gel. It was not clear whether the histological abnormalities were associated with use of the active product, other product components or anal sex. Results of this study were in contrast to other rectal studies discussed below where samples are collected shortly after product application.

Following up on a study that demonstrated enhanced HSV-2 acquisition in mice as a result of rectal application of N-9, Phillips et al., examined rectal lavage samples from 4 participants following rectal application of N-9.[13] K-Y® Plus (Ortho-McNeil

Pharmaceutical, Raritan, NJ) containing 2% N-9, Forplay® (Trimensa Pharmaceuticals, Newbury Park, CA) containing 1% N-9, PC-515 with carrageenan (FMC, Rockland, ME), and methyl cellulose (Mallinckrodt, Paris, KY) were used in this study. In addition to the baseline rectal lavage sample, participants were ask to rectally apply each of the four product formulations followed by rectal lavage at 15 minutes and 8-10 hrs post-insertion, with a minimum of 72 hours between each application and lavage. K-Y® Plus was shown to have caused the greatest amount of epithelial exfoliation, with sheets of epithelium evident in all four lavage specimens, whereas only two of the lavage specimens from Forplay® showed evidence of epithelial exfoliation, although to a much lesser extent than that from use of K-Y® Plus.

Building on a study that assessed the impact of N-9 on the rectal epithelium, Phillips et al.[13] conducted another study of N-9 in 18 participants that included rectal biopsies and rectal lavage. K-Y® Plus (Ortho-McNeil Pharmaceuticals, Raritan, NJ), containing 2% N-9 was the study product, since previous studies[12] have shown it to cause epithelial sloughing at 5 mL. Specimens (via lavage and biopsy) were collected at baseline and again at 15 minutes, 2 hrs, and 8 to 12 hrs after insertion (lavage only). No materials were observed in the baseline collection, but sheets of gut epithelium composed of columnar and goblet cells were observed at 15 minutes after insertion. Significantly fewer materials (degraded cells and bacteria) were observed at the 2 hour time point, and no cellular materials were observed at the 8 hour time point. Results from this study suggest that exfoliation and repair of the rectal epithelium occur within a two hour period.

Thus it appears that rectal administration of N-9 results in mild, but transient epithelial disruption. To capture those events, it is critically dependent on sampling time following application. This information has guided the design of the first rectal microbicide safety study using the vaginal formulation of UC781 as well as the current protocol using the vaginal formulation of tenofovir 1% gel.

Effectiveness-Vaginal Administration

Van Damme and colleagues[85] evaluated the effectiveness of COL-1492 gel (52.5 mg N-9, Columbia Laboratories, New York, NY USA) in a randomized, placebo-controlled, triple-blinded, phase 2/3 trial with a total of 892 female sex workers in Benin, the Ivory Coast, South Africa, and Thailand. Of the 795 women included in the analysis, 376 were randomized to the N-9 arm and 389 to the placebo arm (Replens, Columbia Laboratories, Paris, France). Women were instructed to apply the gel for vaginal intercourse (and again if they had cleaned their vagina after the last act of intercourse). On average, women reported using more than 3.5 applicators per day. It was noted that the risk for HIV acquisition was twice as high for women in the N-9 group as compared to those in the placebo group. The risk for HIV acquisition, however, did not differ among women who reported using the gel less frequently than 3.5 times per day. The results from this study demonstrated that frequent (> 3.5 times/day) N-9 use induced damage to the vaginal epithelium, which resulted in a great risk for acquisition of HIV.

2.9.3 Placebo Gel

Unformulated hydroxyethylcellulose is known to be a non-irritating substance in humans (skin sensitization is unusual), with doses less than 2 g/kg by ingestion not expected to be toxic.[87] No inhalation studies have been conducted, but exposure of humans to the dust in manufacturing operations over many years has not led to any known adverse effects.

Safety-Vaginal Administration
The hydroxyethyl cellulose-based placebo formulation was developed and adopted for use in the HPTN 035 microbicide study, the Phase 2/2B Safety and Effectiveness Study of the Vaginal Microbicides Buffer Gel and 0.5% PRO2000/5 Gel (P) for the Prevention of HIV Infection in Women.

A Phase 1 study of daily vaginal HEC gel exposure was conducted in 2003.[88] In this trial, 30 women were randomized to twice-daily vaginal applications of 3.5 mL of HEC gel or polystyrene sulfonate (PSS) vehicle. The primary objective of this study was to assess and compare the effects of the test articles on symptoms and signs of irritation of the external genitalia, cervix, and vagina as seen on naked eye exam after 7 and 14 days of use including disruption of the epithelium and blood vessels as seen on colposcopy after 14 days of use. Secondary objectives included: 1) an assessment and comparison of differences in vaginal health by evaluating the results of wet mounts, pH, and Gram-stained vaginal smears (Nugent score and neutrophil counts) after 7 and 14 days of use and vaginal cultures after 14 days of use and 2) an assessment of acceptability of the study products after 14 days of use among participants.

Results of this trial indicated that both gels appear safe for use twice a day for 14 days in sexually abstinent women. Two out of 14 women (14.3%) randomized to the HEC group reported at least one symptom of mild severity of genital irritation, which included genital burning, soreness and pelvic pain. A lower proportion of women in the HEC group experienced any evidence (signs and/or symptoms) of genital irritation than in the PSS group. Three out of 14 women in the HEC group (21.4%) had colposcopic findings that included erythema, petechiae and peeling.[88] However, no deep genital disruption was observed in either product group. Minimal changes in wet mounts, pH, Nugent scores, neutrophils, and vaginal flora were observed in both product groups.

Safety-Rectal Administration
A 2-period crossover study of commercially available lubricant gels, by Fuchs and colleagues[89] demonstrated that osmolar properties affected epithelial denudation and product absorption. The gels were made into isosmolar and hyperosmolar mixtures and compounded with a radio-isotope label to address product absorption. Ten healthy male subjects (all MSMs) including 8 seropositive and 2 seronegative men were recruited for the study. All subjects received a 10 mL rectal dose of hyperosmolar ID Glide (Westridge Laboratories, Inc., Santa Ana, CA); 3429 mOsm/kg [pH 4.79]) and a 10 mL rectal dose of an isosmolar preparation of FemGlide (Cooper Surgical Inc., Trumbull, CT)/ID Glide combination gel (283 mOsm/kg [pH 6.77]). Sigmoidoscopy was

performed within 1.5 hours of dosing, with cytobrush sampling at 10 cm and cytobrush and biopsy sampling at 12.5 cm (referred to as 10 cm in results), 40 cm, and 42.5 cm (referred to as 40 cm in results). A paired comparison showed a statistically significant difference in gel concentration between the isosmolar and hyperosmolar products at 10 cm. The median isotope concentration in the isosmolar gel arm was greater at 10 cm than at 40 cm, whereas there was no significant difference at 10 cm and 40 cm in the hyperosmolar gel arm indicating the hyperosmolar gel induced a luminal influx of liquids. The hyperosmolar gel resulted in Grade 3 denudation of the rectal epithelium at 10 cm while the isosmolar gel showed no histological damage. No difference in epithelial structure was observed at 40 cm with either gel. Overall, the results showed epithelial injury is greatest at the site of initial and most concentrated gel exposure which causes mucosal fluid secretion and a dilution of intraluminal gel concentration. While no conclusion could be drawn regarding the timing of repair of epithelial changes in this study, the authors noted that other researchers have reported observations of repair as soon as 2 hours after injury and a resumption to baseline histology at 8 h after insult, prompting the encouragement of future investigation into time-related response, as will be done in MTN-007.

2.10 Justification of No Treatment Arm

MTN-007 will contain three product arms; tenofovir gel, placebo gel, and a 2% N-9 arm. The study will also include a no treatment arm in which mucosal samples will be collected at the same time intervals as the treatment arms; at the Enrollment/Baseline Evaluation Visit, at the Treatment 1 Visit, and at the Final Visit. The purpose of the no treatment arm is to provide data on the baseline levels of the mucosal safety parameters evaluated in the treatment arms. It is acknowledged that the presence of pre-treatment baseline samples for the treatment arms will also provide important comparative data. However, the three evaluations obtained from each participant in the no treatment arm will provide critical data on the biological variability of the mucosal safety parameters. Similar data was generated in the HPTN 056 study where 16 men underwent serial sigmoidoscopy with collection of mucosal samples. However, the HPTN 056 data set is limited as only 4 of the 16 men were HIV negative with a history of receptive anal intercourse. The no treatment control arm may inform identification of any mucosal damage pathways that are not activated by the N-9 arm of the study. Finally, the presence of a no treatment control arm will provide important comparative data with the placebo arm and help determine whether the HEC-based placebo gel is an appropriate control product for future rectal safety studies.

2.11 Study Hypothesis and Rationale

2.11.1 Study Hypothesis

MTN-007 hypothesizes that eight rectal applications of tenofovir 1% gel will be safe, well-tolerated, and acceptable among healthy RAI-abstinent men and women.

2.11.2 Rationale for Rectal Safety Studies

Recently, behavioral publications have emphasized that heterosexual women practice receptive anal intercourse (RAI).[2, 74, 90, 91] Consequently, any vaginal microbicide may well be used in the rectal compartment in association with RAI. Unlike the stratified squamous non-keratinizing epithelium of the vagina, the rectum is lined with a single cell columnar epithelium and represents a biologically distinct compartment for which site specific safety parameters will need to be determined. Developing a rectal safety profile for tenofovir 1% gel vaginal microbicide is an important step in defining the overall safety profile of this product. In addition, successful demonstration of safe and acceptable use of the tenofovir 1% gel formulation rectally may lead to further clinical development of tenofovir 1% gel for rectal use.

There is a well established pathway for the non-clinical and clinical development of vaginal microbicides.[92, 93] Candidate vaginal microbicide products have been evaluated in Phase 1 through Phase 3 studies.[94] To proceed to efficacy studies, candidate microbicides have to pass through a rigorous Phase 1 safety evaluation that routinely includes detailed clinical and colposcopic assessment of the genital compartment. However, no such pathway exists for evaluating the rectal safety of microbicide candidates.

There is relative paucity of safety data for candidate rectal microbicides compared to the vaginal literature. Most studies to date have focused on the surfactant N-9. Initial murine studies showed sloughing of rectal mucosa following application of N-9.[95] Non-human primate studies corroborated this finding and showed sheets of rectal epithelium following a rectal lavage taken 15 minutes after an application of N-9.[47] Human studies followed with similar findings when lavage was performed rapidly after an application of N-9[12, 13]. However, in a rectal safety study that involved daily applications of 3.5% N-9 for 6 weeks, gross mucosal abnormalities were not visualized although mild histological changes on rectal biopsy were reported in 89% of the N-9 group compared to 69% of the placebo arm.[14] These studies set the paradigm for future rectal safety studies as they demonstrated that microbicides that appear safe in the vaginal compartment cannot be assumed to be safe in the rectal compartment.

Histology is preferable to visual inspection which is a coarse measurement of mucosal health for several reasons: 1) the wide spectrum of normal mucosal appearance, 2) inter-observer variability, 3) intra-observer variability.[24] The single-cell rectal lining is easily damaged and quickly repairs. Presence of epithelial cells is normal; mucosal redness, flushing, loss of vascular pattern and even small erosions may all be normal variants and/or a result of colonic preparations. Should any significant abnormalities be noted during the exam, they will be recorded as AEs.

The recently completed UC781 Phase 1 rectal safety study included a broad range of mucosal safety endpoints including: histology, rectal lavage, mucosal cytokines, mucosal T cell phenotype, and fecal calprotectin. Preliminary assessment of the mucosal safety data did not reveal any significant changes associated with product use

(Peter Anton MD, personal communication). These data could be interpreted to suggest that UC781 has a benign safety profile (which may well be true) or that the safety biomarkers used, lacked the sensitivity to identify product-induced mucosal damage. To address this issue, MTN-007 will have a product arm (tenofovir 1% gel), a negative control (placebo gel), and a positive control (2% N-9 gel). All participants will be RAI abstinent for the duration of the study and will receive counseling about the capacity of N-9 to induce mild epithelial disruption.

The rationale for inclusion of an N-9 arm is that we do not have data on the performance characteristics of the immunological biomarkers in the setting of proven inflammation. A recent study has demonstrated the stability of mucosal cytokine and T-cell phenotype in intestinal tissue[11] but did not address changes associated with mucosal inflammation. It is anticipated that MTN-007 participants who are randomized to receive N-9 will experience mild and transient mucosal inflammation that is known to subside within hours of stopping product administration.[12] If the biomarkers in MTN-007 do not increase following administration of N-9, their utility in future microbicide safety studies cannot be recommended. More optimistically, if N-9 associated changes in biomarkers do occur this will be a critical step forward in the development of optimal methods to assess rectal safety of future candidate microbicides.

2.11.3 Rationale for mucosal assays

Repetitive administration of a candidate microbicide to the rectal compartment might result in induction of local immune responses (upregulation of pro-inflammatory cytokines, recruitment of target cells, and/or increased activation of local T-cells), a phenomenon that could be described as mucosal immunotoxicity. Such immunotoxicity could be associated with increased susceptibility to HIV acquisition in sexually active individuals practicing unprotected RAI. As a consequence, MTN-007 has an exploratory objective of determining whether rectal use of tenofovir 1% gel is associated with changes in a broad range of mucosal endpoints.

These endpoints are exploratory but have been previously evaluated in an observational study[11] and in a subsequent rectal safety study of UC781.[15] As previously discussed, repeated rectal administration of UC781 did not result in significant changes in the mucosal parameters that will be evaluated in MTN-007 (Peter Anton MD, personal communication). This finding could be explained by accepting that UC781 has a good rectal safety profile or concluding that the mucosal parameters lacked the sensitivity to identify subtle, but potentially significant, changes in the rectal mucosa. The inclusion of a positive control arm (2% N-9) in MTN-007 is designed to resolve this question.

2.12 Justification of Dosing

MTN-007 is a Phase 1 rectal safety study of the vaginal formulation of tenofovir 1% gel. As a consequence, we have chosen to use the HTI vaginal applicator (HTI Plastics, Lincoln, NE) used in previous vaginal tenofovir gel studies. The primary rationale for

this decision is that until a rectal specific formulation of tenofovir is developed, the only product (and volume of tenofovir gel) available will be the vaginal product.

Choice of the tenofovir 1% vaginal gel concentration is based on both animal and clinical evidence suggesting an appropriate safety profile and potency. Animal and human studies have demonstrated minimal vaginal irritation at this concentration. A rabbit vaginal irritation test identified tenofovir 1% gel as being histopathologically identical to sham or control treatment, while on a qualitative basis 3% gel was more irritating to vaginal epithelia.[44] The tolerability of the 1% gel was confirmed in the HPTN 050 Phase 1 study, the Phase 1 dose ranging study of tenofovir gel (0.3% once daily, then 1.0% once daily, then 0.3% twice daily followed by 1% twice daily).[44, 53, 76] In this study, of the two doses and frequencies studied in the dose finding cohort, the 1% gel applied intravaginally twice daily for 14 days was well-tolerated and was identified as the highest practical dose and frequency for further study in subsequent cohorts.

The second line of evidence is from vaginal transmission inhibition studies performed in nonhuman primates.[44] Six separate studies provided evidence for efficacy of the gel over a range of tenofovir concentrations of 1% to 10%. Although the total data are limited and a powered statistical determination as to the efficacy of tenofovir 1% gel versus 0.3% and 10% cannot be made, empirical examination of the efficacy data identifies tenofovir 1% gel as the lowest efficacious concentration tested when given within two hours of infection. In studies of rectal administration to macaques, 6 of 9 animals given PMPA prior to challenge were protected from overt infection, and virus detection was intermittent or delayed in 2 other macaques. In 4 of 4 untreated macaques and 3 of 4 macaques given placebo gel, virus was recovered at every time-point tested. This indicates a very significant degree of protection ($P < .001$; Fisher's exact test). Virus was isolated on every occasion of testing from 2 of 3 animals where gel was administered 2 hours after virus challenge.[60]

Finally, limited vaginal PK tenofovir data in nonhuman primates demonstrate that tenofovir gel is broadly distributed in vaginal tissues following vaginal application and can penetrate to epithelial tissues.[96] The amount of tenofovir administered by intravaginal application of 4 grams (g) of a 1% dose (40 mg) is highly active against HIV and results in a reduction of plasma HIV ribonucleic acid (RNA) of 1.5 $\log_{10}$ copies/mL after daily administration for 21 days. There are no published studies of drug penetration into human colonic tissue after either rectal or oral administration of tenofovir, though these studies are ongoing. However, colon tissue penetration of tenofovir should exceed tenofovir vaginal tissue penetration given the single columnar colon epithelial layer in contrast to 40 cell layers in the stratified squamous epithelium of the vaginal mucosa.

Significant physiological, histological, and immunological heterogeneity exists within the gastrointestinal tract. The squamous epithelial mucosa of the esophagus is very different to the columnar epithelium of the small intestine. However, even within defined regions of the intestinal tract such as the large intestine there may be regional heterogeneity in histological and/or immunological function. As one example, the

predilection of ulcerative colitis (UC) to always extend from the distal rectum towards the cecum suggests that the left side of the colon has significant differences that make it more susceptible to UC than the right side of the colon. This heterogeneity, although poorly characterized, may have implications for the design of rectal microbicide safety studies. The anorectal tissue directly contacted during RAI (within 10 cm from anal verge) might be quite different to those areas not-directly traumatized but potentially in contact with seminal ejaculate (such as the proximal sigmoid colon).

HPTN 056 was a study of intestinal mucosal immune function.[11] The purpose of the study was to conduct a detailed evaluation of the short-term stability of a range of histological, immunological, and virological mucosal parameters in rectal biopsies obtained from HIV seronegative and seropositive men who were, or were not practicing, RAI. Tissue biopsies were collected at 10 cm and 30 cm from the anal verge. This study demonstrated that there was subtle regional heterogeneity in the expression of mucosal cell phenotypes. T cell populations recognized as target cells for HIV were more common in the 30 cm samples whereas cells bearing the B cell marker CD19 were more common in the 10 cm samples. Cytokine expression displayed less variability although IFN-γ was increased in the 10 cm samples from the individuals with HIV infection. However, these regional differences were felt to be modest and need to be replicated in other studies.

In MTN-007, we will compare biopsies collected at approximately 9 cm from the anal verge using anoscopy with samples collected at 15 cm using flexible sigmoidoscopy. This approach will allow us to evaluate potential regional heterogeneity between sites 9 and 15 cm from the anal margin. It will also allow us to determine whether anoscopy could be used as a primary sampling technique for future rectal microbicide safety studies. This would significantly advance the field by simplifying the sample collection procedures needed to conduct this type of study.

3 OBJECTIVES

3.1 Primary Objective

- To evaluate the safety of tenofovir 1% gel when applied rectally

3.2 Secondary Objectives

- To evaluate the acceptability of tenofovir 1% gel when applied rectally

- To evaluate the safety of placebo gel when applied rectally

- To determine whether use of tenofovir 1% gel is associated with rectal mucosal damage

- To determine whether use of 2% nonoxynol-9 gel (Gynol-II®) is associated with rectal mucosal damage

3.3 Exploratory Objectives

- To determine whether regional heterogeneity exists between mucosal endpoints in samples collected at 9 cm and 15 cm for all parameters examined

- To determine whether there is a correlation between histological abnormality and changes in mucosal biomarkers

4 STUDY DESIGN

4.1 Identification of Study Design

MTN-007 is a Phase 1 randomized, double-blinded, multi-site, placebo-controlled trial. Approximately 60 participants will be randomized to the 4 study arms in a 1:1:1:1 ratio.

4.2 Summary of Major Endpoints

Primary

- Grade 2 or higher adverse events as defined by the Division of AIDS Table for Grading the Severity of Adult and Pediatric Adverse Events, Version 1.0, December 2004 (Clarification dated August 2009) and/or Addenda 1 and 3 (Female Genital and Rectal Grading Tables for Use in Microbicide Studies) to this table

Secondary

- The proportion of participants who at their Final Clinic Visit report via the acceptability questionnaire that they would be very likely to use the candidate microbicide during receptive anal intercourse

- Grade 2 or higher adverse events in the placebo gel arm, as defined by the Division of AIDS Table for Grading the Severity of Adult and Pediatric Adverse Events, Version 1.0, December 2004 (Clarification dated August 2009) and/or Addenda 1 and 3 (Female Genital and Rectal Grading Tables for Use in Microbicide Studies) to this table

- Changes in the following parameters:

    - Epithelial sloughing
    - Intestinal histopathology
    - Intestinal mucosal mononuclear cell phenotype
    - Intestinal mucosal cytokine messenger RNA (mRNA)

- Intestinal mucosal gene expression arrays
- Cytokine profile in rectal secretions
- Fecal calprotectin
- Microflora

Exploratory

- Changes in the following parameters

    - Epithelial sloughing
    - Intestinal histopathology
    - Intestinal mucosal mononuclear cell phenotype
    - Intestinal mucosal cytokine messenger RNA (mRNA)
    - Intestinal mucosal gene expression arrays
    - Cytokine profile in rectal secretions
    - Fecal calprotectin
    - Microflora

4.3 Description of Study Population

The study population will be healthy, HIV-uninfected men and women who meet criteria outlined in Section 5.2 and 5.3.

4.4 Time to Complete Accrual

Accrual is expected to be completed in approximately 5 months.

4.5 Study Groups

Four study arms are planned. A total of approximately 60 men and women will be randomized in a 1:1:1:1 ratio to tenofovir 1% gel, 2% N-9 gel, placebo gel, and no-treatment.

4.6 Expected Duration of Participation

Each participant will be on study for approximately 4 to 11 weeks. The total duration of the study will be approximately 8 months.

4.7 Sites

- Alabama Microbicide CRS, University of Alabama at Birmingham, Birmingham, AL, USA
- Fenway Community Health Center CRS, Boston, MA, USA
- Pitt CRS, University of Pittsburgh, Pittsburgh, PA, USA

5 STUDY POPULATION

5.1 Selection of Study Population

The inclusion and exclusion criteria in Sections 5.2 and 5.3 will be utilized to ensure the appropriate selection of study participants for MTN-007.

5.1.1 Recruitment

Members of the research teams at some of the study sites will recruit men and women from various clinical sites at which they are providing direct patient care to potential study participants. Some sites will also recruit from the greater academic community and related catchment areas or will contact volunteers from previous research studies if those participants have previously signed an authorization permitting this type of contact. All three sites have experience in identifying and recruiting men who have sex with men (MSM) and women with a history of RAI into behavioral and/or clinical studies. Site Institutional Review Board (IRB)-approved media advertisements, telephone scripts, and fliers will be used. These materials will be presented and discussed with the community advisory boards (CAB) at all sites before submission to the local IRBs. Written informed consent will be obtained prior to the initiation of any study-related procedures.

5.1.2 Retention

Each site will establish participant retention procedures. Study site staff members at each site are responsible for developing and implementing site-specific standard operating procedures (SOP) for retention efforts.

5.2 Inclusion Criteria

Individuals who meet the following criteria are eligible for inclusion in the study:

1. ≥ Age of 18 at screening, verified per site SOP

2. Willing and able to provide written informed consent for screening and enrollment

3. HIV-1 uninfected at screening according to the standard DAIDS algorithm in Appendix II

4. Willing and able to communicate in English

5. Willing and able to provide adequate locator information, as defined in site SOP

6. Availability to return for all study visits, barring unforeseen circumstances

7. Per participant report at screening, a history of consensual RAI at least once in the prior year *(Required to assure that participants have a context for the acceptability assessments).*

8. Willing to abstain from insertion of anything rectally, including sex toys, other than the study gel for the duration of study participation

9. Willing to abstain from RAI for the duration of study participation

10. Must agree to use study provided condoms for the duration of the study for vaginal and insertive anal intercourse

11. Must be in general good health

12. At Screening and Enrollment, must agree not to participate in other research studies involving drugs, medical devices, or genital products for the duration of study participation (until all follow-up visits are completed)

In addition to the criteria listed above, female participants must meet the following criteria:

13. Postmenopausal or using (or willing to use) an acceptable form of contraception (e.g., barrier method, IUD, hormonal contraception, surgical sterilization, or vasectomization of male partner). If the female participant has female partners only, the method of contraception will be noted as a barrier method in the study documentation.

5.3 Exclusion Criteria

Individuals who meet any of the following criteria at screening will be excluded from the study:

1. Abnormalities of the colorectal mucosa, or significant colorectal symptom(s), which in the opinion of the clinician represents a contraindication to biopsy (including but not limited to presence of any unresolved injury, infectious or inflammatory condition of the local mucosa, and presence of symptomatic external hemorrhoids

2. At screening: participant-reported symptoms, and/or clinical or laboratory diagnosis of active rectal or reproductive tract infection requiring treatment per current CDC guidelines or symptomatic urinary tract infection (UTI). Infections requiring treatment include symptomatic bacterial vaginosis, symptomatic vaginal candidiasis, other vaginitis, trichomoniasis, Chlamydia (CT), gonorrhea (GC), syphilis, active HSV lesions, chancroid, pelvic inflammatory disease, genital sores or ulcers, cervicitis, or symptomatic genital warts requiring treatment. Note that an HSV-1 or HSV-2 seropositive diagnosis with no active lesions is allowed, since treatment is not required

Note: In cases of non-anorectal GC/CT identified at screening, one re-screening 2 months after screening visit will be allowed

3. Anorectal STI within six months prior to the Screening Visit

4. At screening:
    a. Positive for hepatitis B surface antigen
    b. Hemoglobin < 10.0 g/dL
    c. Platelet count less than 100,000/mm^3
    d. White blood cell count < 2,000 cells/mm^3 or > 15,000 cells/mm^3
    e. *For females:* calculated creatinine clearance less than 60 mL/min by the Cockcroft-Gault formula where creatinine clearance in mL/min (140- age in years) x (weight in kg) x (0.85 for female)/72 x (serum creatinine in mg/dL)
    f. *For males:* calculated creatinine clearance less than 60 mL/min by the Cockcroft-Gault formula where creatinine clearance in mL/min = (140 - age in years) x (weight in kg) x (1 for male)/72 x (serum creatinine in mg/dL)
    g. Serum creatinine > 1.3× the site laboratory upper limit of normal (ULN)
    h. Alanine transaminase (ALT) and/or aspartate aminotransferase (AST) > 2.5× the site laboratory ULN
    i. +1 glucose or +1 protein on urinalysis (UA)
    j. History of bleeding problems

5. History of significant gastrointestinal bleeding in the opinion of the investigator

6. Allergy to methylparaben, propylparaben, sorbic acid, and components of N-9

7. Known HIV-infected partners

8. By participant report at enrollment, history of excessive daily alcohol use (as defined by the CDC as heavy drinking consisting of an average consumption of more than 2 drinks per day for men, and more than 1 drink per day for women), frequent binge drinking or illicit drug use that includes any injection drugs, methamphetamines (crystal meth), heroin, or cocaine including crack cocaine, within the past 12 months

9. Per participant report at screening, anticipated use and/or unwillingness to abstain from the following medications during the period of study participation:
    a. Heparin, including Lovenox®
    b. Warfarin
    c. Plavix® (clopidogrel bisulfate)
    d. Rectally administered medications (including over-the-counter products)
    e. Aspirin
    f. Non-steroidal anti-inflammatory drugs (NSAIDS)
    g. Any other drugs that are associated with increased likelihood of bleeding following mucosal biopsy

10. By participant report at screening, use of post-exposure prophylaxis for HIV exposure, systemic immunomodulatory medications, rectally administered medications, rectally administered products (including condoms) containing N-9, or any investigational products within the 4 weeks prior to the Enrollment/Baseline Evaluation Visit and throughout study participation

11. History of recurrent urticaria

12. Any other condition or prior therapy that, in the opinion of the investigator, would preclude informed consent, make study participation unsafe, make the individual unsuitable for the study or unable to comply with the study requirements. Such conditions may include, but are not limited to, current or recent history of severe, progressive, or uncontrolled substance abuse, or renal, hepatic, hematological, gastrointestinal, endocrine, pulmonary, neurological, or cerebral disease

In addition to the criteria listed above, female participants will be excluded if they meet any of the following criteria:

13. Pregnant at the Enrollment/Baseline Visit

14. Breastfeeding at screening or intend to breastfeed during study participation per participant report.

6 STUDY PRODUCT

6.1 Regimen

Each participant will be randomized to one of three blinded study regimens or to no treatment. Participants randomized to a treatment arm will receive either tenofovir 1% gel, 2% nonoxynol-9 gel or placebo gel.

Treatment arm study participants will receive one dose of study product, at the Treatment 1 visit, under observation.

At the Treatment 2 visit, participants will receive a 7-day supply of study product to administer once daily. All participants will be instructed to insert the entire contents of one applicator rectally at night before bed, or before their longest period of rest.

There will be at least a 7-day washout period between the Treatment 1 and Treatment 2 visits. The participant will have a maximum of 28 days to initiate a consecutive 7-day regimen.

Table 7: Study Product Regimen

Arm	Description	N	Dose, Route, and Frequency
**1**	Tenofovir 1% gel	15	Entire contents of an applicator will be inserted rectally, for a total of 8 doses.
**2**	2% Nonoxynol-9 gel	15	Entire contents of an applicator will be inserted rectally, for a total of 8 doses.
**3**	Placebo gel	15	Entire contents of an applicator will be inserted rectally, for a total of 8 doses.
**4**	No treatment	15	Not Applicable

6.2 Administration

Study staff will instruct participants in proper methods of administering and storing their study product (tenofovir 1% gel, placebo gel or N-9 gel).

At the Treatment 1 Visit, participants will receive one applicator of their assigned study product for self-administration under observation of the site clinician/designee. At the Treatment 2 Visit the participants will receive 8 applicators of their assigned gel for the 7-day administration period. Participants are provided one extra applicator should an applicator not be useable for any reason. During the period of daily administration study participants will be instructed to insert one dose (the entire contents of one applicator) of gel into the rectum once daily throughout the 7-day period. Rectal administration of study product should occur before bedtime, usually in the evening, or the longest period of rest. Participants will be instructed to insert the gel as close to the same time each day as possible.

If a participant misses a dose, she/he must insert rectally the missed dose as soon as possible, unless the next dose is estimated to be due within 6 hours. If the next dose is estimated to be due within 6 hours, the missed dose must be skipped. The next dose will be inserted rectally as originally scheduled.

6.3 Study Product Formulation

6.3.1 Tenofovir 1% Gel

Tenofovir 1% gel (weight/weight) is a gel formulation of tenofovir (PMPA, 9-[(R)-2-(phosphonomethoxy)propyl]adenine monohydrate), formulated in purified water with edetate disodium, citric acid, glycerin, methylparaben, propylparaben, and hydroxyethyl cellulose, with a pH adjusted to 4-5. Tenofovir 1% gel is a transparent, viscous gel that will be filled into applicators to form pre-filled, single-use applicators. Each pre-filled applicator will contain a dose of approximately 4.0 mL of tenofovir 1% gel (equal to 4.4 g).

Tenofovir 1% gel must be stored at controlled room temperature, 25°C (77°F), at all times. Excursions are permitted between 15°C and 30°C (59°F and 86°F).

6.3.2 2% Nonoxynol-9 Gel

Nonoxynol-9 (N-9) will be provided as Gynol II® (Johnson & Johnson, Fort Washington, PA). Gynol II® contains 2% nonoxynol-9 and inactive ingredients including lactic acid, methylparaben, povidone, propylene glycol, purified water, sodium carboxymethyl cellulose, sorbic acid and sorbitol sodium. Nonoxynol-9 is a non-ionic surfactant. It is the active ingredient in many commonly used over-the-counter contraceptive preparations (gels, creams, foams, films, sponges and suppositories) in the United States and worldwide. 2% N-9 will be filled into applicators to form pre-filled, single-use applicators. Each applicator will contain approximately 4.0 mL of 2% N-9 gel for delivery.

Two percent nonoxynol-9 should be stored at 20-25°C (68-77°F). Exposure to extremes of hot or cold should be avoided.

6.3.3 Placebo Gel

The placebo gel, sometimes called the "Universal Placebo Gel" contains hydroxyethyl cellulose as the gel thickener, purified water, sodium chloride, sorbic acid and sodium hydroxide.[48] The gel is isotonic and formulated at a pH of 4.4 to avoid disrupting the normal vaginal pH and has minimal buffering capacity to avoid the inactivation of sexually transmitted pathogens. Hydroxyethyl cellulose, the gelling agent, is used to approximate the viscosity of other microbicide gel candidates. Each pre-filled applicator will contain approximately 4 mL of Placebo gel for delivery.

Placebo Gel should be stored at 25°C (77°F). Excursions are permitted between 15°C and 30°C (59°F and 86°F).

6.4 Study Product Supply and Accountability

6.4.1 Study Product Supply

All study products will be available through Patheon Inc. Patheon Inc. will ship all study products directly to the pharmacist of record (PoR) at each study site. All study products must be stored in the pharmacy.

<u>Tenofovir 1% Gel</u>
Tenofovir 1% gel will be supplied by CONRAD (Arlington, VA, USA). Under direction from CONRAD, Patheon Inc., (Cincinnati, OH USA) which is a contract manufacturing facility, will manufacture the tenofovir 1% gel and analyze/release the gels under cGMP. Patheon Inc. will fill the applicators with tenofovir 1% gel to create pre-filled applicators and package each applicator and plunger in a wrapper.

Placebo Gel
The placebo gel will be supplied by CONRAD (Arlington, VA, USA). Under direction from CONRAD, Patheon Inc., (Cincinnati, OH USA) which is a contract manufacturing facility, will manufacture the placebo gel, and analyze/release the gels under cGMP. Patheon Inc. will fill the applicators with placebo gel to create pre-filled applicators and package each applicator and plunger in a wrapper.

2% Nonoxynol-9 Gel
The 2% nonoxynol-9 (N-9) will be supplied as Gynol II® manufactured by Johnson & Johnson (Fort Washington, PA, USA). DPT Laboratories LTD (San Antonio, TX, USA) will fill the applicators with 2% nonoxynol-9 to create pre-filled applicators and Patheon, Inc., will package each applicator and plunger in a wrapper.

6.4.2 Accountability

The PoR is required to maintain complete records of all study products received from Patheon Inc.

6.4.3 Dispensing

Study products are dispensed only to enrolled participants, upon receipt of a written prescription from an authorized prescriber. At Treatment 2 Visit, depending on the arm of the study to which the participant has been randomized, she/he will receive 8 pre-filled applicators containing tenofovir 1% gel, 2% N-9 gel or placebo gel.

The participants will be provided with sealable bags to collect and store all used and unused applicators, for return to the clinic.

6.4.4 Retrieval of Study Products

Unless the participant needs to replace an applicator, it is anticipated that one unused applicator should remain after the 7 consecutive days of administration. Study participants will be instructed to return all used and unused applicators to the site at the Final Visit. Study staff will count and document the number of returned used and unused applicators. The **used** applicators will be stored at the study site in a biohazard container. The **unused** applicators will be sent to the pharmacy and will be placed in quarantine until returned.

6.4.5 Male Condoms and Lubricant

All participants will receive male condoms and participants in the treatment arm will be offered study specified lubricant to aid with applicator insertion. The condoms and lubricant will be dispensed by the clinic staff, and made available in the clinic.

6.5 Assessment of Participant Adherence

Adherence to product use is crucial to a study that seeks to determine product safety. To encourage adherence, participants will be instructed to apply the product daily before bedtime, usually in the evening or before longest period of rest given that this is likely to result in less leakage and soiling of underwear than is likely to occur when participants are walking around in the course of their daily activities. Therefore, we expect that product administration prior to the longest period of rest will result in better adherence. To monitor adherence, participants will be asked to use a phone reporting system (PRS) after each episode of gel use. To access the PRS, participants call a toll-free number, identify themselves to the system using a unique ID number (corresponding to the participant identification number (PTID)), and then respond to pre-recorded questions on product use and whether there is any comment related to this particular occasion of product use. Responses can be entered by either pressing keys (1 for yes, 2 for no) or by voice response that is understood and registered by the system. Participants receive a small monetary incentive for each call regardless of their report of product use or lack of use; furthermore, a bonus at the end of the seven days is accrued by those who have not missed any day in calling the system. When participants do not call the system within 48 hours, an alert is automatically generated and sent by email to a staff member at Columbia University. The staff member at Columbia University will then contact the study coordinator at the study site who then contacts the participant to inquire about missed calls (if the participant forgot to call) and adherence to the study product regimen. Thus, this system allows monitoring of adherence to calling the PRS on a time-stamped basis. Given that participants are instructed to use the product at bedtime and to call the system immediately after applying the product, the calls are a proxy for compliance with time of application; plus, participants will be reporting whether they used the product, which will constitute one measure of study product adherence. Since participants will be asked to return in separate, sealed bags both used and unused applicators, we will be able to cross validate self-reports, and applicator counts to assess adherence.

6.6 Concomitant Medications

With the exception of medications listed as prohibited, enrolled study participants may use concomitant medications during study participation. All concomitant medications, over-the-counter preparations, vitamins and nutritional supplements, recreational drugs, and herbal preparations reported throughout the course of the study will be recorded on case report forms designated for that purpose.

6.7 Prohibited Medications and Practices

Study participants will be prohibited from using the following medications throughout the study period: heparin (including Lovenox®), warfarin, Plavix® (clopidogrel bisulfate), rectally administered medications (including over-the-counter products), aspirin or NSAIDS, and other drugs that are associated with increased likelihood of bleeding following mucosal biopsy. Furthermore, study participants will be advised not to use the

following products within 4 weeks of the Enrollment/Baseline Evaluation Visit and throughout study participation: post-exposure prophylaxis for HIV exposure, systemic immunomodulatory medications, rectally administered medications, rectally administered products containing N-9, or any other investigational products. Should participants report use of any of these medications or products, PSRT consultation will be requested regarding use of study product. In the event that a participant reports NSAID use prior to a visit requiring endoscopy or biopsy, the study visit should be rescheduled where possible. If not, the determination of action must be decided via an emergency PSRT consultation.

Participants will be advised to refrain from any practices which include rectal insertion of any product including those used during sexual intercourse (sex toys).

7 STUDY PROCEDURES

An overview of the study visit and evaluations schedule is presented in Appendix I. Presented in this section is additional information on visit-specific study procedures. Detailed instructions to guide and standardize procedures across sites are provided in the study-specific procedures manual. Unless otherwise specified, the laboratory procedures listed in this section are performed at the local study site laboratories.

In addition to any Interim Visits that may occur in accordance with guidance outlined in Section 7.9, the following visits should take place for study participants:

- Screening (Visit 1)
- Enrollment/Baseline Evaluation (Visit 2)
- Treatment 1 (Visit 3)
- Follow-Up Phone Assessment (Visit 4)
- Treatment 2 (Visit 5)
- Final (Visit 6)
- Follow-up Phone Assessment/Termination Visit (Visit 7)

Participants randomized to treatment arms will receive a single dose of study product at the Treatment 1 Visit, and then will receive a 7-day supply of study product at the Treatment 2 Visit. Participants will be instructed to insert the study product at night before bed, or before their longest period of rest. There is a 7-day washout period between the Treatment 1 and Treatment 2 Visits. The Enrollment/Baseline Evaluation Visit will be considered Day 0 and will occur no more than 36 days following the Screening Visit.

7.1 Screening Visit

Written informed consent will be obtained before any screening procedures are initiated. For participants who do not meet the eligibility criteria, screening will be discontinued

once ineligibility is determined. For participants who are found to be presumptively eligible based on the evaluations at Screening (listed below), final eligibility will be confirmed at the Enrollment/Baseline Evaluation Visit, which must take place no more than 36 days following the Screening Visit.

Table 8: Screening Visit

**Screening Visit**	
**Component**	**Procedure/Analysis**
**Administrative**	• Obtain written informed consent for Screening Visit<br>• Assign participant ID (PTID)<br>• Collect demographic information<br>• Collect locator information<br>• Assess eligibility<br>• Provide reimbursement for study visit<br>• Schedule next study visit*
**Clinical**	• Collect medical history (including exclusionary medical conditions and medications)<br>• Document pre-existing conditions<br>• Collect menstrual history♀<br>• Collect concomitant medications<br>• Perform physical exam<br>• Perform rectal exam<br>• Provide counseling<br>o HIV pre-and post-test<br>o HIV/STI risk reduction<br>o Contraceptive<br>• Provide condoms<br>• Treat for UTI/RTIs/STIs or refer for other findings*
**Urine**	• Collect urine sample<br>o ♀ Qualitative hCG<br>o Dipstick urinalysis (UA) for protein, glucose, nitrites, and leukocyte esterase<br>o GC and CT by nucleic acid amplification testing (NAAT)
**Blood**	• Collect blood specimens<br>o CBC with differential and platelets<br>o BUN, creatinine (calculate creatinine clearance), ALT, AST<br>o Syphilis rapid plasma reagin (RPR) (confirmatory tests as needed)<br>o HIV-1 serology (confirmatory tests as needed)<br>o Hepatitis B surface antigen (HBsAg)<br>o HSV serology
**Rectal Specimens**	• Rectal swabs<br>o Rectal GC/CT by NAAT

*if indicated ♀ for females of childbearing potential

7.2 Enrollment/Baseline Evaluation Visit

The Enrollment Visit (Day 0) will occur within 36 days of the Screening Visit.

Table 9: Enrollment/Baseline Evaluation Visit (Day 0, Within 36 Days of Screening Visit)

<table>
<tr><th colspan="2">Enrollment/Baseline Evaluation Visit</th></tr>
<tr><th>Component</th><th>Procedure/Analysis</th></tr>
<tr><td>Administrative</td><td>• Obtain written informed consent for Enrollment and Storage and Future Testing of Specimens
• Review/update locator information
• Provide test results
• Eligibility confirmation
• Randomization
• Provide reimbursement for study visit
• Schedule next study visit</td></tr>
<tr><td>Clinical</td><td>• Review/update medical history
• Review/update menstrual history♀
• Review/update concomitant medications
• Perform physical exam
• Perform rectal exam
• Document pre-existing conditions
• Provide counseling
o Adherence (protocol)
o HIV pre-and post-test*
o HIV/STI risk reduction
o Contraceptive
• Provide condoms
• Treat for UTI/RTIs/STIs or refer for other findings*</td></tr>
<tr><td>Behavioral Assessment</td><td>• Administer Baseline Behavioral Questionnaire (BBQ) to all participants
o Instruct participant in use of web-based questionnaire</td></tr>
<tr><td>Urine</td><td>• Collect urine sample
o Qualitative hCG♀
o GC/CT by NAAT*</td></tr>
<tr><td>Blood</td><td>• Collect blood specimens
o Syphilis RPR (confirmatory tests as needed)*
o HIV-1 serology (confirmatory tests as needed)*
o Plasma archive</td></tr>
<tr><td>Rectal Specimens</td><td>• Rectal swabs
o Rectal swab for microflora
o Rectal GC/CT by NAAT*
• Collect rectal sponge specimen for cytokines
• Administer preparatory Normosol-R pH7.4 enema
o Collect effluent for assessment of epithelial sloughing and fecal sample for calprotectin
• Perform high resolution anoscopy and collect:
o Approximately 7 rectal biopsies at approximately 9 cm for histology, cytokine RT PCR, mucosal T cell phenotyping, and mucosal gene expression arrays
• Perform flexible sigmoidoscopy and collect:
o Approximately 7 rectal biopsies at approximately 15 cm for histology, cytokine RT PCR, mucosal T cell phenotyping, and mucosal gene expression arrays</td></tr>
</table>

*If indicated; ♀ for females of childbearing potential

7.3 Treatment 1 Visit

The Treatment 1 Visit will occur between Day 7 and Day 28.

Table 10: Treatment 1 Visit

**Treatment 1 Visit**	
**Component**	**Procedure/Analysis**
**Administrative**	• Review/update locator information<br>• Test results*<br>• Provide reimbursement for study visit<br>• Schedule next study visit<br>• Schedule follow-up phone assessment
**Clinical**	• Review/update medical history<br>• Review/update menstrual history♀<br>• Review/update concomitant medications<br>• Perform physical exam<br>• Perform rectal exam<br>• Provide counseling<br>o Adherence (protocol) including RAI abstinence<br>o HIV/STI risk reduction<br>o Contraceptive<br>• Provide condoms<br>• Record adverse events<br>• Treat for UTI/RTIs/STIs or refer for other findings*
**Urine**	• Collect urine sample<br>o Qualitative hCG♀<br>o GC/CT by NAAT*
**Rectal Specimens**	• Rectal swabs<br>o Rectal swabs for microflora<br>o Rectal GC/CT by NAAT*<br>• Collect rectal sponge specimen for cytokines<br>• Administer preparatory Normosol-R pH 7.4 enema<br>o Collect effluent for assessment of epithelial sloughing and fecal sample for calprotectin<br>• Perform high resolution anoscopy and collect:<br>o Approximately 7 rectal biopsies at approximately 9 cm for histology, cytokine RT PCR, mucosal T cell phenotyping, and mucosal gene expression arrays<br>• Perform flexible sigmoidoscopy and collect:<br>o Approximately 7 rectal biopsies at approximately 15 cm for histology, cytokine RT PCR, mucosal T cell phenotyping, and mucosal gene expression arrays
**Study Product Supply**	• Observe participant administration of single dose of tenofovir 1% gel, 2% N-9 gel, or placebo gel and offer study lubricant to participants in treatment arm

*If indicated; ♀ for females of childbearing potential

7.4 Follow-Up Phone Assessment

A follow-up phone assessment will be scheduled to take place within approximately 24 hours of the Treatment 1 Visit. Study staff will follow-up with participants to inquire about AEs they might experience as a result of study product or procedures performed during the Treatment 1 Visit.

Table 11: Follow-Up Phone Assessment

Follow-up Phone Call	
**Component**	**Procedures/Analysis**
**Clinical**	• Record AEs

7.5 Treatment 2 Visit

The Treatment 2 Visit will occur between Days 14 and 42, but at least 7 days after the Treatment 1 Visit.

Table 12: Treatment 2 Visit

Treatment 2 Visit	
**Component**	**Procedure/Analysis**
**Administrative**	• Review/update locator information<br>• Test results*<br>• Provide reimbursement for study visit<br>• Schedule next study visit
**Clinical**	• Review/update medical history<br>• Review/update menstrual history♀<br>• Review/update concomitant medications<br>• Perform physical exam*<br>• Perform rectal exam*<br>• Provide counseling<br>o HIV/STI risk reduction<br>o Contraceptive<br>o Adherence (protocol and product use)<br>• Provide condoms<br>• Record/update AEs<br>• Treat for UTI/RTIs/STIs or refer for other findings*
**Behavioral Assessment**	• Provide instructions on use of Phone Reporting System to participants randomized to treatment arm
**Urine**	• Collect urine sample<br>o Qualitative hCG♀<br>o GC/CT by NAAT*
**Rectal Specimens**	• Rectal swabs<br>o Rectal GC/CT by NAAT*<br>• Perform high resolution anoscopy and collect*:<br>o Approximately 7 rectal biopsies at approximately 9 cm for histology, cytokine RT PCR, and mucosal T cell phenotyping, and mucosal gene expression arrays
**Study Product Supply**	• Provide supply of tenofovir 1% gel, 2% N-9 gel, or placebo gel and offer study lubricant to participants in treatment arm

*If indicated; ♀ hCG for females of childbearing potential

7.6 Final Clinic Visit

The Final Clinic Visit will occur between Day 21 and Day 63, on the day after the last dose of study product, and no more than 21 days after the Treatment 2 Visit.

Table 13: Final Clinic Visit

**Final Visit**	
**Component**	**Procedure/Analysis**
**Administrative**	• Review/update locator information<br>• Schedule follow-up phone assessment<br>• Provide reimbursement for study visit<br>• Provide test results*
**Clinical**	• Review/update medical history<br>• Review/update menstrual history♀<br>• Review/update concomitant medications<br>• Perform physical exam<br>• Perform rectal exam<br>• Provide counseling<br>o HIV pre-and post-test counseling<br>o HIV/STI risk reduction<br>• Provide condoms<br>• Record/update AEs<br>• Treat for UTI/RTIs/STIs or refer for other findings*
**Behavioral Assessment**	• Administer Product Acceptability Questionnaire (web-based) to participants randomized to treatment arm
**Urine**	• Collect urine sample<br>o Qualitative hCG♀<br>o Dipstick U/A<br>o GC/CT by NAAT*
**Blood**	• CBC with differential and platelets<br>• BUN, creatinine, ALT, AST<br>• Syphilis RPR (with confirmatory tests as needed)<br>• HIV serology (with confirmatory tests as needed)
**Rectal Specimens**	• Rectal swabs<br>o Rectal GC/CT by NAAT*<br>o Rectal microflora<br>• Collect rectal sponge specimen for cytokines<br>• Administer preparatory Normosol-R pH 7.4 enema<br>o Collect effluent for assessment of epithelial sloughing and fecal sample for calprotectin<br>• Perform high resolution anoscopy and collect:<br>o Approximately 7 rectal biopsies at approximately 9 cm for histology, cytokine RT PCR, and mucosal T cell phenotyping, and mucosal gene expression arrays<br>• Perform flexible sigmoidoscopy and collect:<br>o Approximately 7 rectal biopsies at approximately 15 cm for histology, cytokine RT PCR, and mucosal T cell phenotyping, and mucosal gene expression arrays
**Study Product Supply**	• Collect used and unused product from participants in treatment arm

*If indicated ♀ for females of childbearing potential

7.7 Follow-up Phone Assessment Visit/Termination Visit

The Follow-up Phone Assessment Visit will occur between days 28 and 77, targeted to occur 7 days after the Final Clinic Visit and no more than 14 days after the Final Clinic Visit.

Table 14: Follow-Up Phone Assessment Visit

Phone Assessment (Visit 6)	
**Component**	**Procedure/Analysis**
**Clinical**	• Record/update AEs

7.8 Follow-up Procedures for Participants Who Discontinue Study Product

Participants who permanently discontinue study product will not routinely be withdrawn from the study. Rather, every effort will be made to complete all protocol-specified visits and procedures with these participants with the exceptions described below.

7.8.1 Participants Who Become Infected with HIV

Study staff will capture seroconversions on study case report forms (CRFs). Protocol-specified procedures will continue except:

- HIV serology
- Provision of study product
- Adherence counseling (protocol and product use)
- Counseling for HIV/STI risk reduction. Counseling will be modified to address primary and secondary HIV/STI prevention for infected individuals.
- Anoscopy (only if clinically indicated)
- Flexible sigmoidoscopy (only if clinically indicated)

7.8.2 Participants Who Become Pregnant

All protocol-specified study procedures will continue except:

- Provision of study product
- Adherence counseling (protocol and product use)
- Rectal exam
- Anoscopy (only if clinically indicated)
- Flexible sigmoidoscopy (only if clinically indicated)
- Rectal swabs
- Qualitative hCG
- Contraceptive counseling

7.8.3 Participants Who Voluntarily Discontinue Study Product

All protocol-specified study procedures will continue except:

- Provision of study product
- Adherence counseling (protocol and product use)
- Anoscopy (only if clinically indicated)

- Flexible sigmoidoscopy (only if clinically indicated)

7.8.4 Participants Who Are Discontinued from Study Product by the Site Investigator

All protocol-specified study procedures will continue except:

- Provision of study product
- Adherence counseling (protocol and product use)
- Anoscopy (only if clinically indicated)
- Flexible sigmoidoscopy (only if clinically indicated)

Table 15: Early Termination Visit

Early Termination Visit	
**Component**	**Procedure/Analysis**
**Administrative**	• Review/update locator information<br>• Schedule follow-up phone assessment*<br>• Provide reimbursement for study visit<br>• Provide test results*
**Clinical**	• Review/update medical history<br>• Review/update menstrual history♀<br>• Review/update concomitant medications<br>• Perform physical exam<br>• Perform rectal exam<br>• Provide counseling<br>o HIV pre-and post-test counseling<br>o HIV/STI risk reduction<br>• Provide condoms<br>• Record/update AEs<br>• Treat for UTI/RTIs/STIs or refer for other findings*
**Behavioral Assessment**	• Administer Product Acceptability Questionnaire (web-based) to participants in treatment arm
**Urine**	• Collect urine sample<br>o Qualitative hCG♀<br>o Dipstick U/A<br>o GC/CT by NAAT*
**Blood**	• CBC with differential and platelets<br>• BUN, creatinine, ALT, AST<br>• Syphilis RPR (with confirmatory tests as needed)<br>• HIV serology (with confirmatory tests as needed)
**Rectal Specimens**	• Rectal swabs<br>o Rectal GC/CT by NAAT*<br>• Perform high resolution anoscopy and collect:*<br>o Approximately 7 rectal biopsies at approximately 9 cm for histology, cytokine RT PCR, and mucosal T cell phenotyping, and mucosal gene expression arrays<br>• Perform flexible sigmoidoscopy and collect*:<br>o Approximately 7 rectal biopsies at approximately 15 cm for histology, cytokine RT PCR, and mucosal T cell phenotyping, and mucosal gene expression arrays
**Study Product Supply**	• Collect used and unused product from participants in treatment arm

*if indicated; ♀ for females of childbearing potential

7.9 Interim Contacts and Visits

Interim visits may be performed at any time during the study. All interim contacts and visits will be documented in participants' study records and on applicable case report forms.

Some Interim Visits may occur for administrative reasons. For example the participant may have questions for study staff. Other interim contacts and visits may occur in response to AEs experienced by study participants. When interim contacts or visits are completed in response to participant reports of AEs, study staff will assess the reported event clinically and provide or refer the participant to appropriate medical care.

Table 16: Interim Contacts and Visits

Interim Contacts and Visits	
**Component**	**Procedure/Analysis**
**Administrative**	• Review/update locator information<br>• Provide test results*<br>• Schedule next visit*
**Clinical**	• Review/update medical history<br>• Review/update menstrual history♀<br>• Review/update concomitant medications<br>• Perform physical exam*<br>• Perform rectal exam*<br>• Provide counseling<br>o HIV pre-and post-test counseling*<br>o HIV/STI risk reduction<br>• Provide condoms<br>• Record/update AEs*<br>• Treat for UTI/RTIs/STIs or refer for other findings*
**Urine**	• Collect urine sample<br>o Qualitative hCG♀<br>o Dipstick U/A*<br>o GC/CT by NAAT*
**Blood**	• CBC with differential and platelets*<br>• BUN, creatinine, ALT, AST*<br>• Syphilis RPR (with confirmatory tests as needed)*<br>• HIV serology (with confirmatory tests as needed)*
**Rectal Specimens**	• Rectal swabs<br>o Rectal GC/CT by NAAT*<br>• Perform high resolution anoscopy and collect:*<br>o Approximately 7 rectal biopsies at approximately 9 cm for histology, cytokine RT PCR, and mucosal T cell phenotyping, and mucosal gene expression arrays<br>• Perform flexible sigmoidoscopy and collect*:<br>o Approximately 7 rectal biopsies at approximately 15 cm for histology, cytokine RT PCR, and mucosal T cell phenotyping, and mucosal gene expression arrays
**Study Product**	• Collect unused study product from participants in treatment arm*

*if indicated; ♀ for females of childbearing potential

7.10 Final Contact

The Final Clinic Visit for all participants will include laboratory testing for complete blood count, liver panel, creatinine level, and HIV. If all results are not available at the Follow-up Phone Assessment/Termination Visit, a final contact (in person or by telephone – except for HIV test results) may be required to provide these study test results, and post-test counseling, if needed. In addition, for participants who become pregnant during study participation, an additional contact may be required to ascertain the participant's pregnancy outcome. All final contacts will be documented in participant study records.

7.11 Clinical Evaluations and Procedures

The following physical and rectal exam components will be conducted at select visits.

Physical Exam

- Height (may be omitted after the Screening Visit)
- Weight
- Vital signs
  - Temperature
  - Pulse
  - Blood pressure
- General appearance
- Abdomen
- Other components as indicated by participant symptoms

Medical History

- Each participant will be asked about any symptoms or AEs experienced since their previous visit

Rectal Exam and Rectal Specimen Collection

The participant will be positioned in the left lateral decubitus position for the following procedures:

Rectal Exam

- Visual and digital rectal exam: The examiner will conduct a visual examination of the anus and surrounding area and note any abnormality. The examiner will then insert a lubricated gloved finger into the anal canal and sweep around the internal anal circumference.

Rectal Specimen Collection

- Rectal swabs GC/CT, microflora, and sponge collection for cytokines: A lubricated plastic anoscope will be gently and fully inserted (until the lateral 'wings' touch the anal margin) and the obturator removed. Swabs for GC/CT and microflora will be sequentially inserted through the anoscope and placed in contact with the rectal wall, turned through 360 degrees and removed. Next, the

sponge will be inserted through the anoscope and placed in contact with rectum and remain there for 5 minutes. The sponge will then be removed and packaged, then the anoscope will be slowly removed

- Rectal lavage: A 120 mL Normosol-R® (Hospira Inc., Lake Forest, IL) enema will be inserted through the anus and the contents squeezed into the rectum. The participant will hold the fluid in the rectum for approximately 5 minutes then expel it, including stool, into a collection device placed over a toilet bowl
- Flexible sigmoidoscopy and biopsy: A flexible sigmoidoscope will be inserted to 15 cm and biopsies taken using biopsy forceps
- Anoscopic biopsy: A lubricated anoscope will be inserted into the anorectum until the 'wings' touch the anal verge. Biopsies will be taken at 9 cm using biopsy forceps

7.12 Behavioral Measures

There will be three sets of behavioral measures used in this protocol:

Baseline Behavioral Questionnaire

This is a Web-based self-interview that all participants will complete at the Enrollment/Baseline Evaluation Visit at a computer terminal located in the research offices. In addition to demographics, this questionnaire assesses participants' sexual behavior in the prior three months with HIV-negative, positive, or unknown status men and women, including among men, their sexual role—insertive, receptive, or versatile, and frequency of condom use.[97] The assessment includes questions on use of hyperosmolar or hypo-osmolar rectal lubricants, rectal douching prior to sexual intercourse, use of lubricants containing N-9, and other behavioral practices that may affect the anal sphincter or rectal compartment. It also includes questions on frequency of alcohol and drug use in the prior three months and frequency of HIV testing. Finally, the assessment explores participants' attitudes about pre-exposure prophylaxis (PrEP) and post-exposure prophylaxis (PEP), knowledge about microbicides and likelihood of using a microbicide in the future.

Adherence Questionnaire

Adherence will be assessed with PRS to which participants who are randomized to the treatment arm, will be asked to call daily (as described in Section 6.5). Responses to specific questions on product use since the prior call (e.g., "Did you use the product? Y/N) will constitute a measure of adherence. This measurement will be cross validated with used/unused returned applicator counts. In addition, at the Final Clinic Visit, participants will be asked to report on study product use via the Web-based self-interview.

Product Acceptability Questionnaire

This Web-based self-interview will be completed by participants randomized to the treatment arm at the Final Clinic Visit. This questionnaire includes structured and semi-structured questions about the experiences the participant had using the gel rectally, likes and dislikes concerning the gel, the applicator, and the application process, any

changes she/he may have introduced or may wish to introduce in the product used, any problems (e.g., leakage, soiling) she/he may have had, or other product side-effects and how much the participant was bothered by them, and likelihood of using a rectally applied microbicide in the future. This last section has items worded similarly to those of the same section administered at baseline so that we will be able to compare the anticipated likelihood of product use before and after participants become familiar with a product.

7.13 Laboratory Evaluations

7.13.1 Local Laboratory Testing

The local laboratory, site investigator, or designee will run the following, as indicated:

- CBC with platelets and differential
- Syphilis testing by RPR with confirmatory testing as needed
- BUN, creatinine, AST, ALT
- HIV-1 serology, with confirmatory testing as needed
- Urine hCG for women
- Urinalysis
- Urine GC/CT by NAAT
- Hepatitis B surface antigen
- HSV serology
- Rectal GC/CT by NAAT

7.13.2 Network Laboratory Testing

The NL will run the following as indicated:

- Rectal swabs
  - Microflora
- Rectal sponge for cytokines (Luminex)
- Rectal lavage for sloughing
- Rectal biopsies by high resolution anoscopy (HRA) and flexible sigmoidoscopy
  - Cytokines (RT PCR)
  - Gene expression microarrays
  - Phenotyping (flow cytometry)
  - Histology
- Blood Specimens:
  - Plasma archive (to confirm HIV serostatus)

7.13.3 Genova Diagnostics

Fecal specimens will be collected and shipped to Genova Diagnostics for analysis

- Fecal sample (calprotectin)

7.14 Specimen Collection and Processing

Each study site will adhere to the standards of good clinical laboratory practice, the HPTN-MTN Network Laboratory Manual (www.mtnstopshiv.org),DAIDS Laboratory Requirements (http://www3.niaid.nih.gov/LabsAndResources/resources/DAIDSClinRsrch/Laboratories.htm), MTN-007 Study Specific Procedures Manual (www.mtnstopshiv.org), and site standard operating procedures for proper collection, processing, labeling, transport, and storage of specimens at the local laboratory. Specimen collection, testing, and storage at the site laboratories will be documented when applicable using the Laboratory Data Management System (LDMS). In cases where laboratory results are not available due to administrative or laboratory error, sites are permitted to re-draw specimens.

7.15 Specimen Handling

Specimens will be handled in accordance with Requirements for DAIDS Sponsored and/or Funded Laboratories in Clinical Trials (http://www3.niaid.nih.gov/research/resources/DAIDSClinRsrch/PDF/LabPolicy.pdf)

7.16 Storage of Specimens for Future Use

The mucosal biopsy samples will be processed for histology, cell isolation and flow cytometry, and RNA isolation. Histology blocks will be stored at the MTN Core laboratory in Pittsburgh. The cells isolated from the gut biopsies will be consumed by the flow cytometry process and there will be no residual cells. The RNA will be used for RT-PCR amplification in Pittsburgh and for gene array studies at the MTN immunology core in Seattle. It is anticipated that there will be residual RNA stored in both Seattle and Pittsburgh. The residual RNA samples are needed to facilitate additional RT-PCR evaluation of genes identified in the gene array studies. After all protocol testing is complete, any residual samples will be stored based on initial consent from the participant. If the participant did not give consent to store samples after completion of the study, each site will discard specimens according to institute policy.

7.17 Biohazard Containment

As the transmission of HIV and other blood-borne pathogens can occur through contact with contaminated needles, blood, and blood products, appropriate blood and secretion precautions will be employed by all personnel in the drawing of blood and shipping and handling of all specimens for this study as recommended by the CDC and NIH. All biological specimens will be transported using packaging mandated by US Code of Federal Regulations (CFR) 42 Part 72. All dangerous goods materials, including diagnostic specimens and infectious substances, must be transported according to instructions detailed in the International Air Transport Association (IATA) Dangerous Goods Regulations. Biohazardous waste will be contained according to institutional, transportation/carrier, and all other applicable regulations.

8 ASSESSMENT OF SAFETY

8.1 Safety Monitoring

The study site investigators are responsible for continuous close safety monitoring of all study participants, and for alerting the Protocol Team if unexpected concerns arise. A sub-group of the Protocol Team, including the Protocol Chair, CONRAD Medical Officer (MO), DAIDS MO, Protocol Safety Physicians, and Clinical Affairs Safety Associate, serves as the Protocol Safety Review Team (PSRT). The MTN Statistical Data and Management Center (SDMC) prepares routine safety data reports (blinded to treatment assignment) for review by the PSRT, which meets via conference call approximately twice per month during the first 6 months of the study, and then as needed throughout the period of study implementation to review safety data, discuss product use management, and address any potential safety concerns.

8.2 Clinical Data Safety Review

A multi-tiered safety review process will be followed for the duration of this study. The study site investigators are the first layer of this tiered system and are responsible for the initial evaluation and reporting of safety information at the participant level, and for alerting the PSRT if unexpected concerns arise.

Participant safety is also monitored at the Network level through a series of routine reviews conducted by the SDMC Clinical Affairs staff, the PSRT, and study sponsors. Additional special reviews may also be conducted as dictated by the occurrence of certain events.

During the trial, the PSRT will review safety reports (blinded to treatment assignment) and conduct calls to review the data as appropriate. The content, format and frequency of the safety reports will be agreed upon by the PSRT and the SDMC in advance of study implementation. In addition to these routine safety data reviews, the PSRT will convene on an ad hoc basis to make decisions regarding the handling of any significant safety concerns. If necessary, experts external to the MTN representing expertise in the fields of microbicides, biostatistics, and medical ethics may be invited to join the PSRT safety review.

After the product use and the final safety visits are completed, less frequent reporting and safety reviews may be conducted at the discretion of the MTN-007 PSRT.

A Study Monitoring Committee (SMC) has study oversight as no Data Safety Monitoring Board (DSMB) is planned for this study. The SMC provides review of key performance indicators such as participant accrual, participant retention, protocol and intervention adherence, data quality and laboratory quality. As this is a Phase 1 study, the SMC is also charged with reviewing participant safety data.

The SMC will review the study within the first four to six months of the study implementation and at least every six months thereafter (unless review is waived by the SMC Chair).

If at any time, a decision is made to discontinue study gel in all participants, CONRAD, after consultation with the protocol team will inform the US Food and Drug Administration (FDA). The Site PIs will notify the responsible IRBs expeditiously.

8.3 Adverse Events Definitions and Reporting Requirements

8.3.1 Adverse Events

An adverse event is defined as any untoward medical occurrence in a clinical research participant administered an investigational product and which does not necessarily have a causal relationship with the investigational product. As such, an AE can be an unfavorable or unintended sign (including an abnormal laboratory finding, for example), symptom or disease temporally associated with the use of an investigational product, whether or not considered related to the product. This definition is applied to all the study groups, and is applied to all groups beginning from the time of randomization. The term "investigational product" for this study refers to all three study products listed in Section 6 plus the gel applicator.

Study participants will be instructed to contact the study site staff to report any AEs they may experience at any time between enrollment and completion of their participation. In the case of a life-threatening event, they will be instructed to seek immediate emergency care. Where feasible and medically appropriate, participants will be encouraged to seek evaluation where the study clinician is based, and to request that the clinician be contacted upon their arrival. Sites will obtain written permission from the participant to obtain and use records from non-study medical providers to complete any missing data element on a CRF related to an adverse event. All participants reporting an untoward medical occurrence will be followed clinically, until the occurrence resolves (returns to baseline) or stabilizes.

The site IoR will determine AE resolution or stabilization in their best clinical judgment, but may seek DAIDS MO and/or PSRT medical consultation regarding follow-up or additional evaluations of an AE. Study site staff will document in source documents all AEs reported by or observed in enrolled study participants regardless of severity and presumed relationship to study product. Study staff also will record all AEs on case report forms. The DAIDS AE Grading Table Version 1.0, December 2004 (Clarification dated August 2009), Addenda 1 and 3 (Female Genital and Rectal Grading Tables for Use in Microbicide Studies) will be the primary tools for grading adverse events for this protocol. Adverse events not included in that table will be graded by the DAIDS AE Grading Table, Version 1.0 December 2004 (Clarification dated August 2009). In cases where an AE is covered in multiple tables, Addendum 3 (Rectal Grading Table for Use in Microbicide Studies) will be the grading scale utilized. Please note that the grading scale for proteinuria should also be used for grading glycosuria.

Even though RAI abstinence is a requirement during the trial, participants will be encouraged to report to the study clinician any problems experienced by their partners that might be potentially related to study product. If any such problems are reported, study staff should evaluate and document the occurrence. Should any concerns arise with regard to partner safety; the Protocol Chair will advise all study sites on appropriate action.

8.3.2 Serious Adverse Events

Serious adverse events (SAEs) will be defined by the Manual for Expedited Reporting of Adverse Events to DAIDS (Version 2.0, dated January 2010) as AEs occurring at any dose that:

- Results in death
- Is life-threatening
- Results in persistent or significant disability/incapacity
- Is a congenital anomaly/birth defect
- Requires inpatient hospitalization or prolongation of existing hospitalization
  Note: Per ICH SAE definition, hospitalization itself is not an adverse event, but is an outcome of the event. Thus, hospitalization in the absence of an adverse event is not regarded as an AE, and is not subject to expedited reporting. The following are examples of hospitalization that are not considered to be AEs:
  - Protocol-specified admission (e.g. for procedure required by study protocol)
  - Admission for treatment of target disease of the study, or for pre-existing condition (unless it is a worsening or increase in frequency of hospital admissions as judged by the clinical investigator)
  - Diagnostic admission (e.g. for a work-up of an existing condition such as persistent pretreatment lab abnormality)
  - Administrative admission (e.g. for annual physical)
  - Social admission (e.g. placement for lack of place to sleep)
  - Elective admission (e.g. for elective surgery)

Important medical events that may not result in death, be life-threatening, or require hospitalization may be considered a serious adverse drug experience when, based upon appropriate medical judgment, they may jeopardize the patient or subject and may require medical or surgical intervention to prevent one of the outcomes listed above.

8.3.3 Adverse Event Relationship to Study Product

The relationship of all AEs to study product will be assessed per the Manual for Expedited Reporting of Adverse Events to DAIDS (Version 2.0, dated January 2010), the tenofovir gel investigator’s brochure, the N-9 package insert, the placebo gel investigator’s brochure, and clinical judgment. Per the Manual for Expedited Reporting of Adverse Events to DAIDS (Version 2.0, dated January 2010), the relationship categories that will be used for this study are:

- *Related*: There is a reasonable possibility that the AE may be related to the study agent(s)

- *Not related*: There is not a reasonable possibility that the AE is related to the study agent(s)

8.4 Expedited Adverse Event (EAE) Reporting Requirements

Expedited Adverse Event Reporting

Requirements, definitions and methods for expedited reporting of Adverse Events (AEs) are outlined in Version 2.0 of the DAIDS EAE Manual, which is available on the RSC website at http://rsc.tech-res.com/safetyandpharmacovigilance/. The DAIDS Adverse Experience Reporting System (DAERS), an internet-based reporting system, must be used for expedited AE reporting to DAIDS. In the event of system outages or technical difficulties, expedited AEs may be submitted via the DAIDS EAE Form. For questions about DAERS, please contact DAIDS-ES at DAIDS-ESSupport@niaid.gov. Site queries may also be sent from within the DAERS application itself.

Where DAERS has not been implemented, sites will submit expedited AEs by documenting the information on the current DAIDS EAE Form. This form is available on the RSC website: http://rsc.tech-res.com/safetyandpharmacovigilance/. For questions about EAE reporting, please contact the RSC (DAIDSRSCSafetyOffice@tech-res.com).

EAE reporting procedures specific to this protocol are that once the sites have submitted EAEs via DAERS (as above), the RSC Safety Office will also prepare the draft safety reports and send them to the CONRAD and DAIDS MOs for review.

Study sites will be contacted by the DAIDS MO if any further information or clarification is needed after the report is evaluated by CONRAD and DAIDS MOs. The RSC Safety Office will then prepare the final report which will go to CONRAD for signature and submission to the FDA. Copies of this final report will be filed with CONRAD and RSC. Additionally, the RSC Safety Office will distribute safety reports to all DAIDS sites that use products under investigation in this study.

For all EAEs submitted, sites must file an RSC update with the final or stable outcome unless the initial EAE submitted had a final or stable outcome noted already.

EAE Reporting Requirements for this Study

The SAE EAE Reporting Category, as defined in Version 2.0 of the DAIDS EAE Manual, will be used for this study. The study agents for which expedited reporting to CONRAD and the DAIDS MO are required are: tenofovir 1% gel, 2% nonoxynol-9 gel, placebo gel, and the gel applicator.

Grading Severity of Events

The Division of AIDS Table for Grading the Severity of Adult and Pediatric Adverse Events, Version 1.0, December 2004 (Clarification dated August 2009), Addenda 1 and 3 (Female Genital and Rectal Grading Tables for Use in Microbicide Studies) will be the

primary tools for grading adverse events for this protocol. Adverse events not included in those tables will be graded by the DAIDS AE Grading Table Version 1.0, December 2004 (Clarification dated August 2009). In cases where an AE is covered in all tables, the DAIDS AE Grading Table, Version 1.0, December 2004 (Clarification dated August 2009), Addendum 3 (Rectal Grading Table for Use in Microbicide Studies) will be the grading scale utilized.

The DAIDS AE Grading Table, Version 1.0, December 2004 (Clarification dated August 2009), and Addenda 1 and 3 are available on the RSC website at http://rsc.tech-res.com/safetyandpharmacovigilance/.

EAE Reporting Period

- The expedited AE reporting period for this study is defined as the entire study duration for an individual participant (from study enrollment until the participant's final study contact (Follow-Up Phone Assessment Visit/Termination Visit).
- After the protocol-defined AE reporting period, unless otherwise noted, only suspected unexpected serious adverse reactions (SUSARs) as defined in Version 2.0 of the EAE Manual will be reported to DAIDS if the study staff become aware of the events on a passive basis (from publicly available information).

8.5 Pregnancy and Pregnancy Outcomes

Pregnant participants are excluded from this study. Routine urine testing is performed at every study visit. If participants become pregnant at any time during the course of the study, study agents are discontinued, but participants will remain in the study and will continue with these assessments: UA, acceptability assessments, HIV serology, and safety bloods.

Pregnancy-related data will be collected using the pregnancy CRFs for all pregnancies detected during the study. Pregnancy outcomes will not be expeditiously reported to CONRAD and the DAIDS MO unless there is an associated adverse event in the pregnant participant that meets expedited reporting criteria or the pregnancy results in a congenital anomaly meeting the Manual for Expedited Reported of Adverse Events to DAIDS (Version 2.0, January 2010) guidelines for expedited reporting. Fetal losses without congenital anomalies or maternal complications that require expedited reporting will not be expeditiously reported but data will be captured via the pregnancy CRFs.

After the participant's final study contact (Follow-Up Phone Assessment Visit/Termination Visit), pregnancy outcomes that meet criteria for EAE reporting as described above (e.g., maternal complications, congenital anomalies) occurring among participants known to be pregnant at the Final Study Visit will continue be expeditiously reported. The SDMC will prepare and provide to CONRAD a quarterly report on all pregnancies and their outcomes. The SDMC will also prepare an annual summary report of all AEs for the annual IND reports (submitted by CONRAD).

8.6 Social Harms Reporting

Although study sites make every effort to protect participant privacy and confidentiality, it is possible that participants' involvement in the study could become known to others, and that social harms may result (i.e., because participants could become known as HIV-infected or at "high risk" for HIV infection). For example, participants could be treated unfairly or discriminated against, or could have problems being accepted by their families and/or communities. Social harms that are judged by the Investigator of Record to be serious or unexpected will be reported to responsible site IRB at least annually, or according to their individual requirements. In the event that a participant reports social harm, every effort will be made by study staff to provide appropriate care and counseling to the participant, and/or referral to appropriate resources for the safety of the participant as needed. While maintaining participant confidentiality, study sites may engage their CABs in exploring the social context surrounding instances of social harm.

9 CLINICAL MANAGEMENT

Guidelines for clinical management and product discontinuation are outlined in this section.

In general, the site investigator has the discretion to discontinue study product at any time if she/he feels that continued product use would be harmful to the participant or interfere with treatment deemed clinically necessary. Unless otherwise specified below, the investigator should immediately consult the PSRT for further guidance regarding permanent discontinuation.

The site investigator or designee will document all discontinuations on applicable case report forms.

9.1 Grading System

The primary grading system is located in the Rectal Grading Table for Use in Microbicide Studies, which is labeled as Addendum 3 in the DAIDS AE Grading Table, Version 1.0, December 2004 (Clarification dated August 2009), which can be found on the RSC website: http://rsc.tech-res.com/safetyandpharmacovigilance/.

9.2 Dose Modification Instructions

No dose modifications will be undertaken in this study.

9.3 Discontinuation of Study Product(s) in the Presence of Toxicity

Grade 1 or 2
In general, participants who develop a Grade 1 or 2 AE regardless of relatedness to study product that is not specifically addressed below may continue use of study products per protocol.

Grade 3
Participants who develop a Grade 3 AE or toxicity that is not specifically addressed below and is judged to be related to study product should have that study product permanently discontinued.

Grade 4
Participants who develop a Grade 4 AE or toxicity that is not specifically addressed below (regardless of relationship to study product) should have the current study product permanently discontinued.

9.4 General Criteria for Discontinuation of Study Product

Study participants will be permanently discontinued from product use by the Site Investigator or designee in the event of the following:

- Pregnancy
- HIV seroconversion

9.5 Management of Specific Adverse Events

9.5.1 Hemorrhage Following Rectal Mucosal Biopsy

If bleeding continues after the flexible sigmoidoscopy/HRA procedure that is uncontrolled (occurring between bowel movements) and results in the passage of blood clots per rectum, the participant will be referred for assessment in the emergency department of the nearest hospital.

9.5.2 Infection Following Rectal Mucosal Biopsy

The rate of local or systemic infection following anorectal biopsy is very low (< 1 per 1,500 - R Cranston personal communication). Any participant presenting with local or systemic features compatible with infection (fever, localized anorectal pain, anal discharge) will be referred to the emergency department of the nearest hospital.

9.5.3 Perforation of Rectum Following Rectal Mucosal Biopsy

The rate of perforation of a hollow viscus following endoscopic biopsy is less than 0.88:1,000[98]. However, anoscopic rectal sampling is even less likely to perforate a hollow viscus due to sampling being below the reflection of the pelvic peritoneum and

the absence in insufflation for the procedure. Any participant presenting with local or systemic clinical features suggestive of this condition (abdominal pain, swelling, fever) will be referred to the emergency department of the nearest hospital.

9.6 Criteria for Early Termination of Study Participation

Participants may voluntarily withdraw from the study for any reason at any time. The site investigator also may withdraw participants from the study to protect their safety and/or if they are unwilling or unable to comply with required study procedures, after consultation with the PSRP. Participants also may be withdrawn if the study sponsors, government or regulatory authorities (including the Office of Human Research Protections), or site IRBs terminate the study prior to its planned end date. Every reasonable effort is made to complete a final evaluation of participants who withdraw or are withdrawn from the study prior to completing follow-up. Study staff members will record the reason(s) for all withdrawals in participants' study records. In the event that participants who voluntarily withdraw from the study wish to re-join the study, they may resume product use (if applicable) and follow-up through their originally scheduled study exit date.

10 STATISTICAL CONSIDERATIONS

10.1 Overview and Summary of Design

This is a Phase 1 randomized, double-blinded, multi-site, placebo-controlled safety and acceptability study of tenofovir 1% gel. Sixty RAI abstinent, HIV negative adults (male and female) from three sites will be recruited and randomized to four study arms (15 per arm). Participants randomized to a treatment arm will first receive a single dose of the study gel administered rectally in the clinic. Within 30 minutes, a specimen will be obtained to evaluate the mucosal damage by the gel. After a one week recovery period, participants will self-administer rectally outpatient doses once daily for 7 days. They will return to the clinic for acceptability evaluation and specimen collection.

10.2 Study Endpoints

10.2.1 Primary Endpoint

Consistent with the primary study objectives to evaluate the safety of tenofovir 1% gel when applied rectally, the following endpoints will be assessed:

- Grade 2 or higher adverse events as defined by the Division of AIDS Table for Grading the Severity of Adult and Pediatric Adverse Events, Version 1.0, December 2004 (Clarification dated August 2009) and/or Addenda 1 and 3 (Female Genital and Rectal Grading Tables for Use in Microbicide Studies)

10.2.2 Secondary Endpoints

Consistent with the secondary study objective to evaluate the acceptability of tenofovir 1% gel when applied rectally, the following endpoint will be assessed:

- The proportion of participants who at their Final Visit report via the acceptability questionnaire that they would be very likely to use the candidate microbicide during receptive anal intercourse

Consistent with the secondary study objective to evaluate the safety of the placebo gel when applied rectally, the following endpoint be assessed:

- Grade 2 or higher adverse events in the placebo gel arm, as defined by the Division of AIDS Table for Grading the Severity of Adult and Pediatric Adverse Events, Version 1.0, December 2004 (Clarification dated August 2009) and/or Addenda 1 and 3 (Female Genital and Rectal Grading Tables for Use in Microbicide Studies) to this table

Consistent with the secondary study objectives to determine whether use of tenofovir 1% gel and use of 2% nonoxynol-9 gel (Gynol-II®) are associated with rectal mucosal damage, changes in the following endpoints will be assessed:

- Epithelial sloughing
- Intestinal histopathology
- Intestinal mucosal mononuclear cell phenotype
- Intestinal mucosal cytokine messenger RNA (mRNA)
- Intestinal mucosal gene expression arrays
- Cytokine profile in rectal secretions
- Fecal calprotectin
- Microflora

10.2.3 Exploratory Endpoints

Consistent with the exploratory objectives to determine whether regional heterogeneity exists between mucosal endpoints in samples collected at 9 cm and 15 cm for all parameters examined and to determine whether there is a correlation between histological abnormality and changes in mucosal biomarkers, changes in the following endpoints will be assessed:

- Epithelial sloughing
- Intestinal histopathology
- Intestinal mucosal mononuclear cell phenotype
- Intestinal mucosal cytokine messenger RNA (mRNA)
- Intestinal mucosal gene expression arrays
- Cytokine profile in rectal secretions

- Fecal calprotectin
- Microflora

10.3 Accrual, Randomization, Blinding, and Sample Size

The study will recruit a total of 60 RAI abstinent, HIV uninfected men and women from the three study sites. Within each study site, twenty participants will be enrolled and randomized to each of four study arms at a 1:1:1:1 ratio. Based on the prior studies with similar eligibility requirements, each site is expected to enroll 4 participants per month. Therefore accrual is anticipated to take approximately 5 months. The target for retention will be 95% of enrolled participants over the study period. To preserve the study power in the case of discontinuation/non-adherence, additional participants may enroll, at discretion of the protocol team, to replace participants who are discontinued or non-adherent to study product or scheduled study visits. Therefore the total sample size may be slightly exceeding 60 at the end of the study.

The SDMC will provide each study site with a series of numbered, sealed envelopes containing the randomization assignment for each participant. The envelopes will be assigned sequentially by site staff. Each participant will be assigned a product code number. Using a blinded list of product codes and assigned products, the pharmacist at each site will supply the study product. Multiple codes will be utilized to conceal and protect the randomization assignments in this study.

Throughout the period of study implementation and data analysis, neither study staff nor participants will be informed of the participants' random assignments. Study staff and participants will be unblinded after all study visits and data analyses are completed. As described in Section 9, if an investigator is concerned that a participant might be put at an undue risk by continuing product use, the investigator may discontinue the product use by this participant and document the discontinuation. In emergency situations, if a participant experiences an SAE that, in the opinion of the investigator requires unblinding to protect participant safety, the investigator will notify the PSRT to consider and rule upon the request.

For the proposed study sample size, the statistical properties of this study in assessing the safety of study products are summarized below. With 15 participants in each study arm, the probability of observing zero safety events, at least one safety event, and two or more safety events are listed in the following table assuming various true event rates. For instance, if the true rate of a safety endpoint is 5%, the probability of observing that endpoint in at least one participant out of 15 participants is 0.54. A higher true event rate will result in a larger probability to observe at least one event.

Table 17: Power Consideration for MTN-007

Event Rate	Pr(0 event/n=15)	Pr(≥ 1 event/n=15)	Pr(≥ 2 events/n=15)
**1%**	0.86	0.14	0.01
**5%**	0.46	0.54	0.17
**10%**	0.21	0.79	0.45
**15%**	0.09	0.91	0.68
**25%**	≤ 0.01	≥ 0.99	0.92

The statistical properties of this study may also be characterized by the width of the confidence intervals (CI) around observed event rate. The following table presents the exact 95% confidence intervals (Clopper-Pearson method) of the estimated rate when zero, one, or two endpoints are observed among 15 participants:

Table 18: Confidence Intervals

Number of Endpoints	Lower Bound of CI	Upper Bound of CI
**0**	0.0%	16.1%
**1**	0.1%	23.8%
**2**	1.2%	30.4%

Sample Size Consideration for Gene Expression Array Data

We performed a sample size calculation based on rough parameter estimates from existing Illumina array data, using the method by Lee and Whitmore.[99] Suppose there are 30,000 genes that are passing quality control/filtering and are anticipated not to be differentially expressed and suppose there are 1,000 probes that are differentially expressed. Assume the standard deviation of the difference between the $\log_2$ gene expression values across 2 participants is 0.71. We want to control the mean number of falsely positive gene expression changes to be less than 5. To detect a 2-fold change between the treatment and the control arm, we have a power of 0.61, 0.70 and 0.77 for a sample size of 8, 9 and 10 individuals per group, respectively. For comparisons between before and after treatment within the same individuals assuming the standard deviation of the differences of the $\log_2$ gene expression values before and after gel application is 0.4, and other parameters remain the same, the power of detecting a 2-fold change by a paired *t*-test will be approaching 1 for a sample size of 8. These power estimates are approximate, because the standard deviation varies across genes and because we do not know how many genes are actually not differentially expressed.

10.4 Data Analysis

Descriptive statistics and graphics will be used to summarize the characteristics of endpoints among three treatment-randomized groups. For categorical variables, the numbers and the proportions will be tabulated; for continuous variables, the mean, median, standard deviation, and quartiles will be reported. To assess the change of an endpoint from baseline to post-visit levels within a treatment arm, McNemar's test (for categorical variables) or paired *t*-test (for continuous variables) will be used. To assess the difference of certain endpoints after a treatment phase across treatment arms, Chi-square tests will be used for categorical variables with exact *P*-values if the expected

cell count in some stratum is small; *t*-test or linear regression will be used for continuous variables; nonparametric methods such as Wilcoxon rank-sum test may be used if sample size is small and data are non-normal. Generalized linear models will be used to regress continuous or categorical response variables on treatment arm, with or without adjusting for important baseline predictors. The longitudinal data combining endpoints measured at two treatment phases will be analyzed using generalized estimation equations (GEE) with robust variance estimates.

Baseline characteristics will be tabulated for three arms to check any imbalance of randomization. Due to small sample size, formal comparison will not be performed.

10.4.1 Primary and Secondary Analysis on Safety of Tenofovir 1% Gel and Placebo Gel

For the primary safety analysis, we will use per-protocol or modified intention to treat analysis based on the participants who have completed the baseline visit and at least one of two treatment visits. The rationale is that 1) the primary objective of this study is to evaluate the safety of study products. Adverse effects could only be induced by actual exposure to the study products. 2) Due to the small sample size being planned, any missing data generated from discontinuation/non-adherence can be a serious threat to the study power. Therefore we consider replacing participants who are discontinued /non-adherent. The number and the frequency of ≥ Grade 2 adverse events will be tabulated by study arm and treatment visit. Additional safety analyses will also tabulate the number and type of AEs observed overall, and by severity, site, and study arm. AEs that lead to discontinuation of product use and/or study participation will be tabulated separately. At each treatment visit, the rate of safety events will be compared to the baseline within the same treatment arms using McNemar's test, and the event rate will also be compared across treatment arms by Chi-square test. The logistic regression will be used to assess the difference of event rates across arms adjusting for baseline predictors.

10.4.2 Secondary Analysis on Acceptability

Consistent with the secondary study objective to evaluate the acceptability of tenofovir 1% gel when applied rectally, the secondary endpoint is to examine the proportion of participants who at their Final Clinic Visit report in the Product Acceptability Questionnaire that they would be very likely to use the candidate microbicide during receptive anal intercourse. We will calculate the proportion of participants who report high intentionality, operationalized as having a rating in the upper one third of the 10-point Likert scale, to use the product in the future every time they have receptive anal intercourse and compare proportions by study arm using a Chi-square test with exact p-values. Furthermore, we will examine intentionality to use the study gel on occasions when they do not use condoms or if they had to wait 30 minutes after application before having receptive anal intercourse with various types of partners (e.g., lovers, one-night stands, or other partners).

Additionally, to address the secondary study objective, we will conduct non-parametric tests (Kruskal-Wallis non-parametric test), if data are non-normal and sample sizes are unequal across the three conditions, to evaluate whether acceptability assessed at the Final Clinic Visit in the Product Acceptability Questionnaire is different by study condition. Because of insufficient statistical power to detect small or medium differences and the need to be aware of any trends, we will examine the distributions of each acceptability variable by treatment condition. Furthermore, we will inspect effect sizes to estimate how much variance in our measure of acceptability is accounted by the treatment arms. Although many of the individuals who voluntarily enroll in a study like this one would be predisposed to like a product (as measured at baseline by the microbicide intentions scale), the quantitative data will provide descriptive statistics of acceptability after they have had the chance to use the study product for seven days, specifically about the product's characteristics, application process, applicator, as well as the degree to which participants were bothered by leakage, soiling, or other problems related to gel use.

10.4.3 Secondary Analysis on the Mucosal Damage by Tenofovir 1% Gel and 2% Nonoxynol-9 Gel

The association of six sets of mucosal parameters with study products will be examined. Among them epithelial sloughing and histopathology are categorical measures, whereas the mucosal mononuclear cell phenotype, mucosal cytokine profile, weck cell cytokine and fecal calprotectin are continuous measures. All six parameters are measured at baseline, after the Treatment 1 Visit and at the Final Clinic Visit.

Statistical analyses will be first performed to establish the potential immunological biomarkers on microbicide safety in the N-9 gel arm. It is known that the N-9 gel will impose transient inflammation to rectal mucus. Therefore the mucosal parameters, namely various histology measures, cytokines, cell phenotypes or calprotectins, will be compared in biopsies sampled before and after the N-9 single-dose or seven-dose administration. The control arm without gel use will be included in this analysis to adjust for the within-subject fluctuation of these mucosal parameters across 3 sampling time-points. In particular, two sample *t*-test (Wilcoxon rank-sum test if skewed data) will be used to compare the differences of each individual parameter before and after gel use to those of the control arm. Longitudinal data modeling (GEE method) combining all three sampling time-points in both the N-9 arm and the placebo arm will be used to evaluate the differences of biomarkers induced by the N-9 application. Despite the randomization, the imbalance of baseline predictors could occur due to small sample size, additional regression analysis will be performed adjusting for other baseline demographic factors. Those markers that are significantly associated with the N-9 application will be considered as candidate mucosal parameters.

For the tenofovir and placebo gel arms, we will also evaluate the mucosal parameters before and after the gel application, using similar statistical methods as above. If there are mucosal parameters that are changed upon microbicide gel use, these parameters will be candidate markers for mucosal damage; however if there are no difference

detected, we will determine whether there is no mucosal damage or the parameter is not a good marker of mucosal damage by an inspection of results of this parameter from the N-9 arm.

10.4.4 Statistical Analysis of Gene Expression Array Data

We will perform our first statistical analysis once data from the 9 cm biopsies (9 cm up the rectum) will have been collected for a random subset of 8 participants in each group (placebo, tenofovir, N-9 gel, and no treatment), and for the three time points (0 hr, 30 min, 7 days). Microarray raw data will be imported to Beadstudio (v3.2, Illumina) for hybridization quality assessment. Data passing this quality control step will be exported to the *lumi* bioconductor package (www.bioconductor.org) for preprocessing, variance stabilization and normalization.[100, 101] In addition, probes displaying low variability across arrays will be filtered out. Significance analysis of differential expression will be performed by the LIMMA package in the Bioconductor software.[102] The comparison will be made between the 0 hr (baseline) and 30 min or 7 days of tenofovir, N-9, and placebo samples, and between treatment arms and the no treatment arm to identify genes responding differently to the three different compounds. *P*-values and false discovery rates (FDR) will be computed to assess the significance of differential expression. Differentially expressed genes will be defined as those for which the FDR is lower than a cut-off, for example 0.01, and for which the change between baseline and 30 min or 7 days of tenofovir, N-9, or placebo samples is larger than 2-fold or less than 0.5-fold.

Clustering analysis will be performed for the set of genes that show differential expression as identified by the significance analysis. Unsupervised learning methods will be used to identify genes with similar expression patterns. The number of clusters around center points, called centroids, will be determined empirically and the similarity of genes in each cluster will be computed by Euclidean distance. Supervised pattern search will be used to identify genes that share similar or inverse expression patterns to those of a reference gene (also called a profile search). Reference genes will be chosen from the list of biomarkers measured by the RT-PCR and Luminex® assays, based on concordance of expression changes induced by tenofovir, N-9, or placebo exposure between these assays and the microarrays. We may choose additional reference genes, based on findings using the unsupervised learning methods above.

10.4.5 Exploratory Analyses on Regional Heterogeneity of Mucosal Endpoints and Correlation between Histological Abnormality and Changes in Mucosal Biomarkers

To determine whether regional heterogeneity exists between mucosal endpoints, the mucosal parameters will be first compared between samples from 9 cm and 15 cm at each time point, using McNemar's test if the parameter is categorical, paired *t*-test (Wilcoxon signed-rank test if skewed data) if the parameter is continuous. Mixed-effect ANOVA models will be used to evaluate the heterogeneity of two sites across time with subject level modeled as a random effect. Additional analyses will be performed to

evaluate the correlation between mucosal biomarkers and histological abnormality across arms and three time points. At each time point, various cytokines, cell phenotypes or calprotectins will be compared between groups with different levels of histological abnormality, defined by sloughing or histopathology. Longitudinal data modeling (GEE method) combining three time points will be employed to evaluate the collected association of biomarkers with mucosal damage over the study period.

10.4.6 Analysis on Behavioral and Product Adherence Questionnaire

Data collected using the Baseline Behavioral Questionnaire will be primarily descriptive on demographic variables, such as ethnic background, age, education, income; sexual behavior in the prior three months; behavioral practices, such as lubricant and enema use; frequency of HIV testing; and substance use in the prior three months. We will also explore associations between pre-existing practices (i.e., lubricant use) and willingness to use a microbicidal gel.

Product adherence data as tallied by the Phone Reporting System will be analyzed using repeated measures logistic regression to compare gel-use rates (the proportion of outpatient doses used of the seven, once-daily doses prescribed) between treatment arms. GEE will be used to adjust for the within-subject correlations for repeated measures.

11 DATA HANDLING AND RECORDKEEPING

11.1 Data Management Responsibilities

Study case report forms will be developed by the MTN SDMC in conjunction with the protocol team. Quality control reports and queries routinely will be generated and distributed by the SDMC to the study sites for verification and resolution. As part of the study activation process, each study site must identify all case report forms to be used as source documents. Data are transferred to the MTN SDMC, entered, and cleaned using the DataFax data management system.

11.2 Source Documents and Access to Source Data/Documents

All study sites will maintain source data/documents in accordance with Requirements for Source Documentation in DAIDS Funded and/or Sponsored Clinical Trials (http://www3.niaid.nih.gov/LabsAndResources/resources/DAIDSClinRsrch/PDF/SourceDocPolicy.pdf).

Each investigator will maintain, and store securely, complete, accurate and current study records throughout the study. In accordance with US regulations, for each of the three investigational products tested, the investigator will retain all study records for at least two years following the date of marketing approval for the study product for the

indication in which it was studied. If no marketing application is to be filed or if the application is not approved, the records must be retained until two years after the investigation is discontinued and the US FDA is notified.

Study records must be maintained on site for the entire period of study implementation. Thereafter, instructions for record storage will be provided by CONRAD. No study records may be moved to an off-site location or destroyed prior to receiving approval from both DAIDS and CONRAD.

11.3 Quality Control and Quality Assurance

All study sites will conduct quality control and quality assurance procedures for MTN-007 in accordance with Requirements for Clinical Quality Management Plans at DAIDS Funded and/or Supported Clinical Research Sites available at: (http://www3.niaid.nih.gov/research/resources/DAIDSClinRsrch/PDF/QMPPolicy.pdf).

11.4 Study Coordination

CONRAD holds the IND applications for this study. Copies of all regulatory documents submitted to this IND by CONRAD are forwarded to DAIDS, for cross-referencing with other INDs for the study products. Assignment of all sponsor responsibilities for this study will be specified in a Clinical Trials Agreement (CTA) executed by DAIDS and CONRAD.

Study implementation will be directed by this protocol, which may not be amended without prior written approval from the Protocol Chairs and DAIDS Medical Officer. Study implementation will also be guided by a common study-specific procedures manual that provides further instructions and operational guidance on conducting study visits; data and forms processing; specimen collection, processing, and shipping; AE assessment, management and reporting; dispensing study products and documenting product accountability; and other study operations. Standardized study-specific training will be provided to all sites by the MTN CORE, SDMC, NL and other designated members of the Protocol Team.

Close coordination between protocol team members is necessary to track study progress, respond to queries about proper study implementation, and address other issues in a timely manner. The PSRT will address issues related to participant eligibility and AE management and reporting as needed to assure consistent case management, documentation, and information-sharing across sites. Rates of accrual, adherence, follow-up, and AE incidence will be monitored closely by the team as well as the MTN SMC.

12 CLINICAL SITE MONITORING

Study monitoring will be carried out by Pharmaceutical Product Development Inc., (PPD) (Wilmington, NC) in accordance with Requirements for On-Site Monitoring of DAIDS Funded and/or Sponsored Clinical Trials available at:
(http://www3.niaid.nih.gov/research/resources/DAIDSClinRsrch/PDF/OnsiteMonitor_Reqs.pdf).

Study monitors will visit the site to do the following:

- Review informed consent forms, procedures, and documentation
- Assess compliance with the study protocol, Good Clinical Practices (GCP) guidelines, and applicable regulatory requirements (US and non-US), including US Code of Federal Regulations (CFR) Title 45 Part 46 and Title 21 Parts 50, 56, and 312
- Perform source document verification to ensure the accuracy and completeness of study data
- Verify proper collection and storage of biological specimens
- Verify proper storage, dispensing, and accountability of investigational study products
- Assess implementation and documentation of internal site quality management procedures
- Assess site staff training needs

Site investigators will allow study monitors to inspect study facilities and documentation (e.g., informed consent forms, clinic and laboratory records, other source documents, case report forms), as well as observe the performance of study procedures. Investigators also will allow inspection of all study-related documentation by authorized representatives of the MTN CORE, SDMC, NL, CONRAD, NIAID, and US regulatory authorities. A site visit log will be maintained at the study site to document all visits.

13 HUMAN SUBJECTS PROTECTIONS

The investigators will make efforts to minimize risks to participants. Volunteers and study staff members will take part in a thorough informed consent process. Before beginning the study, the investigators will have obtained IRB approval and the protocol will have been submitted to the FDA. The investigators will permit audits by the NIH, CONRAD, the FDA or any of their appointed agents.

13.1 Institutional Review Boards

Each participating institution is responsible for assuring that this protocol and the associated site-specific informed consent documents and study-related documents (such as participant education and recruitment materials) are reviewed by an

Institutional Review Board (IRB) responsible for oversight of research conducted at the study sites. Any amendments to the protocol must be approved by the responsible IRB and DAIDS prior to implementation.

Subsequent to the initial review and approval, the responsible IRBs must review the study at least annually. Each investigator will make safety and progress reports to the IRBs at least annually and within three months after study termination or completion. These reports will include the total number of participants enrolled in the study, the number of participants who completed the study, all changes in the research activity, and all unanticipated problems involving risks to human subjects or others. In addition, the results of all SMC reviews of the study will be provided to the IRBs. Study sites will submit documentation of continuing review to the DAIDS Protocol Registration Office (PRO) in accordance with the DAIDS Protocol Registration Policy and Procedures Manual.

13.2 Protocol Registration

Prior to implementation of this protocol, and any subsequent full version amendments, each site must have the protocol and the protocol consent form(s) approved, as appropriate, by their local institutional review board (IRB)/ethics committee (EC) and any other applicable regulatory entity (RE). Upon receiving final approval, sites will submit all required protocol registration documents to the DAIDS PRO at the Regulatory Support Center (RSC). The DAIDS PRO will review the submitted protocol registration packet to ensure that all of the required documents have been received.

Site-specific informed consent forms (ICFs) *WILL NOT* be reviewed or approved by the DAIDS PRO, and sites will receive an Initial Registration Notification when the DAIDS PRO receives a complete registration packet. Receipt of an Initial Registration Notification indicates successful completion of the protocol registration process. Sites will not receive any additional notifications from the DAIDS PRO for the initial protocol registration. A copy of the Initial Registration Notification should be retained in the site's regulatory files.

Upon receiving final IRB/EC and any other applicable RE approval(s) for an amendment, sites should implement the amendment immediately. Sites are required to submit an amendment registration packet to the DAIDS PRO at the RSC. The DAIDS PRO will review the submitted protocol registration packet to ensure that all the required documents have been received. Site-specific ICF(s) *WILL NOT* be reviewed and approved by the DAIDS PRO and sites will receive an Amendment Registration Notification when the DAIDS PRO receives a complete registration packet. A copy of the Amendment Registration Notification should be retained in the site's regulatory files.
For additional information on the protocol registration process and specific documents required for initial and amendment registrations, refer to the current version of the DAIDS Protocol Registration Manual.

13.3 Risk-Benefit Statement

13.3.1 Risks

General

Phlebotomy may lead to discomfort, feelings of dizziness or faintness, and/or bruising, swelling and/or infection. Disclosure of STI status may cause sadness or depression in volunteers. Participation in clinical research includes the risks of loss of confidentiality and discomfort with the personal nature of questions.

Although study sites make every effort to protect participant privacy and confidentiality, it is possible that participants' involvement in the study could become known to others, and that social harms may result (i.e., because participants could become known as HIV-infected or at "high risk" for HIV infection). For example, participants could be treated unfairly or discriminated against, or could have problems being accepted by their families and/or communities.

Participants in sites requiring partner notification in response to diagnosed STIs or HIV infection could have problems in their relationships with their sexual partners. Participants also could have problems in their partner relationships associated with use or attempted use of study products. In addition, participants could misunderstand the current experimental status of the study gels (i.e., their unknown safety and unproven efficacy) and as a result increase their HIV risk behaviors while in the study.

Data on participant risk behaviors and the occurrence of other potential social harms are collected from all participants on a quarterly basis. The Protocol Team monitors trends in risk behaviors over time based on these data, as well as the occurrence of other potential social harms, and takes any required follow-up action. This information also is reported to the SMC.

Participants will also be counseled on the importance of remaining RAI abstinent throughout the duration of the study to minimize risk to the rectum given the use of N-9 in this study combined with the unknown effects of the other study gels on the rectum.

Anoscopy

Anoscopy is a commonly practiced medical procedure and the procedures done in this trial will not involve any unusual risks or discomforts. The risk associated with these procedures is a small amount of bleeding. The biopsies are painless and heal quickly within 3 to 5 days. On extremely rare occasions, biopsies may lead to pain, bleeding or perforation of the gastrointestinal tract, or infection. Perforation occurs approximately less than once out of every 1,000 procedures. If this extremely rare complication occurs, antibiotics and surgery to repair the tear may be necessary.

Flexible Sigmoidoscopy

Flexible sigmoidoscopy is a commonly practiced medical procedure and the endoscopic procedures done in this trial will not involve any unusual risks or discomforts. The risks

associated with these procedures include mild discomfort and the feeling of having a "bloated stomach". Endoscopic biopsies are painless and heal quickly within 3 days. On extremely rare occasions, the endoscopic procedure or biopsies may lead to pain, infection (sepsis), bleeding or perforation of the gastrointestinal tract. Perforation occurs approximately once out of every 1,000 procedures. If this extremely rare complication occurs, antibiotics and surgery to repair the tear may be necessary.

Rectal Swabs and Sponges
There is the risk of mild discomfort from both these procedures in addition to a slight risk of bleeding.

Enemas
The main risk from having an enema is temporary discomfort. A hollow tube about the thickness of a pencil will be used to put approximately 120 mL of Normosol-R pH 7.4 into the rectum and flush it out again (a larger volume may be required if the initial volume does not produce results), along with any stool that is there. This may cause a "bloated" or "cramping" feeling. Some air may be pumped into the rectum as well, causing flatulence. The tube is small, but it might cause some anal or rectal discomfort if the subject has any hemorrhoids or other painful conditions.

Applicator
Use of a vaginal applicator to deliver a vaginal microbicide into the rectal compartment may be associated with minor anorectal trauma including lacerations and bruising in the anorectal area.

Tenofovir 1% Gel
There is currently no rectal safety data regarding the use of tenofovir 1% gel. Administration of tenofovir gel intravaginally at 0.3% and 1% concentrations in the HPTN 050 Phase 1 study resulted in minimal local irritation and little or no systemic adverse effects were identified.[76] Although 92% of participants reported at least 1 AE, 87% of those reported AEs were mild, and 70% of the AEs were limited to the genitourinary tract. Four severe AEs were reported, with only one, lower abdominal pain, thought to be product-related. The risks associated with tenofovir gel are believed to be less than those identified for systemic use. In the HPTN 050 Phase 1 study of tenofovir gel, serum PK analysis in a subset of participants demonstrated that there is no clinically significant systemic toxicity. Fourteen of 24 women with PK results had low, but detectable, serum tenofovir levels.

Given that Phase 1 data demonstrates measurable plasma concentrations of tenofovir in some participants, participants with hepatitis B infection might be at risk for development of tenofovir resistant hepatitis B. However, participants with known hepatitis B infection will not be eligible for enrollment. It is not known what effect tenofovir gel could have on the HIV virus or HIV disease progression in HIV infected participants or their partners. There is a theoretical risk that tenofovir absorbed systemically from vaginal tenofovir gel could result in mutations of the HIV virus in participants who become infected with HIV during the study, or their partner, if the

partner is infected with HIV. Limited resistance data from HPTN 050 show no new resistance mutations in plasma or cervicovaginal lavage specimens after 14 days of tenofovir gel use. No participant had high level tenofovir mutations (e.g., K65R).

Some of the possible side effects of the study gel are dryness, itching, burning, or pain in the genital area.

In the male tolerance study of tenofovir 1% gel, there were few genital findings observed after product use and all findings were classified as mild, were small in size and required no treatment.[78] The most common symptoms included mild pain (burning, irritation, discomfort) and pruritus. All reported urogenital symptoms were felt to be mild.

2% Nonoxynol-9 Gel

As evidenced by several safety and dose escalation studies involving vaginally administered N-9[81, 82, 84, 86], N-9 oftentimes resulted in greater reports of genital irritation and evidence of vaginal inflammation. In light of evidence that N-9 can possibly increase the risk of HIV acquisition, participants will not only be informed about the possible side-effects of N-9, they will be counseled on the importance of remaining RAI abstinent throughout the course of the study.

While N-9 is demonstrated to cause acute and rapidly healing mucosal injury such as epithelial separation from underlying basement membrane, only one study has addressed participant symptom perception during N-9 use. In this safety and toxicity study of rectal application of N-9, participants reported symptoms of rectal fullness that were often mild and transient. The majority of receptive partners in this study reported feelings of anorectal discomfort characterized by burning, itching, and pain. Rectal bleeding was reported by 29% of the receptive participants.[14] Urinary or penile symptoms in insertive partners were reported by 18 (51%) insertive partners during 6 weeks of product use and by four (12%) participants during the week of placebo use. Genital examination identified a meatal ulceration in an HIV-negative participant 3 days after once-daily N-9 gel use. The ulceration was HSV culture negative and resolved within 3 days off gel.

Symptoms were commonly reported during N-9 and placebo use. Relatively more symptoms were reported during N-9 gel use and at increased frequencies of N-9 gel use even after taking into account that N-9 gel was applied for a longer period of time (6 weeks) than placebo gel (1 week). However, when compared to the brief exposure to placebo in the first week, no specific symptom, stratified by HIV status, was more common during the weeks of N-9 use. Despite these symptoms, study retention was extremely high and 98% of participants completed the 6-week study.

Placebo Gel

There is currently no rectal safety data regarding the use of placebo gel. Twice daily intravaginal administration of placebo gel over the course of two weeks resulted in mild genital irritation, including genital burning, soreness, and pelvic pain, in 2 out of 14

women (14.3%).[48,88] Three out of the 14 women (12.4%) had colposcopic findings which included erythema, petechiae and peeling, although no findings with deep disruption were observed during follow-up. The placebo gel did not appear to alter vaginal health or shift vaginal flora and no SAEs were reported.

13.3.2 Benefits

Participants in this study may experience no direct benefit. Participants and others may benefit in the future from information learned from this study. Specifically, information learned in this study may lead to the development of safe and effective interventions to prevent HIV transmission. Participants also may appreciate the benefit of earlier diagnosis of STIs in addition to the opportunity to contribute to the field of HIV prevention research. Additionally, participants will be referred for treatment for any incidental findings detected during screening and other examinations.

13.4 Informed Consent Process

Written informed consent will be obtained from each study participant prior to both screening and enrollment. Written informed consent also will be obtained for long-term specimen storage and possible future testing, although consent for specimen storage is not required for study participation. In obtaining and documenting informed consent, the investigators and their designees will comply with applicable local and US regulatory requirements and will adhere to GCP and to the ethical principles that have their origin in the Declaration of Helsinki. Study staff must document the informed consent process in accordance with the Requirements for Source Documentation in DAIDS Funded and/or Sponsored Clinical Trials available at:
(http://www3.niaid.nih.gov/research/resources/DAIDSClinRsrch/PDF/SourceDocPolicy.pdf).

Participants will be provided with copies of the informed consent forms if they are willing to receive them. Each study site is responsible for developing study informed consent forms for local use, based on the templates in the Appendices that describe the purpose of screening and of the study, the procedures to be followed, and the risks and benefits of participation, in accordance with all applicable regulations.

The informed consent process will cover all elements of informed consent required by research regulations. In addition, the process specifically will address the following topics of import to this study:

- The unknown safety and unproven efficacy of the study products
- The need to practice safer sex behaviors regardless of study treatment group
- The importance of participants in all four study groups to the success of the study
- The importance of adherence to the study visit and procedures schedule
- The potential medical risks of study participation (and what to do if such risks are experienced)

- The potential social harms associated with study participation (and what to do if such harms are experienced)
- The real yet limited benefits of study participation
- The distinction between research and clinical care
- The right to withdraw from the study at any time

The informed consent process will include an assessment of each potential participant's understanding prior to enrollment and randomization of concepts identified by the protocol team as essential to the informed consent decision. Participants who are not able to demonstrate adequate understanding of key concepts after exhaustive educational efforts will not be enrolled in the study.

13.5 Participant Confidentiality

All study procedures will be conducted in private, and every effort will be made to protect participant privacy and confidentiality to the extent possible. Each study site will implement confidentiality protections that reflect the local study implementation plan and the input of study staff and community representatives to identify potential confidentiality issues and strategies to address them. In addition to local considerations, the protections described below will be implemented at all sites.

All study-related information will be stored securely at the study site. All participant information will be stored in locked file cabinets in areas with access limited to study staff. Data collection, process, and administrative forms, laboratory specimens, and other reports will be identified by a coded number only to maintain participant confidentiality. All local databases will be secured with password-protected access systems. Forms, lists, logbooks, appointment books, and any other listings that link participant ID numbers to other identifying information will be stored in a separate, locked file in an area with limited access. Participants' study information will not be released without their written permission, except as necessary for review, monitoring, and/or auditing by:

- Representatives of the US Federal Government, including the US Food and Drug Administration (FDA), the US Office for Human Research Protections (OHRP), NIH, and/or contractors of the NIH
- Representatives of CONRAD
- Representatives of the MTN CORE, SDMC, and NL
- Site IRBs

13.6 Special Populations

This section outlines considerations made for the inclusion or exclusion of special populations in this study.

13.6.1 Pregnant Women

Women who test positive for pregnancy at screening or enrollment visits will not be eligible to participate in this study.

A urine pregnancy test will be performed on all women at all clinic visits, and additionally if clinically indicated; investigators will discontinue study product among participants who test positive for pregnancy. During the informed consent process, women will be informed that none of the study products are methods of contraception and that the effects of these products on a developing human fetus are unknown.

13.6.2 Children

The NIH has mandated that children be included in research trials when appropriate. This study meets "Justifications for Exclusion" criteria for younger children as set forth by the NIH. Specifically, "insufficient data are available in adults to judge potential risk in children" and "children should not be the initial group to be involved in research studies." This study does not plan to enroll children under 18 years old.

13.7 Compensation

Pending IRB approval, participants will be compensated for their time and effort in this study, and/or be reimbursed for travel to study visits, child care, and time away from work.

13.8 Communicable Disease Reporting

Study staff will comply with all applicable local requirements to report communicable diseases including HIV identified among study participants to local health authorities. Participants will be made aware of all reporting requirements during the study informed consent process.

13.9 Access to HIV-related Care

13.9.1 HIV Counseling and Testing

HIV test-related counseling will be provided to all potential study participants who consent to undergo HIV screening to determine their eligibility for this study, and to all enrolled participants at each follow-up HIV testing time point. Testing will be performed in accordance with the algorithm in Appendix II. Counseling will be provided in accordance with standard HIV counseling policies and methods at each site and additionally will emphasize the unknown efficacy of the study products in preventing HIV infection. In accordance with the policies of the US NIH, participants must receive their HIV test results to take part in this study.

13.9.2 Care for Participants Identified as HIV-Infected

Participants will be provided with their HIV test results in the context of post-test counseling. Participants found to be HIV-infected will be referred to available sources of medical and psychosocial care and support, and local research studies for HIV-infected adults.

13.10 Study Discontinuation

This study may be discontinued at any time by NIAID, the MTN, CONRAD, the US FDA, the OHRP, or site IRBs.

14 PUBLICATION POLICY

DAIDS/NIAID and MTN policies and a Clinical Trial Agreement (CTA) between CONRAD and NIAID will govern publication of the results of this study. Any presentation, abstract, or manuscript will be submitted by the Investigator to the MTN Manuscript Review Committee, DAIDS, and CONRAD, for review prior to submission.

15 APPENDICES

APPENDIX I: SCHEDULE OF STUDY VISITS AND EVALUATIONS

	SCR	ENR	TRTMT 1	F/U PHONE CALL	TRTMT 2	FINAL	F/U PHONE CALL	EARLY TERM.	INTERIM
Informed consents	X	X							
PTID	X								
Demographics	X								
Locator information	X	X	X		X	X		X	X
Test results		X	▲		▲	▲		▲	▲
Eligibility assessment/confirmation	X	X							
Randomization		X							
Reimbursement	X	X	X		X	X		X	
Schedule next study visit	▲	X	X		X				▲
Schedule follow-up phone assessment			X			X		▲	
Medical history	X	X	X		X	X		X	X
Menstrual history	♀	♀	♀		♀	♀		♀	♀
Concomitant meds	X	X	X		X	X		X	X
Physical exam	X	X	X		▲	X		X	▲
Rectal exam	X	X	X		▲	X		X	▲
Treat for RTIs/STIs/UTIs	▲	▲	▲		▲	▲		▲	▲
Document pre-existing conditions	X	X							
HIV pre-and-post test couns	X	▲				X		X	▲
HIV/STI risk reduction couns	X	X	X		X	X		X	X
Contraceptive couns	X	X	X		X				
Adherence couns (protocol and/or product use)		X	X		X				
Record/update AEs			X	X	X	X	X	X	▲
Baseline behavioral instructions and quest.		X							
Phone reporting system instructions*					X				
Product acceptability quest.*						X		X	
Qualitative hCG	♀	♀	♀		♀	♀		♀	♀
Dipstick UA	X					X		X	▲
Urine GC/CT by NAAT	X	▲	▲		▲	▲		▲	▲
CBC	X					X		X	▲
BUN, creatinine, ALT, AST	X					X		X	▲
Calculate creatinine clearance	X								
Syphilis RPR (confirm if necessary)	X	▲				X		X	▲
HIV-1 serology (confirm if necessary)	X	▲				X		X	▲
Plasma archive		X							
HBsAG	X								
HSV serology	X								
Rectal GC/CT by NAAT	X	▲	▲		▲	▲		▲	▲
Rectal microflora		X	X			X			
Rectal cytokines		X	X			X			
Normosol-R enema		X	X			X			
Rectal lavage,effluent, sloughing, and fecal calprotectin		X	X			X			
Anoscopy and biopsies		X	X		▲	X		▲	▲
Flex. sigmoidoscopy and biopsies		X	X			X		▲	▲
Condoms	X	X	X		X	X		X	X
Study gel and offer lubricant *			X		X				
Collect used & unused product*						X		X	▲

▲ If indicated; ♀ for females of childbearing potential; *for participants in treatment arms

APPENDIX II: HIV ANTIBODY TESTING ALGORITHM

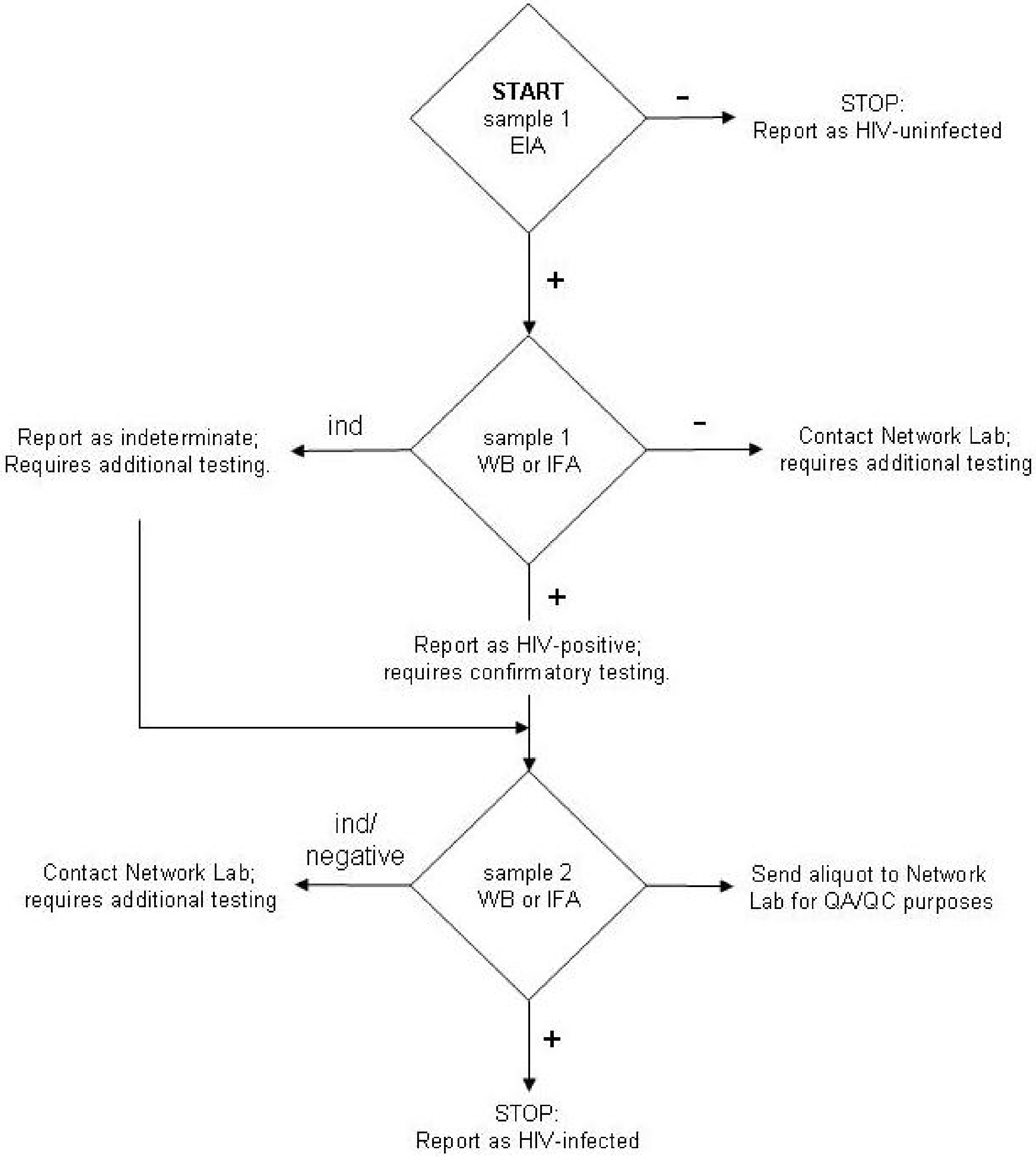

APPENDIX III: TOXICITY TABLES

The AE Grading Table Version 1.0, December 2004 (Clarification dated August 2009), Addendum 3 (Rectal Grading Table for Use in Microbicide Studies) will be the primary tool for grading adverse events for this protocol. Adverse events not included in that table will be graded by the DAIDS AE Grading Table Version 1.0, December 2004 (Clarification dated August 2009) or the DAIDS AE Grading Table Version 1.0, December 2004 (Clarification dated August 2009), Addendum 1 (Female Genital Grading Table for Use in Microbicide Studies). In cases where an AE is covered in all three tables, Addendum 3 (Rectal Grading Table for Use in Microbicide Studies) will be the grading scale utilized.

The Division of AIDS Table for Grading the Severity of Adult and Pediatric Adverse Events (DAIDS AE Grading Table), Version 1.0, December, 2004 (Clarification dated August 2009), is available on the RSC website at http://rsc.tech-res.com/safetyandpharmacovigilance/.

APPENDIX IV: MANUAL FOR EXPEDITED REPORTING OF ADVERSE EVENTS TO DAIDS

The current Manual for Expedited Reporting of Adverse Events to DAIDS is available at: http://rsc.tech-res.com/safetyandpharmacovigilance/.

APPENDIX V: HISTOPATHOLOGY SCORING SYSTEM

Participant ID: _________ **Visit No.: _______** **Visit Date: ______**

Biopsy Site: _______

Please Circle the Grade	Please Circle Subgrade	
**Grade 0**<br>No abnormality		
**Grade 1**<br>Mononuclear cell infiltrate	Low	High
**Grade 2**<br>Neutrophilic infiltrate lamina propria	Low	High
**Grade 3**<br>Neutrophilic infiltrate epithelium	Low<br>*< 50% of crypts*	High<br>*> 50% of crypts*
**Grade 4**<br>Crypt destruction	Low<br>*probable*	High<br>*unequivocal*
**Grade 5**<br>Erosion or ulceration	Low<br>*Restitution, probable erosion*	High<br>*Unequivocal erosion or ulceration*

APPENDIX VI: SAMPLE INFORMED CONSENT FORM (SCREENING)

SAMPLE INFORMED CONSENT FORM
DIVISION OF AIDS, NIAID, NIMH, US NIH

MTN-007
A Phase 1 Randomized, Double-Blinded, Placebo-Controlled Rectal Safety and Acceptability Study of Tenofovir 1% Gel

Version 2.0
August 13, 2010

PRINCIPAL INVESTIGATOR: [insert name]
PHONE: [insert number]
Short Title for the Study: Tenofovir Rectal Safety Study

INTRODUCTION

You are being asked to take part in these screening exams and tests for this research study because you are at least 18 years old, have had at least one experience of receptive anal sex in the past twelve months. This Microbicide Trials Network (MTN) study is sponsored by the US National Institutes of Health (NIH) and CONRAD. The person in charge of the study at this site is [INSERT NAME OF PRINCIPAL INVESTIGATOR]. The screening includes interview questions, urine and blood tests, a physical exam, and an examination of your rectum.

YOUR PARTICIPATION IS VOLUNTARY

This consent form gives information about the screening tests that will be discussed with you. Once you understand the screening tests, and if you agree to take part, you will be asked to sign your name on this form. You will be offered a copy of this form to keep.

Before you learn about the screening tests, it is important that you know the following:

- You do not have to be in this study if you do not want to
- You may decide not to have the screening tests, or you may withdraw from the screening tests at anytime
- You are only being asked at this time to have the screening tests. Even if you agree to have the screening tests, you do not have to join the research study
- Some people may not be able to join the research study because of information found during the screening tests
- You will receive the results of the screening tests even if you are not eligible to join the study

WHY ARE THE SCREENING EXAMS AND TESTS BEING DONE?
These exams and tests are being done to see if you can join this study.

WHY IS THIS STUDY BEING DONE?
There are four main purposes of this study. The first is to find out whether the main study product, tenofovir gel, is safe when inserted into the rectum. We would also like to see if the other two gels used in this study are also safe when inserted into the rectum. Another main purpose of this study is to find out how men and women feel about inserting tenofovir gel into their rectum. Other studies are being done to see if the tenofovir gel, when inserted into the vagina, can be used to prevent the spread of Human Immunodeficiency Virus, or HIV. HIV is the virus that causes Acquired Immunodeficiency Syndrome, or AIDS. Tenofovir gel is “experimental” for HIV prevention. This means **we do not know if it works to protect against HIV.** In future studies, we would like to see if tenofovir gel, when inserted into the rectum, can prevent the spread of HIV. In order to do that, we need to make sure that tenofovir is safe for use in the rectum, and understand how men and women feel about using the gel.

The side effects from tenofovir gel will be compared to the side effects of two other products: nonoxynol-9 (N-9) gel and placebo gel, as well as to participants who do not receive any of the study gels. N-9 gel is known to cause certain side effects and we want to make sure tenofovir gel does not cause the same side effects. The placebo gel does not have any medicine in it. Tenofovir gel and the placebo gel are not FDA approved for use in the rectum, and tenofovir gel has not been tested for rectal use in humans.

WHAT DO I HAVE TO DO IF I TAKE PART IN THE SCREENING EXAMS AND TESTS?
The screening visit will take about [sites to insert] hours. You will be asked to do the following things if you decide you want to join the study:

- Sign this form after you have read it or had it explained to you and had the chance to ask questions about the study
- Answer questions about yourself, such as where you live, your education, your behavior, including your sexual behavior, your medical history and any medicines that you may take and how we can contact you. Women will also be asked about menstrual cycle history
- Have a physical exam
- Have a rectal exam
- Learn about
    - different ways for women to avoid getting pregnant
    - how to avoid infections passed during sex
    - the meaning of your test results, including your HIV test results
    - how to use condoms
- Get treatment for any infections passed during sex or urinary tract infection that you may have, or learn from the study staff where you can get care or treatment

- Provide a urine sample to be tested for pregnancy (females) and urinary tract and sexually transmitted infections
- Have samples of fluid taken from your rectum to be tested for gonorrhea, chlamydia, and herpes
- Have a blood sample [sites to insert amount] taken to check the following:
    - the health of your blood, liver, and kidneys
    - HIV status
    - Syphilis status
    - Herpes status
- Receive condoms from the study staff
- It will take about [INSERT LENGTH OF TIME] to get the results of your screening tests. We will give you the test results when they are available.
- If the results of your screening tests and answers to the screening questions show you are able to take part in this study, the study staff will schedule an enrollment visit

WHY WOULD THE DOCTOR STOP THE SCREENING PROCEDURES EARLY?

The study doctor may need to stop the screening exams and tests early without your permission if:

- The study is cancelled by the US Food and Drug Administration (FDA), US National Institutes of Health (NIH), the MTN, the drug company supporting this study, the Office for Human Research Protections, the local government or regulatory agency, or the Institutional Review Board (IRB). An IRB is a committee that watches over the safety and rights of research participants
- Your exams, tests and answers to the questions show you cannot join the study
- Study staff believes that having the screening exams and tests would be harmful to you
- You do not want to learn your HIV test result
- You are not able to come to the visits or complete the screening exams and tests
- Other reasons that may prevent you from completing the study

WHAT ARE THE RISKS OF THE SCREENING VISIT TESTS?

Risk of Blood Draws:

- You may feel discomfort or pain when your blood is drawn
- You may feel dizzy, faint or lightheaded
- You may have a bruise, swelling, or infection where the needle goes into your arm

Risk of Rectal Exams:

- You may feel discomfort or pressure when your rectum is examined

Other Possible Risks:

- You may become embarrassed, worried, or nervous when discussing personal questions about your sexual behavior, ways to protect yourself against HIV and other infections passed during sex, and your test results

- You may become worried or nervous while waiting for your test results
- If you have HIV or other infections, knowing this could make you worried or nervous. A trained counselor will help you deal with any feelings or questions you may have

We will make every effort to protect your privacy during the screening exams and tests. Your visits will take place in private. However, it is possible that others may learn that you are taking part in the study here. Because of this, they may treat you unfairly.

ARE THERE BENEFITS TO TAKING PART IN THIS STUDY?

You may get no direct benefit from the screening exams and tests. However, you may benefit from the following:

- Physical exam and rectal exam
- Tests for sexually transmitted infections, rectal infections, and HIV (which may detect infections without obvious symptoms). If you have any of these infections, you will be referred for treatment if needed
- Tests to check your general health and the health of your liver, kidneys, and blood. This study cannot provide you with medical care, but study staff will refer you to other available sources of care
- Counseling regarding safer sex and free condoms
- If your tests show you are infected with HIV, you will be referred for medical care, counseling, and other services available to you. Medical care for HIV infection will not be part of this study. You will need to seek medical care for your HIV infection from your own health care provider or we will provide you with a referral to a center where you can receive care

WHAT OTHER CHOICES DO I HAVE BESIDES THIS STUDY?

You do not have to participate in this study, if you choose not to do so. [SITES TO INCLUDE/AMEND THE FOLLOWING IF APPLICABLE]: There may be other studies going on here or in the community for which you may be eligible. If you wish, we will inform you about other studies that are being conducted locally. There also may be other places where you can go for HIV counseling and testing. We will tell you about those places if you wish.] Please talk with your doctor about these and other choices that may be available to you. The decision to not be in this study will not affect your care in any way.

WHAT ABOUT CONFIDENTIALITY?

This study is being conducted according to ethical guidelines. Efforts will be made to keep your personal information private. Your physical and rectal exams will be done in private. We cannot guarantee absolute confidentiality. In some situations, including emergencies, legal and professional rules may force us to share confidential information about you. If this study is published, your name will not be used and you will not be personally identified. You are encouraged but not required to tell sexual partners about your participation in this study.

Your records may be reviewed by:

- Representatives of the US Federal Government, including the US Food and Drug Administration (FDA), the US Office for Human Research Protections (OHRP), NIH, and/or contractors of the NIH
- [INSERT NAME OF SITE] IRB
- Study staff
- CONRAD (the company that supplies the gel used in this study)

[SITES TO INCLUDE/AMEND THE FOLLOWING IF APPLICABLE:]
[LOCAL/STATE/NATIONAL] regulations require study staff to report the names of those who test positive for HIV and other infections passed during sex to the [LOCAL HEALTH AUTHORITY]. Outreach workers from the [HEALTH AUTHORITY] may then contact you about informing your partners, since they should also be tested. If you do not want to inform your partners yourself, the outreach workers will contact them, according to the confidentiality guidelines of the [HEALTH AUTHORITY].

In addition to the efforts made by the study staff to keep your personal information confidential, a Certificate of Confidentiality has been obtained from the US Federal Government for this study. This Certificate protects study staff from being forced to tell people who are not connected with this study, such as the court system, about your participation or information you provide for study purposes. Even with the Certificate of Confidentiality, however, if study staff learn of possible child abuse and/or neglect or a risk of harm to you or others, they will be required to tell the proper authorities. Having a Certificate of Confidentiality does not prevent you from releasing information about yourself and your participation in the study.

WHAT ARE THE COSTS TO ME?
There is no cost to you for the screening exams and tests.

WILL I RECEIVE ANY PAYMENT?
You will be paid for your time and effort for the screening visit. You will receive [SITES TO INSERT - SPECIFIC AMOUNT OF MONEY] for the visit. You will also be paid for other costs to you for coming to the screening visit [SUCH AS CHILD CARE, TRAVEL, AND LOSS OF WORK TIME – SITES TO COMPLETE].

WHAT HAPPENS IF I AM INJURED (EXPERIENCE HARM)?
If you are injured as a result of being in this study, the [INSTITUTION] will give you immediate treatment for your injuries. You [will/will not] have to pay for this treatment. You will be told where you can get additional treatment for your injuries. The US National Institutes of Health (NIH) does not have a program to provide money or other forms of compensation for your injuries. Signing this consent form does not change your legal rights.

[SITES TO SPECIFY INSTITUTIONAL POLICY]

WHAT ARE MY RIGHTS AS A RESEARCH PARTICIPANT?

Taking part in the screening exams and tests is completely voluntary. You may choose not to have the screening exams and tests any time. You will be treated the same no matter what you decide.

We will tell you about new information from this or other studies that may affect your health, welfare or willingness to stay in this study. If you want the results of the study, please inform let study staff.

WHAT DO I DO IF I HAVE PROBLEMS OR QUESTIONS?

For questions about the screening exams and tests or if you have a research-related injury, you should contact:

- [SITE INSERT NAME OF THE INVESTIGATOR OR OTHER STUDY STAFF]
- [SITE INSERT TELEPHONE NUMBER AND PHYSICAL ADDRESS OF ABOVE]

For questions about your rights as a research participant, contact:

- [SITE INSERT NAME OR TITLE OF PERSON ON THE INSTITUTIONAL REVIEW BOARD (IRB) OR OTHER ORGANIZATION APPROPRIATE FOR THE SITE]
- [SITE INSERT TELEPHONE NUMBER AND PHYSICAL ADDRESS OF ABOVE]

SIGNATURES
[INSERT SIGNATURE BLOCKS AS REQUIRED BY LOCAL IRB]

If you have read the informed consent (or had it read and explained to you), and all your questions have been answered, please sign your name below.

Participant Name (print)	Participant Signature	Date
Study Staff Conducting Consent Discussion (print)	Study Staff Signature	Date

APPENDIX VII: SAMPLE INFORMED CONSENT DOCUMENT (ENROLLMENT)

SAMPLE INFORMED CONSENT FORM
DIVISION OF AIDS, NIAID, NIMH, US NIH

MTN-007
A Phase I Randomized, Double-Blinded, Placebo-Controlled Rectal Safety and Acceptability Study of Tenofovir 1% Gel

Version 2.0
August 13, 2010

PRINCIPAL INVESTIGATOR: [insert name]
PHONE: [insert number]
Short Title for the Study: Tenofovir Rectal Safety Study

INTRODUCTION

You are being asked to take part in these enrollment exams and tests for this research study because you are at least 18 years old, reported at least one experience of receptive anal sex in the past twelve months at the Screening Visit, and have passed the screening for this research study. This Microbicide Trials Network (MTN) study is sponsored by the US National Institutes of Health (NIH) and CONRAD. The person in charge of this study at this site is [INSERT NAME OF PRINCIPAL INVESTIGATOR]. Before you decide if you want to join this study, we want you to know about this study.

YOUR PARTICIPATION IS VOLUNTARY

This is an enrollment consent form and gives you information about the study. Study staff will talk with you about this information. You are encouraged to ask questions about the study at any time. If you agree to take part in this study, you will be asked to sign your name on this form. You will be offered a copy of this form to keep.

Before you learn about this study, it is important that you know the following:

- You do not have to join this study if you do not want to do so
- You may decide not to have the enrollment tests or you may withdraw from the enrollment tests at anytime
- Some people may not be able to join the research study because of information found during the enrollment process
- You will receive the results of your tests even if you are not eligible to join the research study

WHY IS THIS STUDY BEING DONE?

There are four main purposes of this study. The first is to find out whether the main study product, tenofovir gel, is safe when inserted into the rectum. We would also like

to see if the other two gels used in this study are also safe when inserted into the rectum. Another main purpose of this study is to find out how men and women feel about inserting tenofovir gel into their rectum. Other studies are being done to see if the tenofovir gel, when inserted into the vagina, can be used to prevent the spread of Human Immunodeficiency Virus, or HIV. HIV is the virus that causes Acquired Immunodeficiency Syndrome, or AIDS. Tenofovir gel is "experimental" for HIV prevention. This means **we do not know if it works to protect against HIV.** In future studies, we would like to see if tenofovir gel, when inserted into the rectum, can prevent the spread of HIV. In order to do this, we need to make sure that tenofovir is safe for use in the rectum, and understand how men and women feel about using it.

The side effects from tenofovir gel will be compared to the side effects from two other products: nonoxynol-9 (N-9) gel and placebo gel as well as to the participants who do not receive any of the study gels. N-9 gel is known to cause certain side effects and we would like to make sure that tenofovir gel does not cause the same side effects as N-9. The placebo gel does not have any medicine in it. Tenofovir gel and the placebo gel are not FDA approved for use in the rectum, and tenofovir gel has not been tested for rectal use in humans.

STUDY GROUPS

There are four study groups. If you decide to take part in the study, you will be placed in one of the four groups: tenofovir gel, N-9 gel, placebo gel OR no gel. You will have a 1 in 4 chance of being in any one study group. There will be 15 participants assigned to each group for a total of 60 participants in the whole study. There are two different treatment periods for this study. At the first treatment period, if you are in one of the groups that receives a study gel, you will receive one dose of the study gel at that clinic visit. The second treatment period begins one week later. At that clinic visit, you will be given a 7-day supply of the same study gel as you received in your first treatment period. Your group will be chosen "by lot" [or other equivalent local term, for example, like flipping a coin or throwing dice] to be in one of these groups. You cannot choose your group, and the study staff cannot choose your group for you. Once you are in a group, you cannot change to another group. The study procedures will be the same for everyone participating in the study. The study staff and study doctor will not know what group you are in. All four of these groups are very important to the results of the study.

WHAT DO I HAVE TO DO IF I AM IN THIS STUDY?

If you decide to take part in this study, your first visit will continue today, after you read, discuss, understand, and sign this form. Study staff will help you understand the form and answer your questions before you sign this form.

Today, if you decide to participate and sign this form, you will find out which group you are in. You will answer questions about your sexual practices and will answer some questions about your medical history to make sure you are still eligible to join this study. Women will also be asked about menstrual cycle history. At today's visit, you will also:

- Have a blood sample [insert amount] taken in case there is a question about your lab results. After all testing is done, this sample will be destroyed according to the sites procedure for getting rid of blood samples that will not be needed after the end of the study
- Be asked to complete the informed consent document for the storage and future testing of specimens. You will only be asked to sign a separate consent document if you give your permission for the study staff to store your specimens for future testing

You will be in the study for about 4 to 11 weeks from the time of your Enrollment Visit (today) up until your follow-up phone call at the end of the study, and will use the study gel for a total of 8 days. Most of the visits will take [insert approximate amount of time]. Visits where study doctors will take small samples of tissue from your rectum will take [insert approximate amount of time].

At most visits, we will ask you to do the following:

- Let us know if there are any changes in where you live or how we may contact you
- Tell us about any changes in your medical or menstrual history
- Tell us if there have been changes to any medicines you are taking now
- Receive test results from previous visit, if available
- Have a physical exam
- Have an exam of your rectum
- Learn how to follow the rules of the study
- Learn about:
    - How to avoid infections that may be passed during sex, including HIV
    - How to avoid becoming pregnant
- If you are female, have your urine tested for pregnancy
- Receive condoms from the study staff

In addition, at the Enrollment, Treatment 1, and Final Visits, you will:

- Have an enema, which is a liquid injected into your rectum to promote a bowel movement. The liquid collected from your rectum afterwards will be tested to check the health of your rectum
- Have an examination of your rectum called anoscopy. This is when a short, hollow tube is placed inside your rectum to allow the study doctor to take a sample of rectal tissue
    - The doctor will take small tissue samples from near the opening of your rectum (biopsy). The tissue samples will be tested to check the health of your rectum
- Have an examination of your rectum (flexible sigmoidoscopy). This is when a flexible, long (a little longer than the anoscope) hollow tube is placed inside your rectum so that the study doctor can check the health of your rectum and take a sample of rectal tissue

    - The doctor will take small tissue samples (biopsy) from further inside your rectum. The tissue samples will be tested to check the health of your rectum

Follow-Up Phone Call

This phone call will take place a day after the Treatment 1 Visit and will take about [Insert amount of time]. During this phone call, we will ask you to:

- Tell us about any side-effects you might have had from using the study gel

At the Treatment 1, Treatment 2 and Final Visits, you will:

- Be checked to see if the gel or biopsies caused any side-effects

At the Treatment 1 and Treatment 2 Visits, if you are in the group that receives the study gel, you will:

- Self-administer one dose of the study gel under the observation of study staff. You will also receive study lubricant to make it easier to insert the applicator. (Treatment 1 Visit)
- Receive a 7-day supply of study gel and study lubricant (this will be the same study gel that you received at the first treatment visit). (Treatment 2 Visit)
- Call an automated phone system each time you use the gel at home. You should insert the gel as close to the same time as possible, before your longest period of rest. When you call, you will be asked a brief set of questions. You will learn how the phone system works, and about the compensation you will receive for the calls. You will also have the opportunity to try the phone system out and ask any questions you may have. You may also be contacted by the study staff if you miss a phone call. (Treatment 2 Visit)

Final Visit

- If you are in the group that receives the study gel, answer questions about your experience using the study gel, including what you did and did not like about the gel
- Provide a urine sample to get tested for urinary tract infections
- Have a blood sample [insert amount] taken to:
    - Check the health of your blood, liver, and kidneys
    - Check for syphilis
    - Check for HIV
- You will be asked to return all used and unused applicators to the study clinic
- Schedule your follow-up telephone call

Follow-Up Phone Call

This phone call will take about [INSERT AMOUNT OF TIME]. During this phone call, we will ask you to:

- Tell us about any side-effects you might have had from using the study gel, if you are in the group that receives the study gel

At Any Time In The Study

If the study doctor thinks you have health problems that may be caused by infections, including those passed through sex, or if you have signs of infections, including those passed through sex, you may:

- Have a physical or rectal exam
- Give blood, urine, rectal fluid, or rectal tissue samples to test for infections
- Provide a urine sample for a pregnancy test (women)
- Get treatment or referrals for most types of infections if you need it

ARE THERE ANY RISKS AND/OR DISCOMFORTS?

Risks from Phlebotomy (blood tests)

- You may feel discomfort or pain when your blood is drawn
- You may feel dizzy or faint
- You may have a bruise, swelling, small clot, or infection where the needle goes in your arm or finger

RISK OF RECTAL EXAMS

- You may feel discomfort or pressure when your rectum is examined
- You may experience some discomfort when the swab or sponge is inserted into the rectum, and occasionally minor rectal bleeding may occur

Risks from Anoscopy and Flexible Sigmoidoscopy with Biopsies

- You may experience some mild discomfort and feel like you have a "bloated stomach"
- Even though the risk is low, you may experience infection, mild rectal irritation and may feel a sudden urge to have a bowel movement
- You may experience limited rectal bleeding (1 to 2 days after the procedure) related to the biopsies
- You may experience low blood pressure
- Even though the risk is very rare, there is a very small chance that you may have a hole or a tear in the intestine. This only happens once out of every 1,000 procedures. If this were to happen, surgery to repair the tear may be necessary. It is important that you do not put anything in your rectum for 7 days after the biopsies, because you may be at higher risk for getting or spreading an infection until the biopsy site(s) have healed

Risks from Enemas

- You may experience some mild discomfort and a bloated or cramping feeling

Risks from the Applicator

- You may experience some discomfort from the applicator since it has been designed for vaginal rather than rectal use

Risks from Tenofovir Gel
If you are in the group receiving tenofovir gel, the gel could cause some bad effects, which are described below. We do not yet know all the bad effects of tenofovir gel and we do not know what effects tenofovir gel will have on the rectum. Some, but not all, women who used the tenofovir gel in other studies have had:

- Dryness, itching, burning feeling, or pain in the genital area
- Vaginal candidiasis (a kind of vaginal infection)
- Discharge from the vagina
- Irritation in the genital area

Risks from N-9
If you are in a group that gets N-9 gel, the gel could cause some bad effects, which are described below. Many studies have been done to study the effects of N-9 gel, and show that when N-9 is used rectally, it can cause temporary damage to the lining of the rectum. Some men and women who used N-9 rectally in other studies have had:

- Burning, itching, or pain in the rectum
- Diarrhea or loose stools
- Rectal bleeding
- Enhanced transmission of HIV and other STIs

Risks from Placebo Gel
If you are in a group that gets placebo gel, the gel could cause some bad effects, which are described below. Some studies have been done to study the effects of placebo gel, and show that when it used vaginally, it can cause the following:

- Burning or soreness in the genital area
- Pain in the pelvic area

Pregnancy/Risks to Fetus
The effects of the study gels on the pregnancy or on baby's development are not known. It is for this reason that study staff will counsel you on ways to avoid becoming pregnant while you are in the study.

Other Possible Risks:

- You may become embarrassed, worried, or nervous when discussing personal questions about your sexual behavior, ways to protect against HIV and other infections passed during sex, and your test results
- You may become worried or nervous while waiting for your test results
- If you have HIV or other infections, knowing this could make you worried or nervous. A trained counselor will help you deal with any feelings or questions you have

We will make every effort to protect your privacy while you are having the study visits, exams, and tests. Reports to the automated phone system will be stored in computers that are password-protected and will not include personal information that could identify or link information to you. Your visits here will take place in private. However, it is possible that others may learn that you are taking part in the study here. Because of

this, they may treat you unfairly or discriminate against you. For example, you could have problems getting or keeping a job, or being accepted by your family or community. Finding out your HIV status could also cause problems between you and your partner. Some studies of HIV prevention have found an unexpected higher risk of study participants contracting HIV. This could happen in any study, including this study. Because of this, the study staff will remind you not to have receptive anal sex while participating in this study, and will also remind you of the importance of using condoms to protect against HIV.

WHAT ARE THE BENEFITS?

You may get no direct benefit from being in this study. **We do not know if tenofovir gel works to protect against HIV.** Also, you may not receive any gel and if you do, the gel you receive may be the N-9 or the placebo gel. Because of this, study staff will remind you of the importance of not having receptive anal sex during the study and will also remind you of the importance of using condoms to protect against HIV.

You or others may benefit in the future from information learned in this study. You may also get some personal satisfaction from being part of research on HIV prevention. This is true no matter what study group you are in.

You will have physical exams and rectal exams. You will have tests to check on the health of your blood, liver, and kidneys. If these tests show that you might have any health problems, you will be referred for medical care and other services available to you.

You will get counseling and testing for HIV. You will receive free condoms. If you have infections passed through sex, other than HIV infection, you will be offered medicine to treat them, if needed. This study does not provide medication for treatment of HIV/AIDS. If you become infected with HIV, you will be referred for medical care, counseling, and other services available to you.

WHY MIGHT I HAVE TO STOP TAKING THE STUDY DRUG EARLY?

You may have to stop using gel if you:

- Become infected with HIV
- Become infected with hepatitis B
- Become pregnant
- Are breastfeeding
- Are taking certain medications that affect your kidneys
- Are unable or unwilling to follow study procedures or instructions
- Could be harmed by continuing to take gel

WHY MIGHT I BE WITHDRAWN FROM THE STUDY WITHOUT MY CONSENT?

You may be withdrawn from the study without your consent for the following reasons:

- The study is stopped or canceled
- Study staff feel that staying in the study would be harmful to you
- You are not willing to find out your HIV test results

- Other reasons, decided by the study staff

If you withdraw early from the study, we will ask you to come in for a final visit with all the exams and tests listed for the final visit, if the study doctor thinks the exams and tests need to be done.

WHAT OTHER CHOICES DO I HAVE BESIDES THIS STUDY?
You do not have to be in this study. The decision to not be in this study will not affect your care in any way

WHAT ARE THE COSTS TO ME?
There is no cost to you for the study procedures and exams.

WILL I RECEIVE ANY PAYMENT?
You will be paid for your time and effort for your scheduled study visits. You will receive [SITES TO INSERT - SPECIFIC AMOUNT OF MONEY] for each visit. You will also be paid for other costs to you for coming to your scheduled visits [SUCH AS CHILD CARE, TRAVEL, AND LOSS OF WORK TIME – SITES TO COMPLETE].

During the Final Visit you will receive $2 for each call you made to the phone reporting system ($2 per day X 7 days = $14), plus $10 if you make a call every day during the 7 days that you used the study gel, for a total of up to $24.

WHAT ABOUT CONFIDENTIALITY?
This study is being conducted according to ethical guidelines. Efforts will be made to keep your personal information private. Your physical and rectal exams will be done in private. We cannot guarantee absolute confidentiality. In some situations, including emergencies, legal and professional rules may force us to share confidential information about you. If this study is published, your name will not be used and you will not be personally identified. You are encouraged but not required to tell sexual partners about your participation in this study.

Your records may be reviewed by:
- Representatives of the US Federal Government, including the US Food and Drug Administration (FDA), the US Office for Human Research Protections (OHRP), NIH, and/or contractors of the NIH
- [INSERT NAME OF SITE] IRB
- Study staff
- CONRAD (the company that supplies the gels used in this study)

[SITES TO INCLUDE/AMEND THE FOLLOWING IF APPLICABLE:]
[LOCAL/STATE/NATIONAL] regulations require study staff to report the names of those who test positive for HIV and other infections passed during sex to the [LOCAL HEALTH AUTHORITY]. Outreach workers from the [HEALTH AUTHORITY] may then contact you about informing your partners, since they should also be tested. If you do

not want to inform your partners yourself, the outreach workers will contact them, according to the confidentiality guidelines of the [HEALTH AUTHORITY].

In addition to the efforts made by the study staff to keep your personal information confidential, a Certificate of Confidentiality has been obtained from the US Federal Government for this study. This Certificate protects study staff from being forced to tell people who are not connected with this study, such as the court system, about your participation or information you provide for study purposes. Even with the Certificate of Confidentiality, however, if study staff learn of possible child abuse and/or neglect or a risk of harm to you or others, they will be required to tell the proper authorities. Having a Certificate of Confidentiality does not prevent you from releasing information about yourself and your participation in the study.

WHAT HAPPENS IF I AM INJURED (EXPERIENCE HARM)?

[SITES TO SPECIFY INSTITUTIONAL POLICY] It is unlikely that you will be injured as a result of study participation. If you are injured, the [institution] will give you immediate necessary treatment for your injuries. You [will/will not] have to pay for this treatment. You will be told where you can get additional treatment for your injuries. There is no program to pay money or give other forms of compensation for such injuries. You do not give up any legal rights by signing this consent form.

WHAT ARE MY RIGHTS AS A RESEARCH PARTICIPANT?

Taking part in exams and tests is completely voluntary. You may choose not to have the exams and tests any time. You will be treated the same no matter what you decide.

We will tell you about new information from this or other studies that may affect your health, welfare or willingness to stay in this study. If you want the results of the study, please inform let study staff.

WHAT DO I DO IF I HAVE PROBLEMS OR QUESTIONS?

If you ever have any questions about the study, or if you have a research-related injury, you should contact [INSERT NAME OF THE INVESTIGATOR OR OTHER STUDY STAFF] at [INSERT TELEPHONE NUMBER AND/OR PHYSICAL ADDRESS].

If you have questions about your rights as a research participant, you should contact [insert name or title of person on the IRB or other organization appropriate for the site] at [insert physical address and telephone number].

If you have questions about whom to contact at the research site, you should contact [insert name of the investigator or community educator or community advisory board (CAB) member [staff will decide which] at [insert physical address and telephone number].

[SITES THAT PREFER NOT TO INCLUDE THE TABLE OF STUDY VISITS AND EVALUATIONS ON THE FOLLOWING PAGE MAY DELETE THIS ATTACHMENT]

SIGNATURE PAGE
[INSERT SIGNATURE BLOCKS AS REQUIRED BY LOCAL IRB]

If you have read the informed consent (or had it read and explained to you), and all your questions have been answered, please sign your name below.

______________________	______________________	
Participant Name (print)	Participant Signature	Date
Study Staff Conducting Consent Discussion (print)	Study Staff Signature	Date

MTN-007 Schedule of Study Visits and Evaluations for Participants

	Screening	Enrollment	Treatment 1	Follow-up phone call	Treatment 2	Final	Follow-up phone call	Early Termination Visit
Informed consents	X	X						
Study identification number	X							
Information about where you live and how we can contact you	X	X	X		X	X		X
Questions to see if you can join the study	X	X						
Test results		X	▲		▲	▲		▲
Information about the study	X							
Information about your health and the medicines you are taking	X	X	X		X	X		X
Physical exam	X	X	X		▲	X		X
Rectal exam	X	X	X		▲	X		X
Questions about your health after you start using the study gel			X	X	X	X	X	X
HIV pre-and post-test counseling	X	▲				X		X
Learn about ways to avoid pregnancy	X	X	X		X			
Learn about ways to avoid getting infections passed through sex	X	X	X		X	X		X
Learn how to follow the rules of the study		X	X		X			
Baseline behavioral questionnaire		X						
Learn about the phone reporting system					X			
Answer questions about the study gel						X		X
Receive study gel			X		X			
Return used and/or unused applicators to the clinic						X		X
**Laboratory Tests**								
HIV and syphilis tests	X	▲				X		X
Test for herpes	X							
Test for hepatitis	X							
Tests for gonorrhea and chlamydia	X	▲	▲		▲	▲		▲
Blood tests	X	X				X		X
Urine sample for pregnancy test	♀	♀	♀		♀	♀		♀
Urine samples to check the health of your liver and kidneys	X					X		X
Enema		X	X			X		
Flexible sigmoidoscopy, anoscopy, and rectal tissue samples		X	X		▲	X		▲

X = required, ▲= if indicated, ♀=required for female participants

APPENDIX VIII: SAMPLE INFORMED CONSENT DOCUMENT (Storage and Future Testing of Specimens)

SAMPLE INFORMED CONSENT FORM
DIVISION OF AIDS, NIAID, NIMH, US NIH

MTN-007
A Phase 1 Randomized, Double-Blinded, Placebo-Controlled Rectal Safety and Acceptability Study of Tenofovir 1% Gel

Version 2.0
August 13, 2010

PRINCIPAL INVESTIGATOR: [insert name]
PHONE: [insert number]
Short Title for the Study: Tenofovir Rectal Safety Study

INTRODUCTION

You have decided to take part in a Division of AIDS research study. While you are in this research study there may be some samples of tissue, and/or fluid from your rectum taken from you that might be useful for future research. You are being asked to agree to the storage of these samples. This consent form gives you information about the collection, storage and use of your samples. The study staff will talk with you about this information. Please ask any questions, if you have some. If you agree to the storage of your samples, you will be asked to sign or make your mark on this consent form. You will be given a copy of this form to keep.

HOW WILL YOU GET THE SAMPLES FROM ME?

The research doctors want to save any extra tissue and/or rectal fluid leftover from your tests during the study. The leftover tissue samples and rectal fluid will be kept and used for future research.

HOW WILL YOU USE MY SAMPLES?

Your samples will be used to look for ways that your body responds to infection (such as cells, proteins, and other chemicals in your body). Tests may also include checking your genes (material passed from parent to child that determines the make-up of the body and mind), since they might affect how your body responds to disease. Your genes might make you more or less likely to get an infection, affect your responses to infection, or make your responses to treatment stronger or weaker. No other kinds of genetic test will be done on your stored samples without first explaining the test to you and getting your permission. The researchers do not plan to contact you or your regular doctor with any results from tests done on your stored samples. This is because research tests are often done with experimental procedures, so the results from one

research study are generally not useful for your medical care. Your samples will not be sold or used directly to produce products that can be sold for profit.

Research studies using your samples will be reviewed by the National Institutes of Health and a special committee at the researcher's institution (an Institutional Review Board) whose purpose is to protect you as a research participant.

HOW LONG WILL YOU KEEP MY SAMPLES?
There is no time limit on how long your samples will be stored.

HOW WILL MY SAMPLES BE STORED?
Your samples will be stored at special facilities that are designed to store samples securely. The storage facilities are made so that only approved researchers will have access to the samples. An Institutional Review Board will oversee the storage facilities to protect you and other research volunteers from harm.

DOES STORAGE OF MY SAMPLES BENEFIT ME?
There are no direct benefits to you.

WHAT ARE THE RISKS?
There are few risks related to storing your samples. When tests are done on the stored samples there is a small but possible risk to your privacy. It is possible that if others found out information about you from tests (such as information about your genes) it could cause you problems with your family (having a family member learn about a disease that may be passed on in families or learning who is the biological parent of a child) or problems getting a job or insurance.

WHAT ABOUT CONFIDENTIALITY?
To keep your information private, your samples will be labeled with a code that can only be traced back to your research clinic. Your personal information (name, address, phone number) will be protected by the research clinic. When researchers are given your stored samples to study they will not be given your personal information. The results of future tests will not be included in your health records.

Your records may be reviewed by:

- Representatives of the US Federal Government, including the US Food and Drug Administration (FDA), the US Office for Human Research Protections (OHRP), NIH, and/or contractors of the NIH
- [INSERT NAME OF SITE] IRB
- Study staff
- CONRAD (the company that supplies the gels used in this study)

In addition to the efforts made by the study staff to keep your personal information confidential, a Certificate of Confidentiality has been obtained from the US Federal Government for this study. This Certificate protects study staff from being forced to tell people who are not connected with this study, such as the court system, about your

participation or information you provide for study purposes. Even with the Certificate of Confidentiality, however, if study staff learn of possible child abuse and/or neglect or a risk of harm to you or others, they will be required to tell the proper authorities. Having a Certificate of Confidentiality does not prevent you from releasing information about yourself and your participation in the study.

WHAT ARE MY RIGHTS?
Allowing your samples to be stored is completely voluntary. You may decide not to have any samples stored other than what is needed to complete this study and still be in this research study or any future study. If you decide now that your samples can be stored for future research, you may change your mind at any time. You must contact your study doctor or nurse and let them know that you do not want your samples used for future research. Your samples will then not be used and will be destroyed.

WHAT DO I DO IF I HAVE QUESTIONS?
For questions about the storage of your samples, contact (*insert the name of the investigator*) at *(insert telephone number)*.

For questions about your rights related to the storage of your samples for research, contact (*insert the name or title of person on the Institutional Review Board) at (insert telephone number)*.

SIGNATURE PAGE
[INSERT SIGNATURE BLOCKS AS REQUIRED BY LOCAL IRB]

Please carefully read the statements below and think about your choice. No matter what you decide it will not affect your participation in the MTN-007 study or your medical care. Please initial or mark your choice and sign your name below.

____I agree to allow my leftover samples to be stored for future testing.

OR

____I do not agree to allow my leftover samples to be stored for future testing.

Participant's Name (print)	Participant's Signature	Date
Study Staff Conducting Consent Discussion (print)	Study Staff Signature	Date

REFERENCES

1. Koblin BA, Chesney MA, Husnik MJ, et al. High-risk behaviors among men who have sex with men in 6 US cities: baseline data from the EXPLORE Study. Am J Public Health 2003;93:926-32.
2. Gross M, Holte SE, Marmor M, Mwatha A, Koblin BA, Mayer KH. Anal sex among HIV-seronegative women at high risk of HIV exposure. The HIVNET Vaccine Preparedness Study 2 Protocol Team. J Acquir Immune Defic Syndr 2000;24:393-8.
3. Civic D. College students' reasons for nonuse of condoms within dating relationships. J Sex Marital Ther 2000;26:95-105.
4. Mosher WD, Chandra A, Jones J. Sexual behavior and selected health measures: men and women 15-44 years of age, United States, 2002. Advance Data 2005:1-55.
5. Erickson PI, Bastani R, Maxwell AE, Marcus AC, Capell FJ, Yan KX. Prevalence of anal sex among heterosexuals in California and its relationship to other AIDS risk behaviors. AIDS Educ Prev 1995;7:477-93.
6. Poles MA, Elliott J, Taing P, Anton PA, Chen IS. A preponderance of CCR5(+) CXCR4(+) mononuclear cells enhances gastrointestinal mucosal susceptibility to human immunodeficiency virus type 1 infection. J Virol 2001;75:8390-9.
7. Leynaert B, Downs AM, de Vincenzi I. Heterosexual transmission of human immunodeficiency virus: variability of infectivity throughout the course of infection. European Study Group on Heterosexual Transmission of HIV. American Journal of Epidemiology 1998;148:88-96.
8. Vittinghoff E, Douglas J, Judson F, McKirnan D, MacQueen K, Buchbinder SP. Per-contact risk of human immunodeficiency virus transmission between male sexual partners. American Journal of Epidemiology 1999;150:306-11.
9. Gray RH, Wawer MJ, Brookmeyer R, et al. Probability of HIV-1 transmission per coital act in monogamous, heterosexual, HIV-1-discordant couples in Rakai, Uganda. Lancet 2001;357:1149-53.
10. Padian NS, Shiboski SC, Glass SO, Vittinghoff E. Heterosexual transmission of human immunodeficiency virus (HIV) in northern California: results from a ten-year study. American Journal of Epidemiology 1997;146:350-7.
11. McGowan I, Elliott J, Cortina G, et al. Characterization of baseline intestinal mucosal indices of injury and inflammation in men for use in rectal microbicide trials (HIV Prevention Trials Network-056). J Acquir Immune Defic Syndr 2007;46:417-25.
12. Phillips DM, Sudol KM, Taylor CL, Guichard L, Elsen R, Maguire RA. Lubricants containing N-9 may enhance rectal transmission of HIV and other STIs. Contraception 2004;70:107-10.
13. Phillips DM, Taylor CL, Zacharopoulos VR, Maguire RA. Nonoxynol-9 causes rapid exfoliation of sheets of rectal epithelium. Contraception 2000;62:149-54.
14. Tabet SR, Surawicz C, Horton S, et al. Safety and toxicity of nonoxynol-9 gel as a rectal microbicide. Sex Transm Dis 1999;26:564-71.
15. Anton PA. A phase I safety and acceptability study of the UC-781 microbicide gel applied rectally in HIV seronegative adults: an interim safety report at 50% completion. In: Microbicides 2008; 2008; New Delhi, India; 2008.
16. Patton DL, Cosgrove Sweeney YT, McCarthy TD, Hillier SL. Preclinical safety and efficacy assessments of dendrimer-based (SPL7013) microbicide gel formulations

in a nonhuman primate model. Antimicrobial Agents & Chemotherapy 2006;50:1696-700.
17. Patton DL, Sweeney YC, Cummings PK, Meyn L, Rabe LK, Hillier SL. Safety and efficacy evaluations for vaginal and rectal use of BufferGel in the macaque model. Sex Transm Dis 2004;31:290-6.
18. Patton DL, Sweeney YT, Balkus JE, Hillier SL. Vaginal and rectal topical microbicide development: safety and efficacy of 1.0% Savvy (C31G) in the pigtailed macaque. Sexually Transmitted Diseases 2006;33:691-5.
19. Geboes K, Riddell R, Ost A, Jensfelt B, Persson T, Lofberg R. A reproducible grading scale for histological assessment of inflammation in ulcerative colitis.[see comment]. Gut 2000;47:404-9.
20. Shacklett BL, Yang O, Hausner MA, et al. Optimization of methods to assess human mucosal T-cell responses to HIV infection. Journal of Immunological Methods 2003;279:17-31.
21. Anton PA, Elliott J, Poles MA, et al. Enhanced levels of functional HIV-1 co-receptors on human mucosal T cells demonstrated using intestinal biopsy tissue. AIDS 2000;14:1761-5.
22. Lapenta C, Boirivant M, Marini M, et al. Human intestinal lamina propria lymphocytes are naturally permissive to HIV-1 infection. European Journal of Immunology 1999;29:1202-8.
23. Patterson BK, Czerniewski M, Andersson J, et al. Regulation of CCR5 and CXCR4 expression by type 1 and type 2 cytokines: CCR5 expression is downregulated by IL-10 in CD4-positive lymphocytes. Clinical Immunology 1999;91:254-62.
24. Weissman D, Dybul M, Daucher MB, Davey RT, Jr., Walker RE, Kovacs JA. Interleukin-2 up-regulates expression of the human immunodeficiency virus fusion coreceptor CCR5 by CD4+ lymphocytes in vivo. Journal of Infectious Diseases 2000;181:933-8.
25. Brenchley JM, Hill BJ, Ambrozak DR, et al. T-cell subsets that harbor human immunodeficiency virus (HIV) in vivo: implications for HIV pathogenesis. Journal of Virology 2004;78:1160-8.
26. Brenchley JM, Price DA, Schacker TW, et al. Microbial translocation is a cause of systemic immune activation in chronic HIV infection.[see comment]. Nature Medicine 2006;12:1365-71.
27. Fichorova RN, Bajpai M, Chandra N, et al. Interleukin (IL)-1, IL-6, and IL-8 predict mucosal toxicity of vaginal microbicidal contraceptives. Biology of Reproduction 2004;71:761-9.
28. Fichorova RN, Richardson-Harman N, Alfano M, et al. Biological and technical variables affecting immunoassay recovery of cytokines from human serum and simulated vaginal fluid: a multicenter study. Analytical Chemistry 2008;80:4741-51.
29. McGowan I, Elliott J, Fuerst M, et al. Increased HIV-1 mucosal replication is associated with generalized mucosal cytokine activation. Journal of Acquired Immune Deficiency Syndromes: JAIDS 2004;37:1228-36.
30. McGowan I, Radford-Smith G, Jewell DP. Cytokine gene expression in HIV-infected intestinal mucosa. AIDS 1994;8:1569-75.
31. Luster AD. Chemokines--chemotactic cytokines that mediate inflammation. New England Journal of Medicine 1998;338:436-45.

32. MacDermott RP, Sanderson IR, Reinecker HC. The central role of chemokines (chemotactic cytokines) in the immunopathogenesis of ulcerative colitis and Crohn's disease. Inflammatory Bowel Diseases 1998;4:54-67.
33. Nabel G, Baltimore D. An inducible transcription factor activates expression of human immunodeficiency virus in T cells.[erratum appears in Nature 1990 Mar 8;344(6262):178]. Nature 1987;326:711-3.
34. Pantaleo G, Fauci AS. Immunopathogenesis of HIV infection. Annual Review of Microbiology 1996;50:825-54.
35. Bjerke K, Halstensen TS, Jahnsen F, Pulford K, Brandtzaeg P. Distribution of macrophages and granulocytes expressing L1 protein (calprotectin) in human Peyer's patches compared with normal ileal lamina propria and mesenteric lymph nodes. Gut 1993;34:1357-63.
36. Johne B, Fagerhol MK, Lyberg T, et al. Functional and clinical aspects of the myelomonocyte protein calprotectin. Molecular Pathology 1997;50:113-23.
37. Brun JG, Ulvestad E, Fagerhol MK, Jonsson R. Effects of human calprotectin (L1) on in vitro immunoglobulin synthesis. Scandinavian Journal of Immunology 1994;40:675-80.
38. Yui S, Mikami M, Yamazaki M. Induction of apoptotic cell death in mouse lymphoma and human leukemia cell lines by a calcium-binding protein complex, calprotectin, derived from inflammatory peritoneal exudate cells. Journal of Leukocyte Biology 1995;58:650-8.
39. Roseth AG, Aadland E, Jahnsen J, Raknerud N. Assessment of disease activity in ulcerative colitis by faecal calprotectin, a novel granulocyte marker protein. Digestion 1997;58:176-80.
40. Tibble J, Teahon K, Thjodleifsson B, et al. A simple method for assessing intestinal inflammation in Crohn's disease. Gut 2000;47:506-13.
41. Thjodleifsson B, Sigthorsson G, Cariglia N, et al. Subclinical intestinal inflammation: an inherited abnormality in Crohn's disease relatives?[see comment]. Gastroenterology 2003;124:1728-37.
42. Carballo-Dieguez A, Dolezal C, Bauermeister J, Ventuneac A, O'Brien W, Mayer K. Preference for gel over suppository as delivery vehicle for a rectal microbicide: results of a randomised, crossover acceptability trial among men who have sex with men. Sexually Transmitted Infections 2008;84:483-7.
43. Carballo-Dieguez A, Exner T, Dolezal C, Pickard R, Lin P, Mayer KH. Rectal microbicide acceptability: results of a volume escalation trial. Sexually Transmitted Diseases 2007;34:224-9.
44. CONRAD. Investigator's Brochure: Tenofovir Gel (GS-1278). Third Edition.
45. Malkovsky M, Newell A, Dalgleish AG. Inactivation of HIV by nonoxynol-9. Lancet 1988;1:645.
46. Polsky B, Baron PA, Gold JW, Smith JL, Jensen RH, Armstrong D. In vitro inactivation of HIV-1 by contraceptive sponge containing nonoxynol-9. Lancet 1988;1:1456.
47. Patton DL, Cosgrove Sweeney YT, Rabe LK, Hillier SL. Rectal applications of nonoxynol-9 cause tissue disruption in a monkey model. Sex Transm Dis 2002;29:581-7.
48. CONRAD. Investigator's Brochure: Universal Placebo Gel. 2005;Version 1.0.

49. Unpublished Work, C. Dezzutti.
50. Rohan LC, Moncla BJ, Kunjara Na Ayudhya RP, et al. In Vitro and Ex Vivo Testing of Tenofovir Shows It Is Effective As an HIV-1 Microbicide. PLoS ONE 2010;5:e9310;1-13.
51. Abner SR, Guenthner PC, Guarner J, et al. A human colorectal explant culture to evaluate topical microbicides for the prevention of HIV infection. Journal of Infectious Diseases 2005;192:1545-56.
52. Gilead, Sciences. Viread Investigator's Brochure. September 2006.
53. Gilead, Sciences. Viread® (tenofovir disoproxil fumarate) tablets. Package Insert November 2008.
54. Gilead, Sciences. Viread Company Core Safety Information. September 18, 2006.
55. Weber J, Chakraborty B, Weberova J, Miller MD, Quinones-Mateu ME. Diminished replicative fitness of primary human immunodeficiency virus type 1 isolates harboring the K65R mutation. Journal of Clinical Microbiology 2005;43:1395-400.
56. Dezzutti CS, James VN, Ramos A, et al. In vitro comparison of topical microbicides for prevention of human immunodeficiency virus type 1 transmission. Antimicrobial Agents & Chemotherapy 2004;48:3834-44.
57. Tien D, Schnaare RL, Kang F, et al. In vitro and in vivo characterization of a potential universal placebo designed for use in vaginal microbicide clinical trials. AIDS Research & Human Retroviruses 2005;21:845-53.
58. Creek MR. Pharmacokinetics and tissue distribution study of PMPA (GS-1278) following intravaginal administration in female New Zealand White Rabbits. SRI Report No B010-97.
59. Lund B, Weber S. Pharmacokinetics and distribution of intravaginal [14C] PMPA (GS-1278) following single dose administration to rats. Gilead Sciences Report No 96-DDM-1278-004.
60. Cranage M, Sharpe S, Herrera C, et al. Prevention of SIV Rectal Transmission and Priming of T Cell Responses in Macaques after Local Pre-exposure Application of Tenofovir Gel. PLoS Medicine 2008;5:1-13.
61. Rezk NL, Crutchley RD, Kashuba AD. Simultaneous quantification of emtricitabine and tenofovir in human plasma using high-performance liquid chromatography after solid phase extraction. Journal of Chromatography B: Analytical Technologies in the Biomedical & Life Sciences 2005;822:201-8.
62. Balzarini J, Van Herrewege Y, Vanham G. Metabolic activation of nucleoside and nucleotide reverse transcriptase inhibitors in dendritic and Langerhans cells. AIDS 2002;16:2159-63.
63. Delaney WEt, Ray AS, Yang H, et al. Intracellular metabolism and in vitro activity of tenofovir against hepatitis B virus. Antimicrobial Agents & Chemotherapy 2006;50:2471-7.
64. Robbins BL, Srinivas RV, Kim C, Bischofberger N, Fridland A. Anti-human immunodeficiency virus activity and cellular metabolism of a potential prodrug of the acyclic nucleoside phosphonate 9-R-(2-phosphonomethoxypropyl)adenine (PMPA), Bis(isopropyloxymethylcarbonyl)PMPA. Antimicrobial Agents & Chemotherapy 1998;42:612-7.

65. Fairchild D. 14-day vaginal tissue irritation and toxicity study of PMPA in rats. Report No M016-97.
66. Unpublished Work, R. Black.
67. Galen BT, Martin AP, Hazrati E, et al. A comprehensive murine model to evaluate topical vaginal microbicides: mucosal inflammation and susceptibility to genital herpes as surrogate markers of safety. Journal of Infectious Diseases 2007;195:1332-9.
68. Patton DL, Kidder GG, Sweeney YC, Rabe LK, Hillier SL. Effects of multiple applications of benzalkonium chloride and nonoxynol 9 on the vaginal epithelium in the pigtailed macaque (Macaca nemestrina). Am J Obstet Gynecol 1999;180:1080-7.
69. Sudol KM, Phillips DM. Relative safety of sexual lubricants for rectal intercourse. Sexually Transmitted Diseases 2004;31:346-9.
70. Miller CJ, Alexander NJ, Gettie A, Hendrickx AG, Marx PA. The effect of contraceptives containing nonoxynol-9 on the genital transmission of simian immunodeficiency virus in rhesus macaques. Fertil Steril 1992;57:1126-8.
71. Patton DL, Cosgrove Sweeney YT, Paul KJ. A summary of preclinical topical microbicide rectal safety and efficacy evaluations in a pigtailed macaque model. In Press: Sexually Transmitted Diseases 2008.
72. Patty's Industrial Hygiene and Toxicology Online. http://www3intersciencewileycom/cgi-bin/mrwhome/104554795/HOME; accessed 4/25/08:Section 43.4.
73. Guttner J, Klaus S, Heinecke H. Embryotoxizitat intraperitoneal applizierter Hydroxyethylcellulose bei der Maus. Anatomischer Anzeiger 1981;149:282-5.
74. Exner T, Correale J, Carballo-Dieguez A, et al. Women's anal sex practices: Implications for formulation and promotion of a rectal microbicide. AIDS Education and Prevention, in press 2008.
75. Patton DL. A Combination Microbicide Shows Differential Toxicity Compared With Standalone Products. In: Microbicides 2008; New Delhi.
76. Mayer KH, Maslankowski LA, Gai F, et al. Safety and tolerability of tenofovir vaginal gel in abstinent and sexually active HIV-infected and uninfected women. AIDS 2006;20:543-51.
77. Beigi R, Noguchi L, Macio I, et al. Maternal Single-Dose Pharmacokinetics and Placental Transfer of Tenofovir 1% Vaginal Gel Among Healthy Term Gravidas In: Microbicides 2010; 2010; Pittsburgh, PA; 2010.
78. CONRAD. Protocol #A04-099 (Study Report), Male Tolerance Study.
79. Hillier SL. Safety and acceptability of daily and coitally dependent use of 1% tenofovir over six months of use. In: Microbicides 2008; New Delhi.
80. Karim QA, Karim SS, Frohlich JA, et al. Effectiveness and Safety of Tenofovir Gel, an Antiretroviral Microbicide, for the Prevention of HIV Infection in Women. Science.
81. Fichorova RN, Tucker LD, Anderson DJ. The molecular basis of nonoxynol-9-induced vaginal inflammation and its possible relevance to human immunodeficiency virus type 1 transmission. Journal of Infectious Diseases 2001;184:418-28.
82. Mauck CK, Weiner DH, Creinin MD, Barnhart KT, Callahan MM, Bax R. A randomized Phase I vaginal safety study of three concentrations of C31G vs. Extra Strength Gynol II. Contraception 2004;70:233-40.

83. Van Damme L, Niruthisard S, Atisook R, et al. Safety evaluation of nonoxynol-9 gel in women at low risk of HIV infection. Aids 1998;12:433-7.
84. Hoffman IF, Taha TE, Padian NS, et al. Nonoxynol-9 100 mg gel: multi-site safety study from sub-Saharan Africa. AIDS 2004;18:2191-5.
85. Van Damme L, Ramjee G, Alary M, et al. Effectiveness of COL-1492, a nonoxynol-9 vaginal gel, on HIV-1 transmission in female sex workers: a randomised controlled trial. Lancet 2002;360:971-7.
86. Stafford MK, Ward H, Flanagan A, et al. Safety study of nonoxynol-9 as a vaginal microbicide: evidence of adverse effects. J Acquir Immune Defic Syndr Hum Retrovirol 1998;17:327-31.
87. Dreisbach R. Handbook of Poisoning. 9 ed. Los Altos, CA: Lang Medical Publications; 1977.
88. Schwartz JL, Ballagh SA, Kwok C, et al. Fourteen-day safety and acceptability study of the universal placebo gel. Contraception 2007;75:136-41.
89. Fuchs EJ, Lee LA, Torbenson MS, et al. Hyperosmolar sexual lubricant causes epithelial damage in the distal colon: potential implication for HIV transmission. Journal of Infectious Diseases 2007;195:703-10.
90. Halperin DT. Heterosexual anal intercourse: prevalence, cultural factors, and HIV infection and other health risks, Part I. AIDS Patient Care STDS 1999;13:717-30.
91. Schwandt M, Morris C, Ferguson A, Ngugi E, Moses S. Anal and dry sex in commercial sex work, and relation to risk for sexually transmitted infections and HIV in Meru, Kenya. Sex Transm Infect 2006;82:392-6.
92. Lard-Whiteford SL, Matecka D, O'Rear JJ, Yuen IS, Litterst C, Reichelderfer P. Recommendations for the nonclinical development of topical microbicides for prevention of HIV transmission: an update. J Acquir Immune Defic Syndr 2004;36:541-52.
93. Mauck C, Rosenberg Z, Van Damme L. Recommendations for the clinical development of topical microbicides: an update. Aids 2001;15:857-68.
94. McGowan I. Microbicides: a new frontier in HIV prevention. Biologicals 2006;34:241-55.
95. Phillips DM, Zacharopoulos VR. Nonoxynol-9 enhances rectal infection by herpes simplex virus in mice. Contraception 1998;57:341-8.
96. NIAID Study 97-101. Pharmacokinetic Study in Three Monkeys.
97. Carballo-Dieguez A, Dolezal C, Ventuneac A. Sexual Practices Assessment Schedule for Men who have Sex with Men (SPAS MSM 2002). Available from first author.
98. Gatto NM, Frucht H, Sundararajan V, Jacobson JS, Grann VR, Neugut AI. Risk of perforation after colonoscopy and sigmoidoscopy: a population-based study.[see comment]. Journal of the National Cancer Institute 2003;95:230-6.
99. Lee ML, Whitmore GA. Power and sample size for DNA microarray studies. Statistics in Medicine 2002;21:3543-70.
100. Du P, Kibbe WA, Lin SM. lumi: a pipeline for processing Illumina microarray. Bioinformatics 2008;24:1547-8.
101. Lin SM, Du P, Huber W, Kibbe WA. Model-based variance-stabilizing transformation for Illumina microarray data. Nucleic Acids Research 2008;36:e11.

102. Smyth GK. Linear Models and Empirical Bayes Methods For Assessing Differential Expressions. Statistical Applications in Genetics and Molecular Biology 2004;3:Article 3.
